# Geographically weighted temporally correlated logistic regression model

**DOI:** 10.1038/s41598-018-19772-6

**Published:** 2018-01-23

**Authors:** Yang Liu, Kwok-Fai Lam, Joseph T. Wu, Tommy Tsan-Yuk Lam

**Affiliations:** 1 Center of Influenza Research, State Key Laboratory of Emerging Infectious Diseases, The University of Hong Kong, Pokfulam, Hong Kong, China; 2 School of Public Health, The University of Hong Kong, Pokfulam, Hong Kong, China; 3 Department of Statistics and Actuarial Science, The University of Hong Kong, Pokfulam, Hong Kong, China

## Abstract

Detecting the temporally and spatially varying correlations is important to understand the biological and disease systems. Here we proposed a geographically weighted temporally correlated logistic regression (GWTCLR) model to identify such dynamic correlation of predictors on binomial outcome data, by incorporating spatial and temporal information for joint inference. The local likelihood method is adopted to estimate the spatial relationship, while the smoothing method is employed to estimate the temporal variation. We present the construction and implementation of GWTCLR and the study of the asymptotic properties of the proposed estimator. Simulation studies were conducted to evaluate the robustness of the proposed model. GWTCLR was applied on real epidemiologic data to study the climatic determinants of human seasonal influenza epidemics. Our method obtained results largely consistent with previous studies but also revealed certain spatial and temporal varying patterns that were unobservable by previous models and methods.

## Introduction

Regression analysis is widely used to study the correlation between dependent and independent variables. Some commonly used regression methods, e.g. linear regression, logistic regression and log linear regression, assumed that all sampling data have the uniform relationship with the factors but have a very stringent assumption of constant covariate effects. However, this assumption is not always true, particularly in complex multivariate systems. In public health and geographic information science, it is common to observe the data with dynamic patterns related to their geographical locations and sampling time, which are regarded as spatial and temporal non-stationarity. Such non-uniform relationships could be addressed and revealed by a varying coefficient model introduced by Hastie and Tibshirani^1^.

Multifactorial dynamic relationships are common in complex biological and disease systems at which some predictors cannot be observed or addressed easily. The usual logistic regression analysis assumes invariant coefficients and hence is inflexible to deal with such cases. For instance, seasonal dynamics of human influenza epidemics have been shown to associate with climatic factors such as temperature and humidity^2^. However, such association may change over time due to unaccounted factors including molecular evolution of the influenza viruses (e.g. emergence of mutants with a higher resistance against higher temperature) or other social events (e.g. mass gatherings, vaccine failure) that are often hard to measure and analyze with climatic factors. Therefore, the temporally and spatially varying coefficient models rationally surpass the invariant coefficient models with less bias. Yet, many of these unaccounted predictors are believed to follow the fundamental characteristics of spatial and temporal correlation. Our aim is to develop a model that can estimate the spatio-temporal pattern of these factors for accountable correlation.

Earliest temporally varying coefficient models arose from the analysis of longitudinal data commonly seen in medical and health cohort studies. Based on the simplest linear regression, a two-step estimation of functional linear regression method was proposed by Fan and Zhang^3^, where the collected longitudinal data is divided into different groups based on their sampling time and a linear regression analysis is performed within each group. In order to include information from the whole time period, smoothing method was used to refine the estimated regression coefficients attained from each group. In the generalized linear model’s setup, Cai *et al*.^4^ proposed a local likelihood method to deal with independent and identically distributed data by assigning a kernel weight to the likelihood of each observation. Şentürk^5^ further extended the local likelihood method to accommodate longitudinal data. Dong *et al*.^6^ extended the two-step estimation method to logistic regression to analyze binary data. This method is similar to the one proposed by Fan and Zhang^3^ but their raw estimates are more susceptible to bias which results in the requirement of large sample size.

Spatially varying coefficient model is popular in geographic information science. The spatial feature is depicted by the location coordinates, and it is natural to assume an intrinsic difference in relationships between different variables over the spatial unit. As initial and fundamental works, Brunsdon *et al*.^7^ and Fotheringham *et al*.^8^ proposed the geographically weighted regression (GWR) analysis for variables with geographically non-stationary coefficients. They used linear regression with a weighted least squares approach by assigning a geographical weight to each observation corresponding to the distance between the observation’s location and the location where the regression coefficient is being inferred. The fundamental assumption of GWR is Tobler’s first law of geography: “everything is related to everything else, but near things are more related than distant things”^9^, and hence the weight decreases toward zero when distance goes to infinity. Nakaya *et al*.^10^ proposed a natural extension of GWR model to geographically weighted Poisson regression (GWPR) model for count data. Since the usual least squares estimator is not available for the generalized linear model, a variant of local likelihood approach is used. By applying the iteratively reweighted least squares, they further deduced the asymptotic properties of their proposed estimator. They are also the first to propose the semi-parametric GWPR model which allows some of the variables to be invariant.

Some studies proposed to incorporate temporal non-stationarity into GWR framework recently to account for the temporal variation. Huang *et al*.^11^ proposed the geographically and temporally weighted regression (GTWR) model as an extended version of GWR model to integrate both temporal and spatial information into the analysis by treating time as the third dimension in addition to the location and distance in a straightforward manner to calculate the weight. To further address the possible correlation in the cases of regular sampling times, Wu *et al*.^12^ proposed the geographically and temporally weighted autoregressive model by applying an autoregressive model within the linear function and proposed a two-stage least squares estimation method for the model. Fotheringham *et al*.^13^ pointed out that treating time as the third dimension of location is not entirely appropriate since time and geographical information are measured in different scales. They modified the GTWR model by defining the weight function as a product of two weight functions calculated from temporal and spatial information respectively.

There is an increasing interest in modeling spatial and temporal data especially in public health. Hu *et al*.^14^ used GWR model to investigate the determinants for the incidence of hand, foot and mouth disease. Lin and Wen^15^ used GWR model to explore the factors that influence the dengue disease incidence. Tsai and Yeh^16^ used GWR model to identify the possible association for scrub typhus disease. As laboratory testing or diagnosis for disease surveillance often generates binary data (e.g. positive or negative for a certain pathogen in the detection assay), logistic regression model for these binary outcome data with spatial and temporal information has particular value study factors driving the presence or absence of the disease. For instance, the geographically weighted logistic regression (GWLR) were considered by Wu *et al*.^17^ and Zhou *et al*.^18^. However, there is no a similar logistic regression model to deal with those disease detection data and considering both the geographical and temporal variation of the correlation.

To this end, we propose a flexible geographically weighted temporally correlated logistic regression (GWTCLR) model as a natural extension of GWLR model for the analysis of binomial spatial and temporal data. It incorporates both spatial and temporal information by introducing the spatio-temporal varying coefficients to the logistic regression model, which accommodates the potential temporal correlation among the observations with flexible choices of correlation structures. For a specific location, we employ local likelihood method to maximize a geographically weighted likelihood with weight related to the geographical relationship in the spatial variant part to obtain the raw estimates for the coefficients. In order to include information from the entire time period, we use smoothing method to attain the refined estimates for any particular location in the temporal variant part. Using this method, we can attain regression coefficients of other closely related locations at any time within the observation period, and hence a plot of the coefficient over time can be constructed to visualize the temporal variation of the coefficient estimates. To accommodate the potential correlation among the longitudinal data with auto-correlation structure as a special case, the concept of tetrachoric correlation proposed by Lecessie and Vanhouwelingen^19^ is adopted in the model. Simulation study and application on real influenza epidemiological data were conducted to assess and demonstrate the robustness and utilization of the proposed method.

## Methods

Suppose the data are collected from $M$ distinct locations, each with a geographical coordinate (*u*_*i*_,*v*_*i*_) on *T*_*i*_ occasions (for *i* = 1, …, *M*). Moreover *S*_*i*,*t*_ samples are collected from location *i* at the *t*^th^ occasion (*t* = 1, …, *T*_*i*_) with sampling times $({t}_{1},{t}_{2},\ldots ,{t}_{{T}_{i}})$. Define the observed data pairs as (*X*_*i*,*t*,*j*_, *Y*_*i*,*t*,*j*_) where *Y*_*i*,*t*,*j*_ is a binary dependent variable, *X*_*i*,*t*,*j*_ is a vector of independent variables for *j* = 1, …, *S*_*i*,*t*_, and it is assumed that *X*_*i*,*t*,*j*_ = *X*_*i*,*t*_. We assume *Y*_*i*,*t*,*j*_ = 1 if the underlying event of interest is observed and *Y*_*i*,*t*,*j*_ = 0 otherwise and let

1 $$P({Y}_{i,t,j}=1)={\pi }_{i,t,j}.$$

We now assume that the coefficients of the independent variables in the marginal logistic regression model are spatially and temporally non-stationary. That is

2 $${\rm{P}}({Y}_{i,t,j}=1)={\pi }_{i,t,j}=\frac{\exp ({X}_{i,t}\beta ({u}_{i},{v}_{i},t))}{1+\exp ({X}_{i,t}\beta ({u}_{i},{v}_{i},t))},$$

where *β*(*u*_*i*_, *v*_*i*_, *t*) is a vector of regression coefficients.

### Temporal Correlation Structure

For a fixed location and time, each sample is assumed to be identical and independently distributed, under condition on their covariates, that is:

3 $${Y}_{i,t,j}\perp {Y}_{a,b,c}|({X}_{i,t},{X}_{a,b})\,{\rm{if}}\,{\rm{only}}\,{\rm{if}}\,\{i\ne a\}\cup (\{i=a\}\cap \{t=b\}).$$

The following discussed correlations are correlations conditional on the given the covariates, and we will omit the conditional notations for simplicity.

The tetrachoric correlation approach, proposed by Lecessie and Vanhouwelingen^19^, is adopted to accommodate the potential association among the binary variables. Additional information for the tetrachoric correlation can be found in Supplementary Methods. Assume a weakly stationary tetrachoric correlated samples within location $i$ and denote

4 $${\rm{corr}}({Y}_{i,{t}_{1},j},{Y}_{i,{t}_{2},k})={c}_{i}(|{t}_{2}-{t}_{1}|),{\rm{when}}\,{t}_{1}\ne {t}_{2}.$$

It is natural to assume a temporally decreasing correlation structure for all locations. In the cases with irregular sampling times, examples are:

(A) Linear Correlation Structure
  5 $${\rm{corr}}({Y}_{i,{t}_{1},j},{Y}_{i,{t}_{2},k})={c}_{i}(|{t}_{2}-{t}_{1}|)=\{\begin{array}{cc}1-(\frac{|{t}_{2}-{t}_{1}|}{{\rho }_{i}}), & {\rm{if}}\,|{t}_{2}-{t}_{1}| < {\rho }_{i};\\ 0, & {\rm{otherwise}}\end{array},$$
  where ${\rho }_{i}$ is the temporal correlation parameter.
(B) Gaussian Correlation Structure
  6 $${\rm{corr}}({Y}_{i,{t}_{1},j},{Y}_{i,{t}_{2},k})={c}_{i}(|{t}_{2}-{t}_{1}|)=\exp \{-{(\frac{{t}_{2}-{t}_{1}}{{\rho }_{i}})}^{2}\}.$$
  In the cases with discrete regular sampling times, a natural choice is
(C) AR(1) Correlation Structure:

7 $${\rm{corr}}({Y}_{i,{t}_{1},j},{Y}_{i,{t}_{2},k})={c}_{i}(|{t}_{2}-{t}_{1}|)={{\rho }_{i}}^{|{t}_{2}-{t}_{1}|},$$

where −1 ≤ *ρ*_*i*_ ≤ 1 is the tetrachoric correlation when temporal distance is 1.

Regardless of the choice of correlation structure, there is only one temporal correlation parameter, namely ${\rho }_{i}$, involved to describe the temporal correlation which can be estimated by maximum likelihood estimation (MLE) principle. However, it is difficult to give an explicit expression of the estimator. Note that this parameter is a scalar and is often constrained in a specific interval that can be estimated easily by fixing the regression parameters at the most updated estimated values.

### Model Construction

In this subsection, we will focus on estimating the regression coefficients for location $i$ at time t. Suppose the temporal correlation parameter ${\rho }_{i}$ is known.

It is natural to assume that the similarity between coefficients from different time points decreases with their temporal distance. Thus, for location *i*, we define a *τ*-nearest temporal set, and we assume all coefficients *β* are the same within each set. Here *τ* is a bandwidth which can be chosen by prior knowledge. It should be noted that the choice of *τ* depends on the smoothing property of *β*(*u*_*i*_, *v*_*i*_, *t*) related to *t*. For a large *τ*, bias may be introduced. However for a small *τ*, fewer samples may be involved which results in larger variance of the estimator. For time *t*_*k*_, its *τ*-nearest temporal set is defined as

8 $$T[{t}_{k}]=\{t\in \{{t}_{1},\ldots ,{t}_{{T}_{i}}\}||t-{t}_{k}|\le \tau \}.$$

For each *Y*_*i*,*t*,*j*_, there is a corresponding latent variable *Z*_*i*,*t*,*j*_, with ${Y}_{i,t,j}={1}_{\{{Z}_{i,t,j} < {{\rm{\Phi }}}^{-1}({\pi }_{i,t,j})\}}$, where the marginal distribution of *Z*_*i*,*t,*j_, is a standard normal distribution, denoted by ${\rm{\Phi }}$. Define *Y*_*i*,*tk*_ be a vector with elements *Y*_*i*,*t*,*j*_ if *t* ∈ *T*[*t*_*k*_], then the elements of *Y*_*i*,*tk*_ are the random variables used to construct the spatio-temporal local likelihood function for the raw estimation of the regression coefficients for location *i* and time *t*_*k*_. Let ${{\rm{\Sigma }}}_{i,{t}_{k}}={\rm{corr}}({Y}_{i,{t}_{k}})$ be the corresponding tetrachoric correlation matrix of *Y*_*i*,*tk*_ and ${N}_{i,{t}_{k}}={\rm{\dim }}({Y}_{i,{t}_{k}})$, the length of vector *Y*_*i*,*t*,*k*_.

For simplicity, for fixed location *i* and time *t*_*k*_, we re-define the elements of ${Y}_{i,{t}_{k}}$ as ${Y}_{1}.{Y}_{2},\ldots \ldots ,{Y}_{{N}_{i,{t}_{k}}}$, the corresponding latent variable ${Z}_{i,{t}_{k}}$ as $({Z}_{1},{Z}_{2},\ldots \ldots ,{Z}_{{N}_{i,{t}_{k}}})$, and the corresponding independent variable matrix ${X}_{i,{t}_{k}}$ as $({X}_{1}.{X}_{2},\ldots \ldots ,{X}_{{N}_{i,{t}_{k}}})$. The marginal distribution of ${Y}_{i}$ is specified by a Bernoulli distribution with *P*(*Y*_*i*_ = 1|*X*_*i*_) = *π*_*i*_ where *π*_*i*_ = (exp(*X*_*i*_*β*))/(1 + exp(*X*_*i*_*β*)). Then the spatio-temporal local log-likelihood function for location i and time ${t}_{k}$ given the observed vector ${Y}_{i,{t}_{k}}$ is

9 $${l}_{i,{t}_{k}}=\,\mathrm{log}\,{L}_{i,{t}_{k}}=\,\mathrm{log}(\int \int \ldots \ldots \int {\phi }_{{N}_{i,{t}_{k}}}({Z}_{i,{t}_{k}},0,{{\rm{\Sigma }}}_{i,{t}_{k}})d{Z}_{i,{t}_{k}}),$$

where ${\phi }_{{N}_{i,{t}_{k}}}$ is the probability density function of multivariate normal distribution with dimension ${N}_{i,{t}_{k}}$, mean 0 and variance ${{\rm{\Sigma }}}_{i,{t}_{k}}$, the lower and upper limits of the integration with respect to ${Z}_{i,k}$ are respectively −∞ and Φ^−1^(*π*_*j*_) if ${Y}_{j}=1$; and are Φ^−1^(*π*_*j*_) and +∞, respectively if *Y*_*j*_ = 0.

Now, we focus on providing the raw estimate for $\beta ({u}_{i},{v}_{i},{t}_{k})$, the regression parameter associated with location $i$ and time ${t}_{k}$. We can attain the spatio-temporal local likelihood for each location at time ${t}_{k}$. Then what we need to do is to assign a weight to each spatio-temporal local log-likelihood defined by equation (9). A variant local likelihood principle is used, noted that this principle is similar to the weighted likelihood introduced in literature^20^. A geographical weight function is adopted and based on the first law of geography, we believe that near locations have more impact on the estimate, and hence the weight of nearer locations’ local log-likelihoods should be higher. For those distant locations, their impacts are presumably smaller or could even be ignored. Here we adopt the Gaussian distance decay-based weighting function proposed by Brunsdon *et al*.^7^. The function is defined as *W*_*ij*_ = exp(−((*d*_*ij*_)/(*h*))^2^), where *d*_*ij*_ is the distance between location i and location j and h is the geographical bandwidth parameter. The temporal local log-likelihood function for location $i$ and time ${t}_{k}$ is

10 $${\bar{l}}_{i,{t}_{k}}=\sum _{j=1}^{M}{W}_{ij}{l}_{j,{t}_{k}},$$

and let $\hat{b}({u}_{i},{v}_{i},{t}_{k})$ be the raw estimate for $\beta ({u}_{i},{v}_{i},{t}_{k})$, we have

11 $$\hat{b}({u}_{i},{v}_{i},{t}_{k})=\mathop{{\rm{argmax}}}\limits_{\beta }{\bar{l}}_{i,{t}_{k}}.$$

The spatio-temporal local log-likelihood is rather complicated and difficult to differentiate. Here we use the pseudo-likelihood introduced in literature^19^ to approximate the true likelihood, additional information for the pseudo-likelihood and its derivative can be found in Supplementary Methods. Therefore, the raw estimate for $\beta ({u}_{i},{v}_{i},{t}_{k})$ is given by

12 $$\hat{b}({u}_{i},{v}_{i},{t}_{k})=\mathop{{\rm{argmax}}}\limits_{\beta }\,{\bar{l}}_{i,{t}_{k}}^{pse}.$$

Since a weakly stationary correlation is assumed, we use samples from the whole period to estimate temporal correlation parameter $\rho$. As $\rho$ is constrained in a specific interval, we search $\rho$ within the region with a predefined step size. We attain the raw estimates for *β* and calculate the log-likelihoods under different values of $\rho$, and the ML estimate for $\rho$ is approximated by the one which gives the highest log-likelihood.

It is noted that the raw estimate $\hat{b}({u}_{i},{v}_{i},{t}_{k})$ should reflect $\beta ({u}_{i},{v}_{i},{t}_{k})$ in certain extent. Since we only use samples with sampling time from the $\tau$-nearest temporal set $T[{t}_{k}]$, the raw estimate is incomplete. In order to include information from the whole period, we will refine $\hat{b}({u}_{i},{v}_{i},{t}_{k})$ using the nonparametric local polynomials method^21^. For the raw estimate of the set of location $i$,

13 $$\hat{b}=(\hat{b}({u}_{i},{v}_{i},{t}_{1}),\,\hat{b}({u}_{i},{v}_{i},{t}_{2}),\ldots \ldots ,\hat{b}({u}_{i},{v}_{i},{t}_{T})),$$

and let ${\hat{b}}_{m}=({\hat{b}}_{m}({u}_{i},{v}_{i},{t}_{1}),{\hat{b}}_{m}({u}_{i},{v}_{i},{t}_{2}),\ldots \ldots ,{\hat{b}}_{m}({u}_{i},{v}_{i},{t}_{T}))$ be the *m*^th^ row of $\hat{b}$. Given a kernel function $K$, bandwidth $h$ and order $p$, we fit $\hat{b}$ with the time to get the refined estimate ${\hat{\beta }}_{m}({u}_{i},{v}_{i},{\rm{t}})$ for any time $t$ within the period. We have

14 $${\hat{\beta }}_{m}({u}_{i},{v}_{i},{\rm{t}})=\widehat{{\alpha }_{0}}+\sum _{r=1}^{p}\widehat{{\alpha }_{r}}{t}^{r},$$

where $$\widehat{{\alpha }_{0}},\widehat{{\alpha }_{1}},\ldots \ldots ,\widehat{{\alpha }_{p}}$$ minimize

15 $$\sum _{n=1}^{T}K(\frac{t-{t}_{n}}{h}){({\hat{b}}_{m}({u}_{i},{v}_{i},{t}_{n})-\widehat{{\alpha }_{0}}-\sum _{r=1}^{p}\widehat{{\alpha }_{r}}{{t}_{n}}^{r})}^{2}.$$

Let $B$ be the design matrix and ${{\rm{\Omega }}}_{t}$ be a diagonal matrix with diagonal elements $K(\frac{t-{t}_{n}}{h})$, and let *B*(*t*) = (1, *t*^1^, *t*^2^, …, *t*^r^), we have the refined estimate of *β*(*u*_*i*_, *v*_*i*_, t) given by

16 $$\hat{\beta }({u}_{i},{v}_{i},{\rm{t}})={[B(t){{({B}^{T}{{\rm{\Omega }}}_{t}B)}^{-1}{B}^{T}{{\rm{\Omega }}}_{t}\hat{b}}^{T}]}^{T},$$

and $\hat{\beta }({u}_{i},{v}_{i},{\rm{t}})$ is termed the GWTCLR estimator of $\beta ({u}_{i},{v}_{i},{\rm{t}}).$

### Geographical Bandwidth Selection

The spatial impact and temporal impact are assumed to be independent. Therefore, we first assume no temporal correlation and hence use all samples from the whole period to estimate a geographical bandwidth. A geographically weighted logistic regression (GWLR)^8^ is used, where a Poisson approximation to the binomial distribution can be considered as an alternative, and small sample bias corrected AIC (AICc)^22^ or BIC is used to choose the bandwidth *h* of the geographical weight function. This procedure can be done in GWR 4.0, which is available for estimating the bandwidth. More discussion about the choice of bandwidth can be found in literature^8^.

## Asymptotic Properties and Covariance

In order to give the covariance of GWTCLR estimator, the asymptotic properties were studied. The raw estimate $\hat{b}({u}_{i},{v}_{i},{t}_{k})$ for $\beta ({u}_{i},{v}_{i},{t}_{k})$ which is derived from

17 $${\bar{l}}_{i,{t}_{k}}(\beta )=\sum _{j=1}^{M}{W}_{ij}{l}_{j,{t}_{k}}(\beta |{Y}_{j,{t}_{k}},{X}_{j,{t}_{k}})=\sum _{j=1}^{M}{W}_{ij}\,\mathrm{log}({f}_{j,{t}_{k}}({Y}_{j,{t}_{k}}|\beta ,{X}_{j,{t}_{k}})),$$

where the probability density of ${Y}_{j,{t}_{k}}$ given $\beta$ is as defined in equation (9) as

18 $${f}_{j,{t}_{k}}({Y}_{j,{t}_{k}}|\beta ,{X}_{j,{t}_{k}})={L}_{j,{t}_{k}}.$$

Herein, ${E}_{0}\,{\rm{and}}\,Va{r}_{0}$ denote the expectation and variance under the probability space based on the true parameter. Without loss of generality, we focus on location $i$ and time ${t}_{k}$, and suppose the temporal correlation parameter is known.

We have the following theorems given the necessary assumptions.

### ***Assumption 1***.

*Let* ${f}_{j,{t}_{k}}({Y}_{j,{t}_{k}}|\beta ,{X}_{j,{t}_{k}})$ *be the probability density of* ${Y}_{j,{t}_{k}}$ *given β and* ${X}_{j,{t}_{k}}$. *Assume for all β* ∈ *B*, *where B is the open parameter set that contains the true parameter*. *When the bandwidth of the geographical weight function is small enough*, *and when β*(*u*_*i*_, *v*_*i*_, *t*_*k*_) *is smooth related to u*_*i*_, *v*_*i*_, *then the following is always true:*

19 $$\frac{1}{{M}^{2}}\sum _{j=1}^{M}Va{r}_{0}({W}_{ij}\,\mathrm{log}({f}_{j,{t}_{k}}({Y}_{j,{t}_{k}}|\beta ,{X}_{j,{t}_{k}}))) < \infty ,\forall M > 0,\,\forall \beta \in B.$$

### ***Theorem 1.***

(*Large Sample and Small Bandwidth Asymptotic Consistency*). *When sample size is large enough and the bandwidth of the geographical weight function is small enough*, *under assumption 1*, *score function* (*dl*_*M*_(*β*))/(*dβ*) = 0 *almost surely has a solution and this solution asymptotically converges to the real parameter in probability*.

### ***Proof.***

*See Supplementary Methods*.

### ***Assumption 2.***

*Let the parameter β be a p-dimensional vector*, *β* = (*β*_1_,*β*_2_,…,*β*_*p*_)^*T*^ and suppose the following conditions are always true:

i. *The probability density* ${f}_{j,{t}_{k}}({Y}_{j,{t}_{k}}|{\beta },{X}_{j,{t}_{k}})$ *is twice differentiable with respect to* $\beta$ *for every j*.
ii. *Let* $\overline{{{\rm{\Sigma }}}_{M}}=\frac{1}{M}Va{r}_{0}(\frac{\partial {\bar{l}}_{i,{t}_{k}}(\beta )}{\partial \beta }{|}_{\beta =\beta ({u}_{i},{v}_{i},{t}_{k})})$, *by geographically independent*, *we have*
  20 $$\overline{{{\rm{\Sigma }}}_{M}}=\frac{1}{M}\sum _{j=1}^{M}Va{r}_{0}({W}_{ij}\frac{\frac{\partial {f}_{j,{t}_{k}}({Y}_{j,{t}_{k}}|\beta ,{X}_{j,{t}_{k}})}{\partial \beta }{|}_{\beta =\beta ({u}_{i},{v}_{i},{t}_{k})}}{{f}_{j,{t}_{k}}({Y}_{j,{t}_{k}}|\beta ({u}_{i},{v}_{i},{t}_{k}),{X}_{j,{t}_{k}})})\mathop{\to }\limits^{M\to +\infty }{\rm{\Sigma }},$$
  where $\Sigma$ is a finite, positive definite matrix.
iii. *For every j*, *we have:*
  21 $$\mathop{\mathrm{lim}}\limits_{M\to +\infty }{(M\overline{{{\rm{\Sigma }}}_{M}})}^{-1}Va{r}_{0}({W}_{ij}\frac{\frac{\partial {f}_{j,{t}_{k}}({Y}_{j,{t}_{k}}|\beta ,{X}_{j,{t}_{k}})}{\partial \beta }{|}_{\beta =\beta ({u}_{i},{v}_{i},{t}_{k})}}{{f}_{j,{t}_{k}}({Y}_{j,{t}_{k}}|\beta ({u}_{i},{v}_{i},{t}_{k}),{X}_{j,{t}_{k}})})=0.$$
iv. *For every* $1\le i < j\le p$ *and M* > *0*, *the second order derivative of* ${\bar{l}}_{i,{t}_{k}}(\beta )$ *satisfies:*

22 $$\frac{1}{{M}^{2}}\sum _{j=1}^{M}Va{r}_{0}({W}_{ij}\frac{{\partial }^{2}\,\mathrm{log}({f}_{j,{t}_{k}}({Y}_{j,{t}_{k}}|\beta ,{X}_{j,{t}_{k}}))}{\partial {\beta }_{i}\partial {\beta }_{j}}{|}_{\beta =\beta ({u}_{i},{v}_{i},{t}_{k})}) < +\infty ;$$

and

23 $$\mathop{\mathrm{lim}}\limits_{M\to \infty }\frac{1}{M}\sum _{j=1}^{M}{E}_{0}({W}_{ij}\frac{{\partial }^{2}\,\mathrm{log}({f}_{j,{t}_{k}}({Y}_{j,{t}_{k}}|\beta ,{X}_{j,{t}_{k}}))}{\partial {\beta }_{i}\partial {\beta }_{j}}{|}_{\beta =\beta ({u}_{i},{v}_{i},{t}_{k})})=I,$$

*where I is a finite*, *positive definite matrix*.

### ***Theorem 2.***

(*Large Sample and Small Bandwidth Asymptotic Normality*). *When sample size is large enough and the bandwidth of the geographical weight function is small enough*, *under assumption 1 and assumption 2*, *the raw estimator of GWTCLR follows a normal distribution asymptotically as follows:*

24 $$\sqrt{M}(\hat{b}({u}_{i},{v}_{i},{t}_{k})-\beta ({u}_{i},{v}_{i},{t}_{k})) \sim AN(0,M{{I}_{M}}^{-1}{{\rm{\Sigma }}}_{M}{{I}_{M}}^{-1})$$

where

25 $$\begin{array}{c}{I}_{M}={E}_{0}(\frac{{{\rm{\partial }}}^{2}{\bar{l}}_{i,{t}_{k}}(\beta )}{{\rm{\partial }}{\beta }^{2}}{|}_{\beta =\beta ({u}_{i},{v}_{i},{t}_{k})}),\\ {{\rm{\Sigma }}}_{M}=\sum _{j=1}^{M}Va{r}_{0}({W}_{ij}\frac{\frac{{\rm{\partial }}{f}_{j,{t}_{k}}({Y}_{j,{t}_{k}}|\beta ,{X}_{j,{t}_{k}})}{{\rm{\partial }}\beta }{|}_{\beta =\beta ({u}_{i},{v}_{i},{t}_{k})}}{{f}_{j,{t}_{k}}({Y}_{j,{t}_{k}}|\beta ({u}_{i},{v}_{i},{t}_{k}),{X}_{j,{t}_{k}})}).\end{array}$$

### ***Proof***.

*See Supplementary Methods*.

By Theorem 2, asymptotically, we have

26 $$\begin{array}{rcl}Var(\hat{b}({u}_{i},{v}_{i},{t}_{k})) & = & {{I}_{M}}^{-1}{{\rm{\Sigma }}}_{M}{{I}_{M}}^{-1}\\  & = & {[{E}_{0}(\frac{{\partial }^{2}{\bar{l}}_{i,{t}_{k}}(\beta )}{\partial {\beta }^{2}}{|}_{\beta =\beta ({u}_{i},{v}_{i},{t}_{k})})]}^{-1}\\  &  & \times \,(\sum _{j=1}^{M}Va{r}_{0}({W}_{ij}\frac{\partial {l}_{j,{t}_{k}}(\beta |{Y}_{j,{t}_{k}},{X}_{j,{t}_{k}})}{\partial \beta }{|}_{\beta =\beta ({u}_{i},{v}_{i},{t}_{k})}))\\  &  & \times \,{[{E}_{0}(\frac{{\partial }^{2}{\bar{l}}_{i,{t}_{k}}(\beta )}{\partial {\beta }^{2}}{|}_{\beta =\beta ({u}_{i},{v}_{i},{t}_{k})})]}^{-1}.\end{array}$$

By the same method in the proof of Theorem 2

27 $$\begin{array}{l}Cov(\hat{b}({u}_{i},{v}_{i},{t}_{n}),\hat{b}({u}_{i},{v}_{i},{t}_{m}))\\ \begin{array}{ll}\quad = & {[{E}_{0}(\frac{{\partial }^{2}{\bar{l}}_{i,{t}_{n}}(\beta )}{\partial {\beta }^{2}}{|}_{\beta =\beta ({u}_{i},{v}_{i},{t}_{n})})]}^{-1}\\  & \times (\sum _{j=1}^{M}Co{v}_{0}({W}_{ij}\tfrac{\partial {l}_{j,{t}_{n}}(\beta |{Y}_{j,{t}_{n}},{X}_{j,{t}_{n}})}{\partial \beta }{|}_{\beta =\beta ({u}_{i},{v}_{i},{t}_{n})},{W}_{ij}\tfrac{\partial {l}_{j,{t}_{m}}(\beta |{Y}_{j,{t}_{m}},{X}_{j,{t}_{m}})}{\partial \beta }{|}_{\beta =\beta ({u}_{i},{v}_{i},{t}_{m})}))\\  & \times {[{E}_{0}(\frac{{\partial }^{2}{\bar{l}}_{i,{t}_{m}}(\beta )}{\partial {\beta }^{2}}{|}_{\beta =\beta ({u}_{i},{v}_{i},{t}_{m})})]}^{-1}.\end{array}\end{array}$$

Then we can estimate them by pseudo-likelihood as follows

28 $$\begin{array}{l}\widehat{Var}(\hat{b}({u}_{i},{v}_{i},{t}_{k}))\\ \begin{array}{ll}\quad = & {[E(\frac{{\partial }^{2}{\bar{l}}_{i,{t}_{k}}^{pse}(\beta )}{\partial {\beta }^{2}}{|}_{\beta =\hat{b}({u}_{i},{v}_{i},{t}_{k})})]}^{-1}\\  & \times (\sum _{j=1}^{M}{{W}_{ij}}^{2}(\tfrac{\partial {l}_{j,{t}_{k}}^{pse}(\beta |{Y}_{j,{t}_{k}},{X}_{j,{t}_{k}})}{\partial \beta }{|}_{\beta =\hat{b}({u}_{i},{v}_{i},{t}_{k})}){(\tfrac{\partial {l}_{j,{t}_{k}}^{pse}(\beta |{Y}_{j,{t}_{k}},{X}_{j,{t}_{k}})}{\partial \beta }{|}_{\beta =\hat{b}({u}_{i},{v}_{i},{t}_{k})})}^{T})\\  & \times {[E(\frac{{\partial }^{2}{\bar{l}}_{i,{t}_{k}}^{pse}(\beta )}{\partial {\beta }^{2}}{|}_{\beta =\hat{b}({u}_{i},{v}_{i},{t}_{k})})]}^{-1};\end{array}\end{array}$$

and

29 $$\begin{array}{l}\widehat{Cov}(\hat{b}({u}_{i},{v}_{i},{t}_{n}),\hat{b}({u}_{i},{v}_{i},{t}_{m}))\\ \begin{array}{ll}\quad = & {[E(\frac{{\partial }^{2}{\bar{l}}_{i,{t}_{n}}^{pse}(\beta )}{\partial {\beta }^{2}}{|}_{\beta =\hat{b}({u}_{i},{v}_{i},{t}_{n})})]}^{-1}\\  & \times (\sum _{j=1}^{M}{{W}_{ij}}^{2}(\tfrac{\partial {l}_{j,{t}_{n}}^{pse}(\beta |{Y}_{j,{t}_{n}},{X}_{j,{t}_{n}})}{\partial \beta }{|}_{\beta =\hat{b}({u}_{i},{v}_{i},{t}_{n})}){(\tfrac{\partial {l}_{j,{t}_{m}}^{pse}(\beta |{Y}_{j,{t}_{m}},{X}_{j,{t}_{m}})}{\partial \beta }{|}_{\beta =\hat{b}({u}_{i},{v}_{i},{t}_{m})})}^{T})\\  & \times {[E(\frac{{\partial }^{2}{\bar{l}}_{i,{t}_{m}}^{pse}(\beta )}{\partial {\beta }^{2}}{|}_{\beta =\hat{b}({u}_{i},{v}_{i},{t}_{m})})]}^{-1}.\end{array}\end{array}$$

Now, for the refined estimate $\hat{\beta }({u}_{i},{v}_{i},{t}_{k})$, it has the following general expression

30 $$\hat{\beta }({u}_{i},{v}_{i},{t}_{k})=\sum _{j=1}^{T}w({t}_{k},{t}_{j})\hat{b}({u}_{i},{v}_{i},{t}_{j}),$$

where function $w({t}_{k},{t}_{j})$ is deduced from the non-parametric fit. So we can finally get the variance estimate as

31 $$\begin{array}{rcl}\widehat{{\rm{Var}}}(\hat{\beta }({u}_{i},{v}_{i},{t}_{k})) & = & \sum _{j=1}^{T}{[w({t}_{k},{t}_{j})]}^{2}\widehat{{\rm{Var}}}(\hat{b}({u}_{i},{v}_{i},{t}_{j}))\\  &  & +\,2\sum _{1\le n < m\le T}w({t}_{k},{t}_{n})w({t}_{k},{t}_{m})\widehat{{\rm{Cov}}}(\hat{b}({u}_{i},{v}_{i},{t}_{n}),\hat{b}({u}_{i},{v}_{i},{t}_{m})).\end{array}$$

Note that, we point out that this variance estimate is based on large sample and small bandwidth, and we can further give a raw asymptotic 95% confidence interval for $\beta ({u}_{i},{v}_{i},{t}_{k})$ by

32 $$\hat{\beta }({u}_{i},{v}_{i},{t}_{k})\pm {{\rm{\Phi }}}^{-1}(0.975)\sqrt{\widehat{Var}(\hat{\beta }({u}_{i},{v}_{i},{t}_{k}))}.$$

## Simulation Studies

### Simulation Design

We conducted simulation studies to evaluate the validity of the proposed method. Our simulation contains 3 coefficient functions for two independent variables ${X}_{1},\,{X}_{2}$ and the y-intercept. The ${X}_{1}$ is generated from Uniform(−5, 5), ${X}_{2}$ is generated from Uniform(−50, 50). To account for different cases, we set three coefficient functions (a spatio-temporally fixed ${\beta }_{0}$, a spatio-temporally varying ${\beta }_{1}$ and a spatially fixed but temporally varying ${\beta }_{2}$). For location $(u,v)$ and time t, the varying coefficient functions are

33 $$\{\begin{array}{c}{\beta }_{0}=1,\\ {\beta }_{1}(u,v,t)=0.001\times sgn(u)\times {u}^{2}+0.001\times sgn(v)\times {v}^{2}+0.05\times sin(2\pi ((t-1)/20)+\pi /2),\\ {\beta }_{2}(t)=0.035\,{\rm{l}}{\rm{o}}{\rm{g}}(\pi ((t-1)/20)+\pi /2)-\mathrm{0.035.}\end{array}$$

Two data sets, each of size 10 × 10 × 21, are generated regularly on a square grid with arbitrary distance unit. For data set A, location $(u,v)$ ranges from 8.2 to 11.8 with a step size of 0.4 for u and v. For data set B, location $(u,v)$ ranges from −11.8 to −8.2 with a step size of 0.4 for u and v. Time ranges from 1 to 21 with a step size of 1 for data set A and B. For each location $(u,v)$ and time t, we simulate 500 binary data with probability P as

34 $${\rm{P}}=\frac{\exp ({\beta }_{0}+{\beta }_{1}(u,v,t){X}_{1}+{\beta }_{2}(t){X}_{2})}{1+\exp ({\beta }_{0}+{\beta }_{1}(u,v,t){X}_{1}+{\beta }_{2}(t){X}_{2})}.$$

### Result

**Figure 1:**
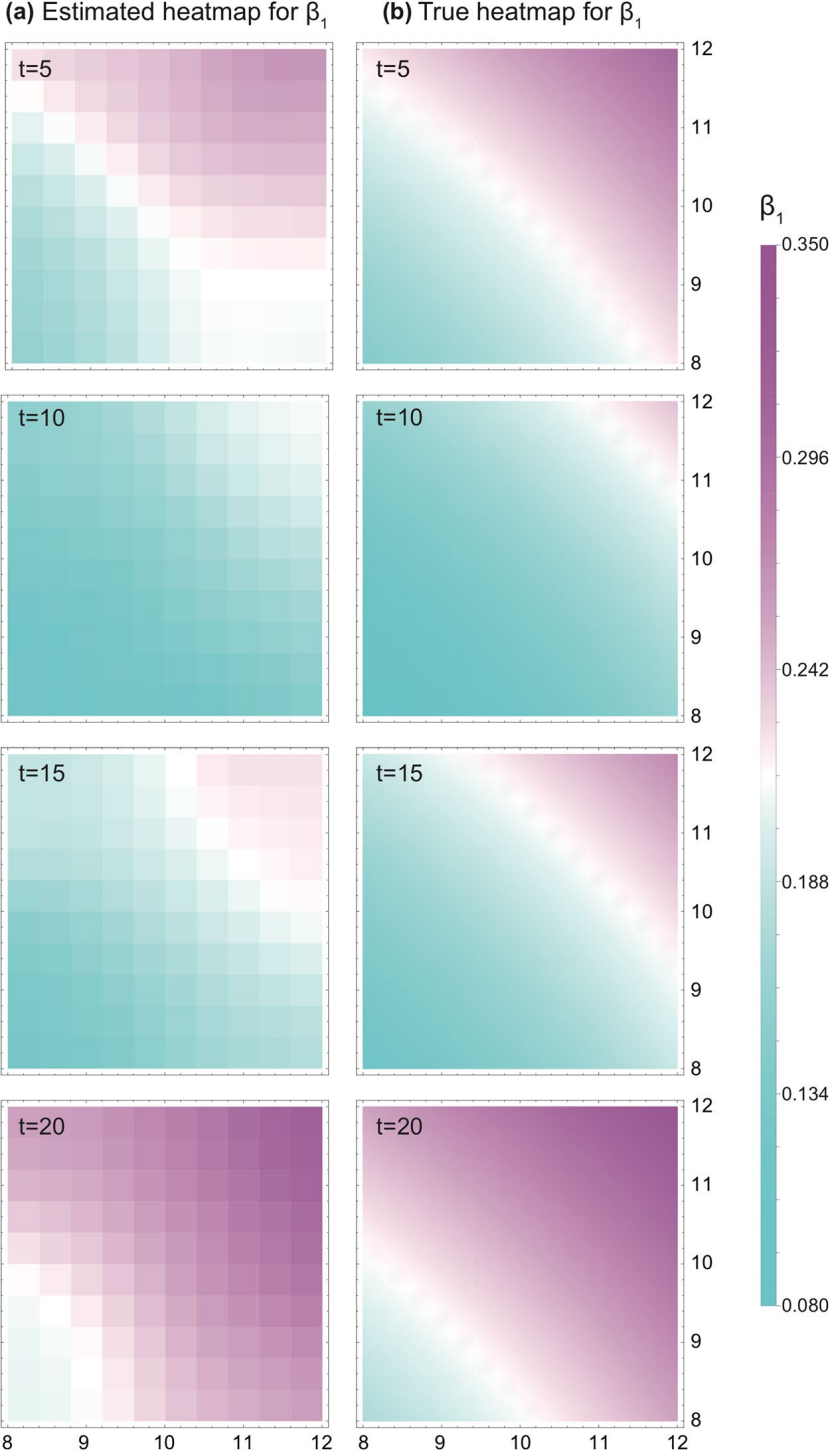
Heatmap of the ${{\rm{\beta }}}_{1}$ estimates and their true values on the plane of arbitrary spatial distance. (**a**) The estimates of ${{\rm{\beta }}}_{1}$ based on GWTCLR method in time 5, 10, 15, 20 of the simulated data set A. (**b**) Heatmap of true values of ${{\rm{\beta }}}_{1}$ in time 5, 10, 15, 20. The color bars are showing the magnitude scales for panel a and b.

**Figure 2:**
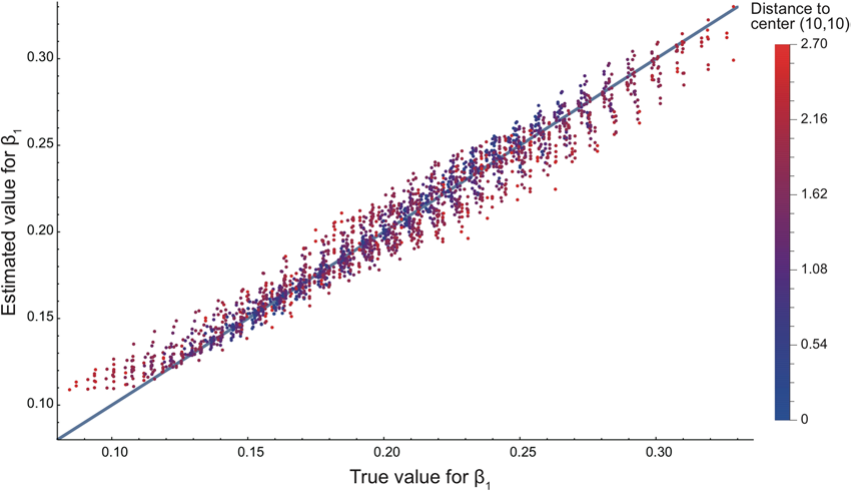
Scatter plot of estimated ${{\rm{\beta }}}_{1}$. The true value of ${{\rm{\beta }}}_{1}$ is plotted against the corresponding estimates by GWTCLR method in all time points and locations for simulated data set A. The nodes are displayed using different color based on their distance to the geographical center (10, 10).

We applied the GWTCLR estimation on the simulated data sets. Because their sample sizes (100 locations for each data set) are large, the $\tau$ of $\tau$-nearest temporal set is set 0 to avoid bias. The optimal geographical bandwidth and the kernel function are used. The heatmap of GWTCLR estimates for the coefficient ${\beta }_{1}$ as well as the heatmap of true values for coefficient ${\beta }_{1}$ of data set A are presented in Fig. 1. The two heatmaps are largely consistent with each other, yet bias is present on the geographic boundary. Figure 2 displays the scatter plot of the coefficient ${\beta }_{1}$ estimates against their true values in data set A, the Pearson correlation between estimates and true value is 0.98 (${\rm{p}} < 0.005$), indicating a high consistency. While GWTCLR estimates perform well for the locations close to geographical center (colored with blue), bias can be seen for locations on the boundary (colored with red). We further conducted a linear regression analysis of the estimated values for the coefficient ${\beta }_{1}$ and their true values of data set A, the result is given in Table 1. An approximated equality can be achieved when locations’ distance to the geographical center decrease. The bias on the boundary is due to relatively smaller sample size and unevenly distributed neighbors for location on the boundary.

**Table 1:** Linear regression analysis of estimated and true values for β_1_.

Independent variable	Estimate (95% CI)	S.E	P value
**All locations (n = 2,100)**			
intercept	0.0163 (0.0146, 0.018)	0.0009	<0.0001
true value for β_1_	0.9205 (0.9125, 0.9286)	0.0041	<0.0001
**Location’s distance to geographical center < 2 (n = 1,680)**			
intercept	0.0117 (0.0098, 0.0135)	0.0009	<0.0001
true value for β_1_	0.9443 (0.9356, 0.953)	0.0045	<0.0001
**Location’s distance to geographical center < 1**.**5 (n = 924)**			
intercept	0.0018 (−0.0004, 0.0041)	0.0011	0.106
true value for β_1_	0.9947 (0.984, 1.0055)	0.0055	<0.0001

**Figure 3:**
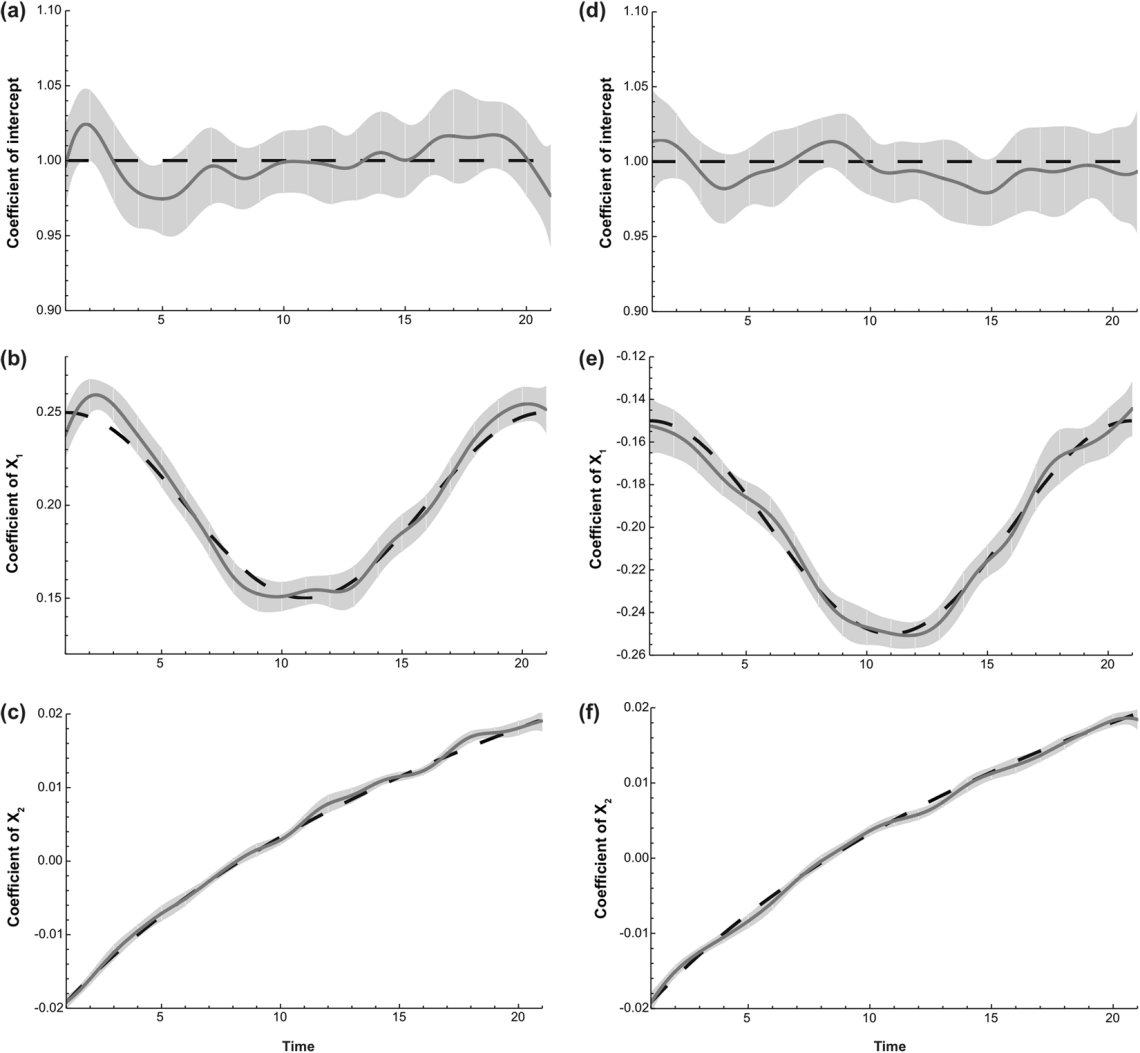
Through-time regression coefficient ($\beta$) estimates for the geographical center of data set A and B. Results for location (10, 10) and (−10, −10) are shown in the left (**a**–**c**) and right (**d**–**f**) columns respectively. The true coefficient functions are shown by dashed line and the estimates are shown by solid line. The 95% point-wise confidence intervals are shown by the shaded grey areas.

We choose the geographical center of data set A and B, whose location is (10, 10) and (−10, −10) respectively, to show the GWTCLR estimate for coefficient ${\beta }_{0},\,{\beta }_{1}$ and ${\beta }_{2}$ on the temporal domain. Figure 3 displays the true coefficients function (dashed line) and they GWTCLR estimates (solid line) together with the 95% confidence interval. The GWTCLR estimate has successfully captured the temporally varying pattern of all 3 coefficients.

## Application to Human Influenza Data

We have implemented the estimation method in Mathematica 11 code (available at http://github.com/id-bioinfo/GWTCLR) and applied to a human influenza data set. It has been previously demonstrated that global dynamics of influenza epidemics are determined by the seasonal fluctuation in climatic factors such as temperature, amount of precipitation and relative humidity, and that the relationships between these climatic factors and influenza incidence are significantly different between distantly separated locations^2^. In this section, by fitting the data to the proposed GWTCLR model, we aim to explore the spatio-temporal variations of the impacts of these climatic factors on the influenza incidence in 2012–2013. Guidelines and codes for replicating the results of this application are provided in Supplementary Codes.

### Data

Influenza surveillance data between 3 October 2011 and 30 March 2014 were downloaded from the World Health Organization via FluNet (http://www.who.int/influenza/gisrs_laboratory/flunet/en/). Weekly number of laboratory-confirmed influenza positive samples and weekly number of specimens processed are used together as binary data, where influenza-positive specimens are coded as 1 and others as 0 and are binned monthly because of the absence of weekly climate data. A total of 22 countries, either from Europe or Southeast Asia, are chosen for analysis because these countries have relatively smaller sizes as only country-level data are available, and are considered as temperate and tropical regions that are believed to play distinct roles in human influenza transmission^2,23^.

Monthly temperature (in degrees Celsius), amount of precipitation (in mm/month) and vapor pressure (in hPa) of the 22 countries were collected from CRUCY v. 3.23 Dataset^24^ between October 2011 and March 2014. Relative humidity is approximated by the ratio of actual vapor pressure and saturate vapor pressure, while saturate vapor pressure is calculated from temperature by Teten’s equation.

The countries’ geographical center coordinates were downloaded from Wolfram Mathematica and hence the geographical distance (unit: kilometer) instead of the Euclidean distance was used in the geographical weight function. We also conducted the separate analysis by replacing the geographical center coordinates with the capital center coordinates of the countries (data not shown). We found that this has little impact to the estimates and conclusion.

We have four parameters including the intercept. The $\tau$ of $\tau$-nearest temporal set is set to be 3 and we estimated a 7-month average coefficients. To ensure every month of year 2012 and year 2013 has a complete $\tau$-nearest temporal set, we also include data of October-December, 2011 and data of January-March, 2014.

### Results

We first searched the optimal spatial distance bandwidth by using all data and followed the method in Section “Geographical Bandwidth Selection”, we used the GWR 4.0 software with a fixed Gaussian kernel type and “spherical” coordinates. The optimal bandwidth at 1,450 km with an AICc = 43199.570, which presents a “valid-fit” in the outcome was chosen.

**Figure 4:**
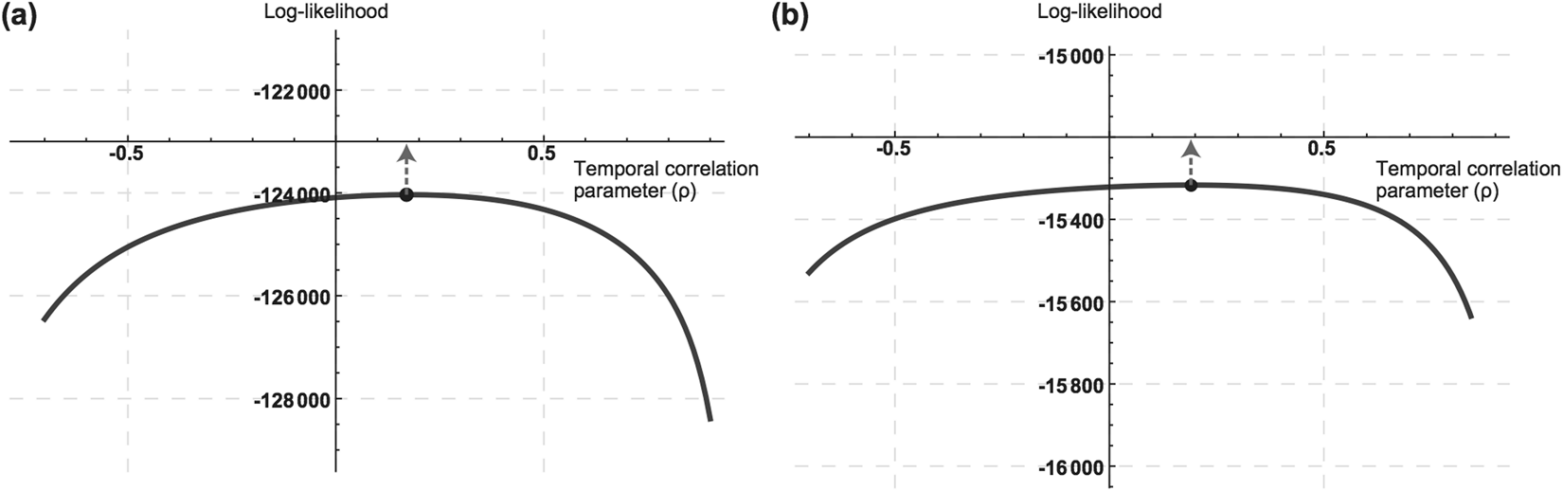
Likelihood profiles of maximum likelihood estimates of temporal correlation parameters ($\rho$) for France and Thailand. Profiles for France and Thailand are shown on panel a and b respectively. The circles indicate the maxima, with arrows pointing to the approximate maximum likelihood estimates.

We determined the temporal correlation parameter ${\rho }_{i}$ of each country. Since the sampling interval in this study is regular, we chose the AR(1) autocorrelation structure to account for potential negative correlation. We used all data and assigned a Gaussian distance decay-based function with bandwidth value 1,450 km as the geographical weight function for each country’s likelihood. We calculated the MLE of coefficients under each possible value of ${\rho }_{i}$ with a step size of 0.01, generated the log-likelihood profile, from which the approximate MLE of ${\rho }_{i}$ was identified. For instance, the optimal value of ${\rho }_{i}$ is 0.17 for France (maximum log-likelihood is −124035.5) and 0.19 for Thailand (maximum log-likelihood is −15316.55) (Fig. 4), both suggesting a positive correlation.

We applied our GWTCLR model on all data to estimate the coefficients for each of the 22 countries. Noteworthy, estimations at the time points with insufficient samples were attained by smoothing method. The bandwidth $h\,\,$and order $p$ in the kernel function were selected by plotting the raw estimates $\hat{b}$ along with refined estimates $\hat{\beta }$. For instance, $p$ was set to be 2 and *h* was 4 for France and 3 for Thailand.

**Figure 5:**
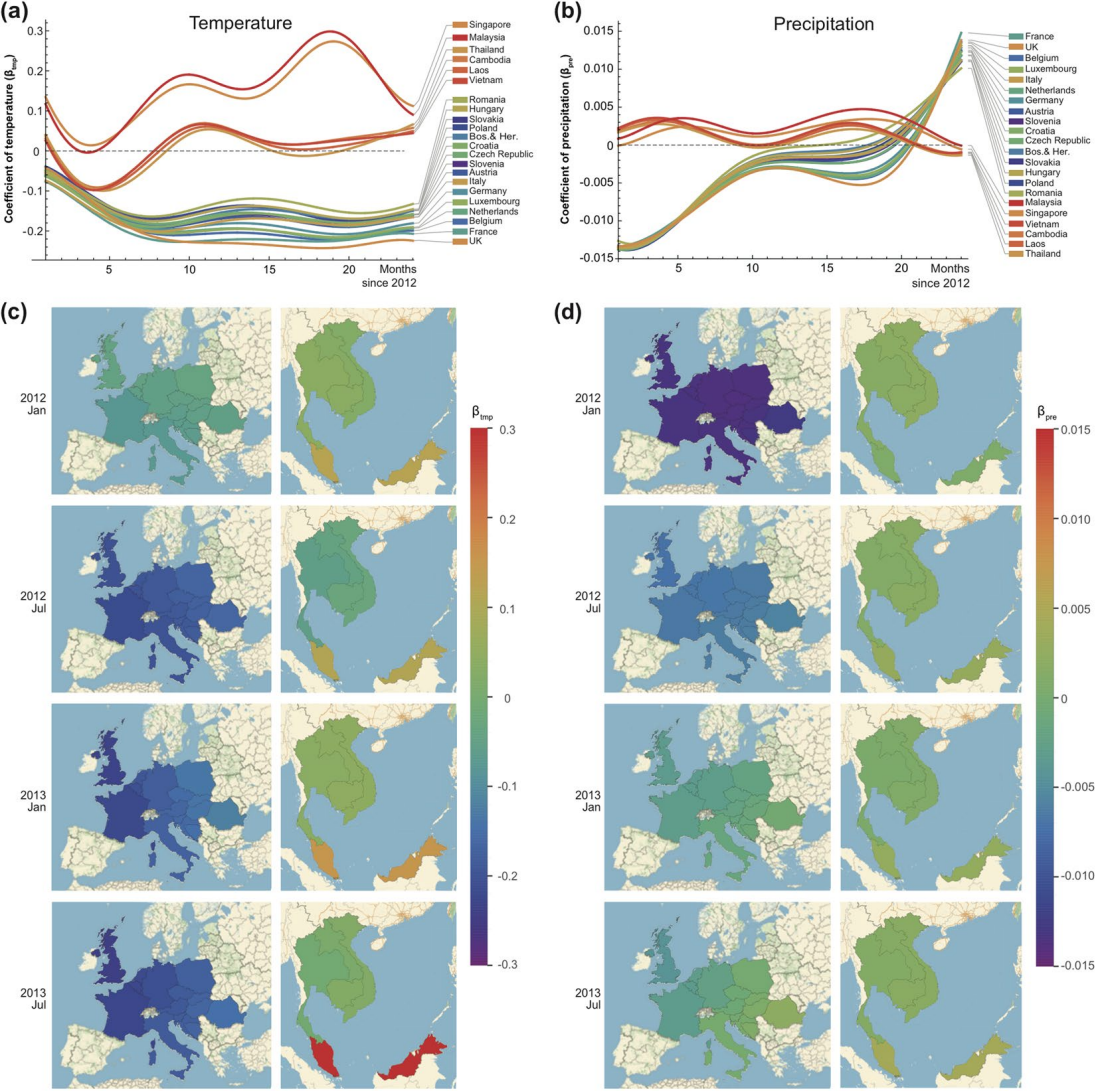
Regression coefficients (β) estimates for temperature and precipitation in Europe and Southeast Asia. Through-time regression coefficient estimates of temperature (β_tmp_; panel a) and precipitation (β_pre_; panel b) for 22 countries. Maps of the coefficients of temperature (β_tmp_) in European and Southeast Asian regions in January 2012, July 2012, January 2013 and July 2013 are shown in panel c. Maps for the precipitation coefficients (β_pre_) are shown in panel d. The color bars are showing the magnitude scales for panel c,d. The results of relative humidity are omitted because their confidence intervals are wide and cover zero, and thus indicate no significant correlation. The maps were created using Mathematica (version: 11, https://www.wolfram.com/mathematica/).

**Figure 6:**
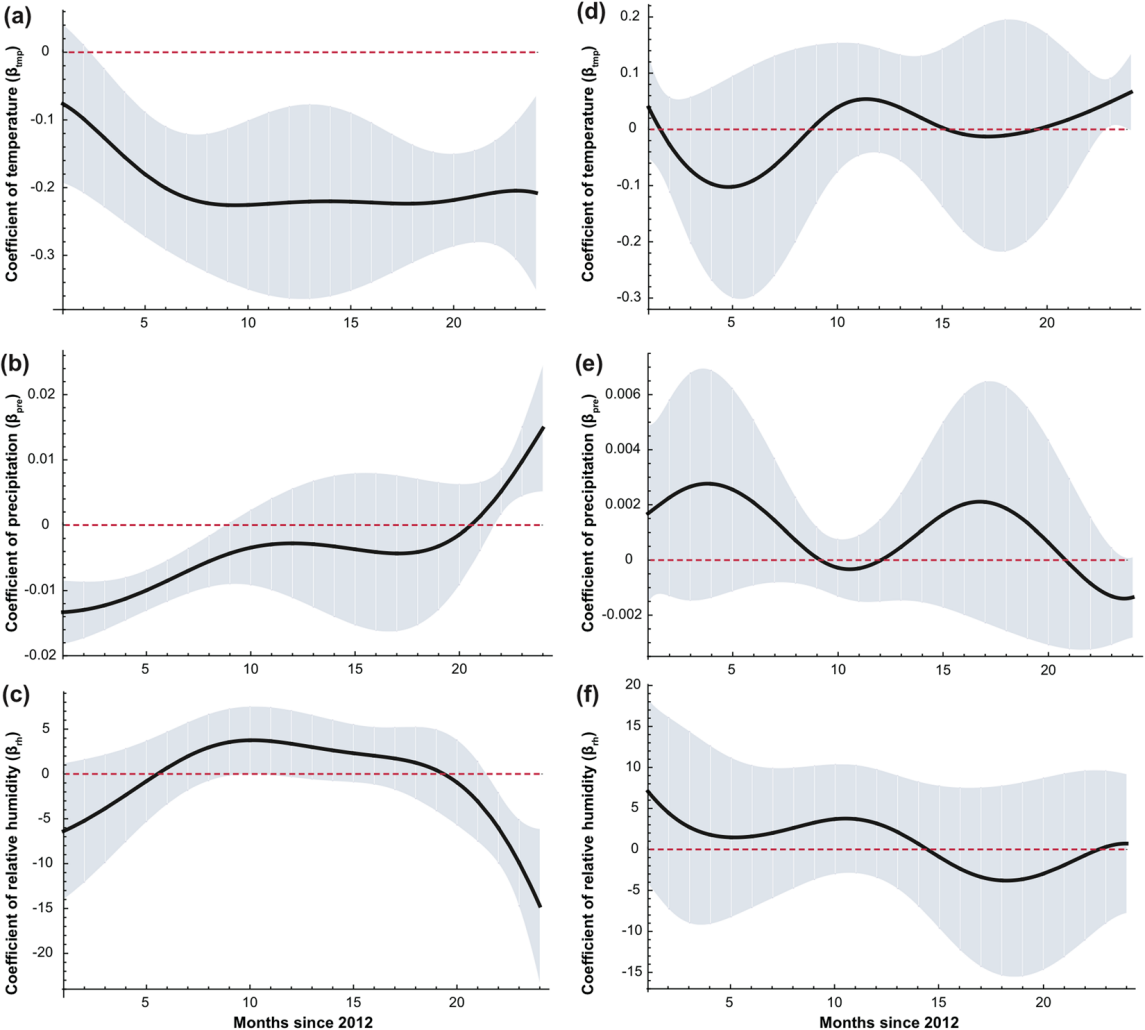
Through-time regression coefficient (β) estimates for climatic factors in France and Thailand. Climatic factors include temperature (β_tmp_; panel a,d), precipitation (${\rm{\beta }}\,$_pre_; panel b,e) and relative humidity (β_rh_; panel c,f). Results for France and Thailand are shown in the left (**a**–**c**) and right (**d**–**f**) columns respectively. The 95% point-wise confidence intervals are shown by the shaded blue areas.

The refined estimates for the coefficients of all countries between 2012 and 2013 are presented in Fig. 5. The result for France is highlighted in Fig. 6a–c for further illustration. It is observed that temperature shows a significantly negative effect on influenza incidence as the 95% confidence interval is almost completely below the zero. Considering that France locates in a high latitude region, this result is consistent with the previous studies either from laboratory evidence^25^ or epidemiologic analysis^2^. For precipitation, a rather complicated pattern is observed. A negative effect is shown from month 1 to 8 (corresponding to January 2012-August 2012, denoted as Period I), no significant difference from zero is shown from month 9 to 21 (corresponding to September 2012-September 2013, denoted as Period II) and a positive effect is shown from month 22 to 24 (corresponding to October 2013-December 2013, denoted as Period III). There are no previous studies relating to the time-varying effect of precipitation.

**Figure 7:**
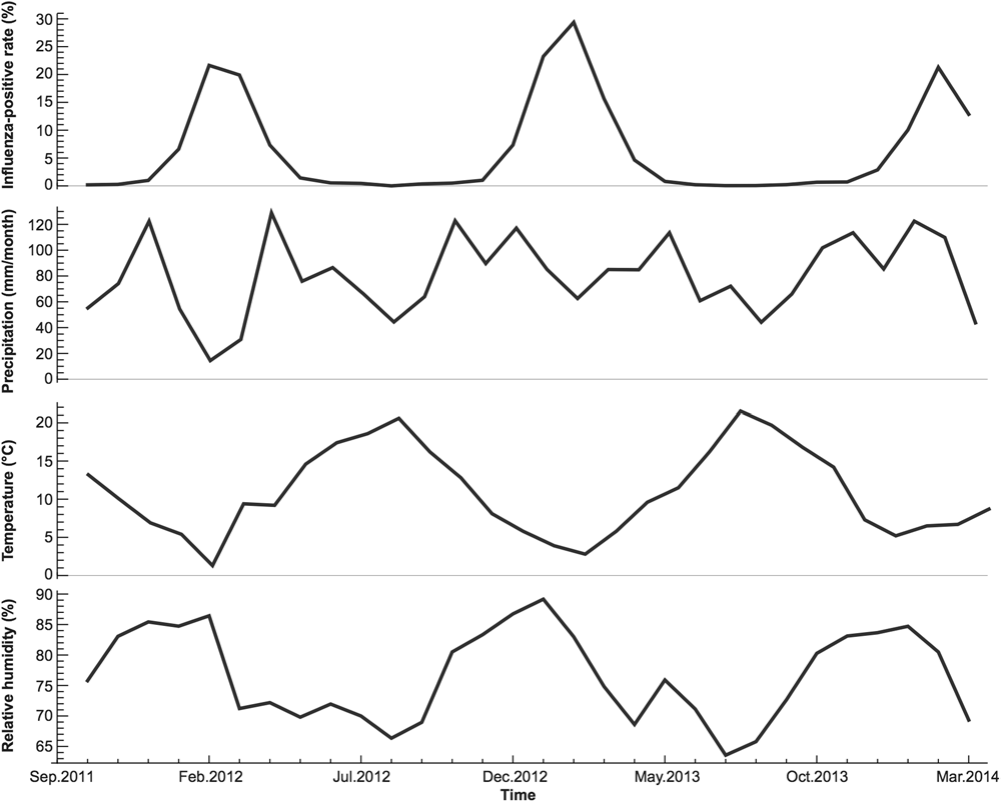
Observed influenza-positive rate, precipitation, relative humidity and temperature in France, 2012–2013.

We assessed the time-varying correlation of precipitation in France by referring to the raw data of influenza-positive samples and precipitation, as shown in Fig. 7. Three obvious crests of influenza-positive samples are observed at Dec 2011-Apr 2012, Dec 2012-Apr 2013, and Dec 2013-Mar 2014. The first crest corresponded apparently to the substantial valley of precipitation, explaining the significant negative correlation estimated by GWTCLR. The second crest coincided with a much smaller valley of precipitation, also franking by considerably low level of influenza activities but fluctuating precipitation in the summer of 2012 and 2013. This might contribute to the lack of correlation till October 2013. After Oct 2013, it is the third crest of influenza activity concurring with precipitation, which is mainly characterized by their same drops from January to February 2014. These visual results demonstrated that our model could capture the time-varying relationship between dependent and independent variables.

The effect of relative humidity was found insignificant during most period although it showed a negative effect at the end of year 2013. However, previous laboratory studies^23^ have suggested that relative humidity has negative effect on the influenza activity. Two reasons possibly explain this discordance: Firstly, the relative humidity of France between October 2011 and March 2014 did not vary largely (with a relatively small standard deviation 0.074) and it maintained at a high level (with a median 75.84%) that did not provide sufficient variability in the independent variable. Secondly, it has been pointed out that relative humidity is indeed a weaker predictor of influenza activity compared with temperature^2,25^, so the true effect of relative humidity may be masked by temperature especially when temperature varies more significantly.

The refined estimates of the coefficients in Thailand between 2012 and 2013 are presented in Fig. 6d–f. All these three factors (temperature, precipitation and relative humidity) do not show a significant effect on the influenza activity. Considering the latitude of Thailand is ${15}^{^\circ }N$, this result was consistent with findings by Tamerius *et al*.^2^ that in middle latitudes between 12.5° and 25° N/S, there was no significant association between climatic variables and influenza peaks. Moreover, the lack of association was also consistent with Deyle *et al*.^23^ which attributed to the vaguer seasonality in tropical countries.

Spatially distributed coefficients for temperature and precipitation show a clear spatial clustering (Fig. 5). For example, European countries show a similar negative association between influenza activity and temperature, which is self-evident given the regular winter peak of influenza in European countries^26^. For Southeast Asian countries, although Singapore and Malaysia deviated from others with quite high coefficients of temperature, none of the coefficient estimates are considered significant because their confidence intervals cover zero. In terms of the association between influenza activity and precipitation, European countries like France show an initial negative association, transiting to a positive association at the end of study period, which is also supported by visual inspection of the data (Fig. 7). Southeast Asian countries remain non-significant throughout 2012–2013, which is consistent with the previous report of weak causality^2^. Overall, our GWTCLR model managed to identify the spatially and temporally varying relationships between influenza disease incidence and climatic variables.

## Discussions

In this paper, we propose a geographically weighted temporally correlated logistic regression model (GWTCLR) that is designated for binary outcome data such as disease detection results from public health surveillance. This model integrates the geographically weighted logistic regression (GWLR) model^8^ and two-step estimation approach^6^, to deal with spatial and temporal non-stationarity simultaneously. We showed the asymptotic properties of the proposed estimator. We also provided a way to estimate the asymptotic covariance under some regularity conditions. Our model is implemented and applied to the regional influenza detection results published by WHO FluNet. GWTCLR obtained consistent conclusions with previous studies, while also revealed the temporal change of association between disease prevalence and climates that could not be shown in previous studies that were unable to accommodate the temporal and spatial non-stationarities simultaneously. Furthermore, it is noteworthy that, unlike the previous temporal and geographical linear regression model^11^, our GWTCLR also accounts for possible temporal correlation of the longitudinal data.

Our GWTCLR model is an extension of two commonly used models in literature^6,8^, and can be easily reduced to previous models and other variant models by a simple specification of some parameters. For example, if we assume all samples are independent, then by setting ${\rm{corr}}({Y}_{i,{t}_{1},j},{Y}_{i,{t}_{2},k})=0$, GWTCLR is reduced to GWTLR. If we further assume coefficients are temporally invariable, then by setting $\tau$ large enough so as to use all samples from the whole time period, GWTCLR is further reduced to GWLR. The same idea applies to the spatial component, by setting an extremely large bandwidth in the geographical weight function, all weights are forced to be equal, the estimation approach reduces to the two-step estimation approach.

An interesting and useful prospect of GWTCLR is that, if a large number of samples is collected from locations that sufficiently spread over a specific region, we can estimate the coefficients at any coordinates within that region and at any time point of the whole period. Therefore, a through-time animation of geographic heatmap showing the coefficient estimates at every location can be constructed. This can help users to visualize the temporally and spatially varying magnitudes and directions of the predictor’s impacts on the outcomes in an intuitive and comprehensive manner. However, drawing such heatmaps over the time requires relatively larger sampling sizes, coverages and frequencies, as well as computational time. Further research can investigate how the application of GWTCLR could be benefited by better sampling techniques and more efficient inference algorithms.

Several limitations of GWTCLR should be highlighted for future investigation. First, the asymptotic normality test could be studied further, for example, with different sample sizes. Second, we assume a weakly stationary tetrachoric correlation structure to reduce the number of temporal correlation parameters, but the temporal correlation may vary at different times, which could be accommodated by an adaptive correlation structure. Third, a spatio-temporally constant geographical bandwidth was assumed in order to simplify the model, yet this may not be appropriate as the localization of association pattern for different places is likely not constant, and such localization may also change with time. We believe that future research should address this issue, allowing the geographical bandwidth to be dynamic. Fourth, while GWTCLR is an extension of logistic regression model, similar extension may be possible for other generalized linear models.

## Electronic supplementary material

Supplementary Methods

Supplementary Codes

Supplementary Data

## References

[1] Hastie, T. & Tibshirani, R. Varying-coefficient models. *J Roy Stat Soc B*, 757–796 (1993).

[2] Tamerius, J. D. *et al*. Environmental Predictors of Seasonal Influenza Epidemics across Temperate and Tropical Climates. *Plos Pathog* **9** (2013). 10.1371/journal.ppat.1003194 PMC3591336 23505366

[3] Fan JQ, Zhang JT (2000). Two-step estimation of functional linear models with applications to longitudinal data. J Roy Stat Soc B.

[4] Cai ZW, Fan JQ, Li RZ (2000). Efficient estimation and inferences for varying-coefficient models. J Am Stat Assoc.

[5] Senturk D, Dalrymple LS, Mohammed SM, Kaysen GA, Nguyen DV (2013). Modeling time-varying effects with generalized and unsynchronized longitudinal data. Stat Med.

[6] Dong J, Estes JP, Li G, Senturk D (2016). A two-step estimation approach for logistic varying coefficient modeling of longitudinal data. J Stat Plan Infer.

[7] Brunsdon C, Fotheringham AS, Charlton ME (1996). Geographically weighted regression: A method for exploring spatial nonstationarity. Geogr Anal.

[8] Fotheringham, A. S., Brunsdon, C. & Charlton, M. *Geographically weighted regression: the analysis of spatially varying relationships*. (Wiley, 2002).

[9] Tobler WR (1970). A computer movie simulating urban growth in the Detroit region. Economic geography.

[10] Nakaya T, Fotheringham AS, Brunsdon C, Charlton M (2005). Geographically weighted Poisson regression for disease association mapping. Stat Med.

[11] Huang B, Wu B, Barry M (2010). Geographically and temporally weighted regression for modeling spatio-temporal variation in house prices. Int J Geogr Inf Sci.

[12] Wu B, Li RR, Huang B (2014). A geographically and temporally weighted autoregressive model with application to housing prices. Int J Geogr Inf Sci.

[13] Fotheringham AS, Crespo R (2015). & Yao. J. Geographical and Temporal Weighted Regression (GTWR). Geogr Anal.

[14] Hu, M. G. *et al*. Determinants of the Incidence of Hand, Foot and Mouth Disease in China Using Geographically Weighted Regression Models. *Plos One* **7** (2012). 10.1371/journal.pone.0038978 PMC3377651 22723913

[15] Lin CH, Wen TH (2011). Using Geographically Weighted Regression (GWR) to Explore Spatial Varying Relationships of Immature Mosquitoes and Human Densities with the Incidence of Dengue. Int J Env Res Pub He.

[16] Tsai, P. J. & Yeh, H. C. Scrub typhus islands in the Taiwan area and the association between scrub typhus disease and forest land use and farmer population density: geographically weighted regression. *Bmc Infect Dis* **13** (2013). 10.1186/1471-2334-13-191 PMC3648375 23627966

[17] Wu, L. *et al*. Spatial Analysis of Severe Fever with Thrombocytopenia Syndrome Virus in China Using a Geographically Weighted Logistic Regression Model. *Int J Env Res Pub He* **13** (2016). 10.3390/ijerph13111125 PMC5129335 27845737

[18] Zhou YB (2016). Geographical variations of risk factors associated with HCV infection in drug users in southwestern China. Epidemiol Infect.

[19] Lecessie S, Vanhouwelingen JC (1994). Logistic-Regression for Correlated BinaryData. Appl Stat-J Roy St C.

[20] Staniswalis JG (1989). The Kernel Estimate of a Regression Function in Likelihood-Based Models. J Am Stat Assoc.

[21] Fan, J. & Gijbels, I. *Local polynomial modelling and its applications*. 1st edn, (Chapman & Hall, 1996).

[22] Hurvich CM, Simonoff JS, Tsai CL (1998). Smoothing parameter selection in nonparametric regression using an improved Akaike information criterion. J Roy Stat Soc B.

[23] Deyle ER, Maher MC, Hernandez RD, Basu S, Sugihara G (2016). Global environmental drivers of influenza. P Natl Acad Sci USA.

[24] Harris I, Jones PD, Osborn TJ, Lister DH (2014). Updated high-resolution grids of monthly climatic observations - the CRU TS3.10 Dataset. Int J Climatol.

[25] Lowen AC, Mubareka S, Steel J, Palese P (2007). Influenza virus transmission is dependent on relative humidity and temperature. Plos Pathog.

[26] Cox NJ, Subbarao K (2000). Global epidemiology of influenza: Past and present. Annu Rev Med.

