# Supplementary material for: Geographically weighted temporally correlated logistic regression model
Source: Sci Rep. 2018 Jan 23;8:1417. doi: 10.1038/s41598-018-19772-6 (PMC5780421; doi:10.1038/s41598-018-19772-6)
Supplement: Supplementary file 1 — Supplementary Methods [file 41598_2018_19772_MOESM1_ESM.pdf]

Supplementary Methods to

Geographically weighted temporally correlated logistic regression model

Authors:

Yang Liu[1,2], Kwok-Fai Lam[3], Joseph T Wu[2], Tommy Tsan-Yuk Lam[1,2]*

Affiliations:

[1] Center of Influenza Research, State Key Laboratory of Emerging Infectious Diseases, The University of Hong Kong

[2] School of Public Health, The University of Hong Kong

[3] Department of Statistics and Actuarial Science, The University of Hong Kong

* Corresponding author
Email: ttylam@hku.hk
Postal address: 5/F, Laboratory Block, Li Ka Shing Faculty of Medicine, The University of Hong Kong, 21 Sassoon Road, Pokfulam, Hong Kong SAR, China.

Tetrachoric Correlation

The tetrachoric correlation approach, proposed by Lecessie and Vanhouwelingen[1], is adopted to accommodate the potential association among the observations. The tetrachoric correlation will replace the original definition of correlation to describe the temporal correlation of the binary variables $Y_{i,t,j}$ defined in the main text.

Suppose there is a random pair of binary variables $Y = (Y_1, Y_2)$ of which Y is a realization of a bivariate continuous random variable $Z = (Z_1, Z_2)$, so that the correlation of Y_1 and Y_2 can be represented by that of Z. Suppose Z follows a standard bivariate normal distribution with correlation ρ, then we call ρ as the tetrachoric correlation of Y. To be more specific, let

$$\begin{aligned} &\mathrm{P}(Y_1 = 1) = \pi_1, \mathrm{P}(Y_2 = 1) = \pi_2, \\ &g_1 = \Phi^{-1}(\pi_1), g_2 = \Phi^{-1}(\pi_2), \end{aligned} \tag{1}$$

where Φ is the cumulative distribution function of a standard normal distribution.

Suppose that

$$\begin{aligned} &\mathrm{P}(Y_1 = 1, Y_2 = 1) = \pi_{11}; \\ &\mathrm{P}(Y_1 = 1, Y_2 = 0) = \pi_{10}; \\ &\mathrm{P}(Y_1 = 0, Y_2 = 1) = \pi_{01}; \\ &\mathrm{P}(Y_1 = 0, Y_2 = 0) = \pi_{00}, \end{aligned} \tag{2}$$

and let $\varphi_m(z, u, \Sigma)$ be the probability density function of bivariate normal distribution with dimension m, mean u and covariance matrix Σ. Then we have

$$\begin{aligned} \pi_{11} &= P(Z_1 < g_1, Z_2 < g_2) = \int_{-\infty}^{g_1}\int_{-\infty}^{g_2} \varphi_2\left((z_1, z_2), 0, \begin{pmatrix} 1 & \rho \\ \rho & 1 \end{pmatrix}\right) dz_1 dz_2; \\ \pi_{10} &= P(Z_1 < g_1, Z_2 > g_2) = \int_{-\infty}^{g_1}\int_{g_2}^{+\infty} \varphi_2\left((z_1, z_2), 0, \begin{pmatrix} 1 & \rho \\ \rho & 1 \end{pmatrix}\right) dz_1 dz_2; \\ \pi_{01} &= P(Z_1 > g_1, Z_2 < g_2) = \int_{g_1}^{+\infty}\int_{-\infty}^{g_2} \varphi_2\left((z_1, z_2), 0, \begin{pmatrix} 1 & \rho \\ \rho & 1 \end{pmatrix}\right) dz_1 dz_2; \\ \pi_{00} &= P(Z_1 > g_1, Z_2 > g_2) = \int_{g_1}^{+\infty}\int_{g_2}^{+\infty} \varphi_2\left((z_1, z_2), 0, \begin{pmatrix} 1 & \rho \\ \rho & 1 \end{pmatrix}\right) dz_1 dz_2. \end{aligned} \tag{3}$$

Pseudo-Likelihood

In Section "Model Construction", the spatio-temporal local log-likelihood is rather complicated and difficult to differentiate. Here we use the pseudo-likelihood introduced in literature[1] to approximate the true likelihood.

Remember in the joint event $Y_{i,t_k} = (Y_1.Y_2, \ldots\ldots, Y_{N_{i,t_k}})$ defined in the main text, we have N_{i,t_k} marginal events. We will now divide the joint event to a set of $C_{N_{i,t_k}}^2$ pairwise events. Similar to Le

Cessie and van Houwelingen[1], define $l_{i,t_k,(p,q)}$ as the pairwise log likelihood for the pairwise event $(Y_p.Y_q)$ with sampling time (t_p,t_q) and independent covariate vector $(X_p.X_q)$. Then, let the pseudo likelihood that simplifies l_{i,t_k} be

$$l_{i,t_k}^{pse}=\frac{1}{N_{i,t_k}-1}\sum_{q=2}^{N_{i,t_k}}\sum_{p=1}^{q-1} l_{i,t_k,(p,q)}, \tag{4}$$

where

$$l_{i,t_k,(p,q)}=\sum_{a=0}^{1}\sum_{b=0}^{1} 1_{\{Y_p=a,Y_q=b\}}\log\left(\pi_{ab,(pq)}\right), \tag{5}$$

such that

$$\begin{aligned}
\pi_{11,(pq)}&=\int_{-\infty}^{g_p}\int_{-\infty}^{g_q}\varphi_2\left(z_1,z_2,0,\begin{pmatrix}1 & c_i\left(\left|t_p-t_q\right|\right)\\ c_i\left(\left|t_p-t_q\right|\right) & 1\end{pmatrix}\right)dz_1dz_2;\\
\pi_{10,(pq)}&=\int_{-\infty}^{g_p}\int_{g_q}^{+\infty}\varphi_2\left(z_1,z_2,0,\begin{pmatrix}1 & c_i\left(\left|t_p-t_q\right|\right)\\ c_i\left(\left|t_p-t_q\right|\right) & 1\end{pmatrix}\right)dz_1dz_2;\\
\pi_{01,(pq)}&=\int_{g_p}^{+\infty}\int_{-\infty}^{g_q}\varphi_2\left(z_1,z_2,0,\begin{pmatrix}1 & c_i\left(\left|t_p-t_q\right|\right)\\ c_i\left(\left|t_p-t_q\right|\right) & 1\end{pmatrix}\right)dz_1dz_2;\\
\pi_{00,(pq)}&=\int_{g_p}^{+\infty}\int_{g_q}^{+\infty}\varphi_2\left(z_1,z_2,0,\begin{pmatrix}1 & c_i\left(\left|t_p-t_q\right|\right)\\ c_i\left(\left|t_p-t_q\right|\right) & 1\end{pmatrix}\right)dz_1dz_2,
\end{aligned} \tag{6}$$

with $g_p=\Phi^{-1}\left(\frac{\exp\left(X_p\beta\right)}{1+\exp\left(X_p\beta\right)}\right)$ and $g_q=\Phi^{-1}\left(\frac{\exp\left(X_q\beta\right)}{1+\exp\left(X_q\beta\right)}\right)$. Then, the pseudo temporal local log likelihood function for location *i* and time t_k is given by

$$\bar{l}_{i,t_k}^{pse}=\sum_{j=1}^{M} W_{ij} l_{j,t_k}^{pse}, \tag{7}$$

and the maximum pseudo local likelihood estimate for $\beta(u_i,v_i,t_k)$ is given by

$$\hat{b}\left(u_i,v_i,t_k\right)=\underset{\beta}{\operatorname{argmax}}\,\bar{l}_{i,,t_k}^{pse}. \tag{8}$$

In order to get the estimate, we need to differentiate $\bar{l}_{i,t_k}^{pse}$ respect to β. This is equivalent to obtain the derivative of each $l_{i,t_k,(p,q)}$. We have

$$\frac{\partial l_{i,t_k,(p,q)}}{\partial\beta}=\sum_{a=0}^{1}\sum_{b=0}^{1}\frac{1_{\{Y_p=a,Y_q=b\}}}{\pi_{ab,(pq)}}\frac{\partial\pi_{ab,(pq)}}{\partial\beta}. \tag{9}$$

Taking expectation of the second order derivative, we have

$$\mathrm{E}\left\{\frac{\partial^2 l_{i,t_k,(p,q)}}{\partial\beta^2}\right\}=-\left\{\sum_{a=0}^{1}\sum_{b=0}^{1}\frac{1}{\pi_{ab,(pq)}}\left(\frac{\partial\pi_{ab,(pq)}}{\partial\beta}\right)\left(\frac{\partial\pi_{ab,(pq)}}{\partial\beta}\right)'\right\}, \tag{10}$$

with

$$\frac{\partial \pi_{11,(pq)}}{\partial \beta} = \Phi\left(\frac{g_q - g_p c_i\left(\left|t_p - t_q\right|\right)}{\sqrt{1 - c_i\left(\left|t_p - t_q\right|\right)^2}}\right)\frac{\partial\left(\frac{\exp\left(X_p\beta\right)}{1+\exp\left(X_p\beta\right)}\right)}{\partial \beta} + \Phi\left(\frac{g_p - g_q c_i\left(\left|t_p - t_q\right|\right)}{\sqrt{1 - c_i\left(\left|t_p - t_q\right|\right)^2}}\right)\frac{\partial\left(\frac{\exp\left(X_q\beta\right)}{1+\exp\left(X_q\beta\right)}\right)}{\partial \beta};$$

$$\frac{\partial \pi_{10,(pq)}}{\partial \beta} = \Phi\left(\frac{-g_q + g_p c_i\left(\left|t_p - t_q\right|\right)}{\sqrt{1 - c_i\left(\left|t_p - t_q\right|\right)^2}}\right)\frac{\partial\left(\frac{\exp\left(X_p\beta\right)}{1+\exp\left(X_p\beta\right)}\right)}{\partial \beta} - \Phi\left(\frac{g_p - g_q c_i\left(\left|t_p - t_q\right|\right)}{\sqrt{1 - c_i\left(\left|t_p - t_q\right|\right)^2}}\right)\frac{\partial\left(\frac{\exp\left(X_q\beta\right)}{1+\exp\left(X_q\beta\right)}\right)}{\partial \beta};$$

$$\frac{\partial \pi_{01,(pq)}}{\partial \beta} = -\Phi\left(\frac{g_q - g_p c_i\left(\left|t_p - t_q\right|\right)}{\sqrt{1 - c_i\left(\left|t_p - t_q\right|\right)^2}}\right)\frac{\partial\left(\frac{\exp\left(X_p\beta\right)}{1+\exp\left(X_p\beta\right)}\right)}{\partial \beta} + \Phi\left(\frac{-g_p + g_q c_i\left(\left|t_p - t_q\right|\right)}{\sqrt{1 - c_i\left(\left|t_p - t_q\right|\right)^2}}\right)\frac{\partial\left(\frac{\exp\left(X_q\beta\right)}{1+\exp\left(X_q\beta\right)}\right)}{\partial \beta};$$

$$\frac{\partial \pi_{00,(pq)}}{\partial \beta} = -\Phi\left(\frac{-g_q + g_p c_i\left(\left|t_p - t_q\right|\right)}{\sqrt{1 - c_i\left(\left|t_p - t_q\right|\right)^2}}\right)\frac{\partial\left(\frac{\exp\left(X_p\beta\right)}{1+\exp\left(X_p\beta\right)}\right)}{\partial \beta} - \Phi\left(\frac{-g_p + g_q c_i\left(\left|t_p - t_q\right|\right)}{\sqrt{1 - c_i\left(\left|t_p - t_q\right|\right)^2}}\right)\frac{\partial\left(\frac{\exp\left(X_q\beta\right)}{1+\exp\left(X_q\beta\right)}\right)}{\partial \beta}.$$

We can attain the MLE using Fisher Scoring method based on the iteration function

$$\beta^{(k+1)} = \beta^{(k)} + \mathrm{I}\left(\beta^{(k)}\right)^{-1}\left[\frac{\partial \bar{l}_{i,t_k}^{pse}}{\partial \beta}\left(\beta^{(k)}\right)\right], \tag{11}$$

the iteration stops when the solution converges and we obtain the raw estimates $\hat{b}(u_i, v_i, t_k)$.

Proof of Theorem 1

We write $l_M(\beta)$ and $\overline{l_M}(\beta)$ as following

$$l_M(\beta) = \bar{l}_{i,t_k}(\beta) = \sum_{j=1}^{M} W_{ij}\log\left(f_{j,t_k}\left(Y_{j,t_k}|\beta, X_{j,t_k}\right)\right),$$

$$\overline{l_M}(\beta) = E_0(\bar{l}_{i,t_k}(\beta)) = \sum_{j=1}^{M} E_0(W_{ij}\log\left(f_{j,t_k}\left(Y_{j,t_k}|\beta, X_{j,t_k}\right)\right)),$$

since $\beta(u_i, v_i, t_k)$ is the true parameter, $\forall\beta \in B$

$$
\begin{aligned}
&\overline{l_M}(\beta)-\overline{l_M}(\beta(u_i,v_i,t_k))\\
&=\sum_{j=1}^{M}W_{ij}E_0\left(\log\left(f_{j,t_k}\left(Y_{j,t_k}|\beta,X_{j,t_k}\right)\right)-\log\left(f_{j,t_k}\left(Y_{j,t_k}|\beta(u_i,v_i,t_k),X_{j,t_k}\right)\right)\right)\\
&=\sum_{j=1}^{M}W_{ij}E_0\left(\log\left(\frac{f_{j,t_k}\left(Y_{j,t_k}|\beta,X_{j,t_k}\right)}{f_{j,t_k}\left(Y_{j,t_k}|\beta(u_i,v_i,t_k),X_{j,t_k}\right)}\right)\right)\\
&=\sum_{j=1}^{M}W_{ij}\sum_{Y_{j,t_k}}\log\left(\frac{f_{j,t_k}\left(Y_{j,t_k}|\beta,X_{j,t_k}\right)}{f_{j,t_k}\left(Y_{j,t_k}|\beta(u_i,v_i,t_k),X_{j,t_k}\right)}\right)f_{j,t_k}\left(Y_{j,t_k}|\beta(u_j,v_j,t_k),X_{j,t_k}\right)\\
&\le\sum_{j=1}^{M}W_{ij}\sum_{Y_{j,t_k}}\left(\frac{f_{j,t_k}\left(Y_{j,t_k}|\beta,X_{j,t_k}\right)}{f_{j,t_k}\left(Y_{j,t_k}|\beta(u_i,v_i,t_k),X_{j,t_k}\right)}-1\right)f_{j,t_k}\left(Y_{j,t_k}|\beta(u_j,v_j,t_k),X_{j,t_k}\right)\\
&=\sum_{j=1}^{M}W_{ij}\sum_{Y_{j,t_k}}\left(f_{j,t_k}\left(Y_{j,t_k}|\beta,X_{j,t_k}\right)\frac{f_{j,t_k}\left(Y_{j,t_k}|\beta(u_j,v_j,t_k),X_{j,t_k}\right)}{f_{j,t_k}\left(Y_{j,t_k}|\beta(u_i,v_i,t_k),X_{j,t_k}\right)}-f_{j,t_k}\left(Y_{j,t_k}|\beta(u_j,v_j,t_k),X_{j,t_k}\right)\right).
\end{aligned}
$$

When the bandwidth of the geographical weight function is small enough, then for those $\beta(u_j,v_j,t_k)$ which is significantly different to $\beta(u_i,v_i,t_k)$ (e.g. when the geographical distance between location j and i is long enough) will have a weight function W_{ij} close to 0. Also, since $\beta(u_i,v_i,t_k)$ is smooth related to u_i,v_i, for those neighbor locations where $\beta(u_j,v_j,t_k)$ are not significantly different to $\beta(u_i,v_i,t_k)$, and in practice the trivial difference can be ignored. We simply set $\beta(u_j,v_j,t_k)=\beta(u_i,v_i,t_k)$, for those neighbor location under small bandwidth. Then we have

$$\frac{f_{j,t_k}\left(Y_{j,t_k}|\beta(u_j,v_j,t_k),X_{j,t_k}\right)}{f_{j,t_k}\left(Y_{j,t_k}|\beta(u_i,v_i,t_k),X_{j,t_k}\right)}=1$$

$$
\begin{aligned}
&\overline{l_M}(\beta)-\overline{l_M}(\beta(u_i,v_i,t_k))\\
&\le\sum_{j=1}^{M}W_{ij}1_{\{W_{ij}>0\}}\sum_{Y_{j,t_k}}\left(f_{j,t_k}\left(Y_{j,t_k}|\beta,X_{j,t_k}\right)-f_{j,t_k}\left(Y_{j,t_k}|\beta(u_j,v_j,t_k),X_{j,t_k}\right)\right)=0.
\end{aligned}
$$

So when bandwidth is sufficiently small, we have

$$\overline{l_M}(\beta(u_i,v_i,t_k))\ge\overline{l_M}(\beta),\forall M>0,\beta\in B.$$

Now consider for an open neighborhood

$$B_r(\beta(u_i,v_i,t_k))=\{\beta|\beta-\beta(u_i,v_i,t_k)<r\}\subset B,\forall r,$$

under assumption 1 and by Kolmogorov strong law of large numbers, we have

$$P\left(\lim_{M\to+\infty}\frac{1}{M}(l_M(\beta)-\overline{l_M}(\beta))=0\right)=1,\forall\beta\in B.$$

In other words, for any $\beta\in B_r(\beta(u_i,v_i,t_k))$, we have

$$\frac{1}{M}(l_M(\beta)-l_M(\beta(u_i,v_i,t_k)))\overset{a.s.}{\to}\lim_{M\to\infty}\frac{1}{M}\left(\overline{l_M}(\beta)-\overline{l_M}(\beta(u_i,v_i,t_k))\right)\le 0,$$

because $l_M(\beta)$ is continuous with respect to β in $B_r(\beta(u_i,v_i,t_k))$. Therefore when M is sufficiently large, it must have a local maximum point, denoted by $\hat{b}(u_i,v_i,t_k)$. Since $l_M(\beta)$ is

differentiable, hence when M is sufficiently large, we must have

$$\frac{dl_M(\beta)}{d\beta}|_{\beta=\hat{b}(u_i,v_i,t_k)}=0.$$

Now because of arbitrary value of r and $\| \hat{b}(u_i,v_i,t_k)-\beta(u_i,v_i,t_k) \| < r$, we have

$$\hat{b}(u_i,v_i,t_k)\xrightarrow{P}\beta(u_i,v_i,t_k) \text{ when } M\to+\infty \quad \square$$

Proof of Theorem 2

We explicitly write the vector form $\beta=(\beta_1,\beta_2,\ldots,\beta_p)^T$,because $\hat{b}(u_i,v_i,t_k)$ is the MLE of $\bar{l}_{i,t_k}(\beta)$, so by the mean value theorem, for every $j\in\{1,2,\ldots\ldots,p\}$

$$0=\frac{\partial\bar{l}_{i,t_k}(\beta)}{\partial\beta_j}|_{\beta=\hat{b}(u_i,v_i,t_k)}=\frac{\partial\bar{l}_{i,t_k}(\beta)}{\partial\beta_j}|_{\beta=\beta(u_i,v_i,t_k)}+\nabla\frac{\partial\bar{l}_{i,t_k}(\beta)}{\partial\beta_j}|_{\beta=\beta^*}\left(\hat{b}(u_i,v_i,t_k)-\beta(u_i,v_i,t_k)\right).$$

Then write the above equation in vector form, we have

$$0=\frac{\partial\bar{l}_{i,t_k}(\beta)}{\partial\beta}|_{\beta=\hat{b}(u_i,v_i,t_k)}=\frac{\partial\bar{l}_{i,t_k}(\beta)}{\partial\beta}|_{\beta=\beta(u_i,v_i,t_k)}+\frac{\partial^2\bar{l}_{i,t_k}(\beta^*)}{\partial\beta^2}\left(\hat{b}(u_i,v_i,t_k)-\beta(u_i,v_i,t_k)\right),$$

where

$$\frac{\partial^2\bar{l}_{i,t_k}(\beta^*)}{\partial\beta^2}=\begin{pmatrix} \frac{\partial^2\bar{l}_{i,t_k}(\beta)}{\partial\beta_1^2}|_{\beta=\beta_1^*} & \frac{\partial^2\bar{l}_{i,t_k}(\beta)}{\partial\beta_1\partial\beta_2}|_{\beta=\beta_1^*} & \cdots & \frac{\partial^2\bar{l}_{i,t_k}(\beta)}{\partial\beta_1\partial\beta_p}|_{\beta=\beta_1^*} \\ \frac{\partial^2\bar{l}_{i,t_k}(\beta)}{\partial\beta_2\partial\beta_1}|_{\beta=\beta_2^*} & \frac{\partial^2\bar{l}_{i,t_k}(\beta)}{\partial\beta_2^2}|_{\beta=\beta_2^*} & \cdots & \frac{\partial^2\bar{l}_{i,t_k}(\beta)}{\partial\beta_2\partial\beta_p}|_{\beta=\beta_2^*} \\ \vdots & \vdots & \ddots & \vdots \\ \frac{\partial^2\bar{l}_{i,t_k}(\beta)}{\partial\beta_p\partial\beta_1}|_{\beta=\beta_p^*} & \frac{\partial^2\bar{l}_{i,t_k}(\beta)}{\partial\beta_p\partial\beta_2}|_{\beta=\beta_p^*} & \cdots & \frac{\partial^2\bar{l}_{i,t_k}(\beta)}{\partial\beta_p^2}|_{\beta=\beta_p^*} \end{pmatrix},$$

and

$$\begin{cases} \beta_1^*=c_1\hat{b}(u_i,v_i,t_k)+(1-c_1)\beta(u_i,v_i,t_k), \\ \beta_2^*=c_2\hat{b}(u_i,v_i,t_k)+(1-c_2)\beta(u_i,v_i,t_k), \\ \vdots \\ \beta_p^*=c_p\hat{b}(u_i,v_i,t_k)+(1-c_p)\beta(u_i,v_i,t_k), \end{cases} 0\le c_1,\ldots,c_p\le 1.$$

So we have

$$\hat{b}(u_i,v_i,t_k)-\beta(u_i,v_i,t_k)=-\left(\frac{\partial^2\bar{l}_{i,t_k}(\beta^*)}{\partial\beta^2}\right)^{-1}\left(\frac{\partial\bar{l}_{i,t_k}(\beta)}{\partial\beta}|_{\beta=\beta(u_i,v_i,t_k)}\right).$$

Consider $\frac{\partial\bar{l}_{i,t_k}(\beta)}{\partial\beta}|_{\beta=\beta(u_i,v_i,t_k)}$, under assumption 2 *ii*, *iii*, and by the multivariate Lindeberg-Feller Central Limit Theorem[2], we have

$$\sqrt{M}\left(\frac{1}{M}\frac{\partial\bar{l}_{i,t_k}(\beta)}{\partial\beta}|_{\beta=\beta(u_i,v_i,t_k)}-\frac{1}{M}E_0\left(\frac{\partial\bar{l}_{i,t_k}(\beta)}{\partial\beta}|_{\beta=\beta(u_i,v_i,t_k)}\right)\right)\xrightarrow{d}N(0,\Sigma)$$

or equivalently,

$$\sqrt{M}\frac{1}{M}\frac{\partial\bar{l}_{i,t_k}(\beta)}{\partial\beta}|_{\beta=\beta(u_i,v_i,t_k)}\sim AN\left(\frac{1}{\sqrt{M}}E_0\left(\frac{\partial\bar{l}_{i,t_k}(\beta)}{\partial\beta}|_{\beta=\beta(u_i,v_i,t_k)}\right),\overline{\Sigma_M}\right).$$

Now we have:

$$\begin{aligned}
&E_0\left(\frac{\partial\bar{l}_{i,t_k}(\beta)}{\partial\beta}|_{\beta=\beta(u_i,v_i,t_k)}\right)\\
&=E_0\left(\sum_{j=1}^{M}W_{ij}\frac{\frac{\partial f_{j,t_k}(Y_{j,t_k}|\beta,X_{j,t_k})}{\partial\beta}|_{\beta=\beta(u_i,v_i,t_k)}}{f_{j,t_k}(Y_{j,t_k}|\beta(u_i,v_i,t_k),X_{j,t_k})}\right)\\
&=\sum_{j=1}^{M}W_{ij}\sum_{Y_{j,t_k}}\left(\left(\frac{\partial f_{j,t_k}(Y_{j,t_k}|\beta,X_{j,t_k})}{\partial\beta}|_{\beta=\beta(u_i,v_i,t_k)}\right)\frac{f_{j,t_k}(Y_{j,t_k}|\beta(u_j,v_j,t_k),X_{j,t_k})}{f_{j,t_k}(Y_{j,t_k}|\beta(u_i,v_i,t_k),X_{j,t_k})}\right)
\end{aligned}$$

As in the proof of Theorem 1, under the condition with large sample size and small bandwidth, $W_{ij}=0$ for a distant location *j*. While for a neighbor location j, by the smoothing property of $\beta(u,v,t)$, we have

$$\frac{f_{j,t_k}(Y_{j,t_k}|\beta(u_j,v_j,t_k))}{f_{j,t_k}(Y_{j,t_k}|\beta(u_i,v_i,t_k))}=1,$$

then

$$E_0\left(\frac{\partial\bar{l}_{i,t_k}(\beta)}{\partial\beta}|_{\beta=\beta(u_i,v_i,t_k)}\right)=\sum_{j=1}^{M}W_{ij}1_{\{W_{ij}>0\}}\sum_{Y_{j,t_k}}\left(\frac{\partial f_{j,t_k}(Y_{j,t_k}|\beta,X_{j,t_k})}{\partial\beta}|_{\beta=\beta(u_i,v_i,t_k)}\right),$$

$$\because\sum_{Y_{j,t_k}}f_{j,t_k}(Y_{j,t_k}|\beta,X_{j,t_k})=1,\forall\beta\in B,$$

therefore

$$E_0\left(\frac{\partial\bar{l}_{i,t_k}(\beta)}{\partial\beta}|_{\beta=\beta(u_i,v_i,t_k)}\right)=0,$$

and hence

$$\sqrt{M}\frac{1}{M}\frac{\partial\bar{l}_{i,t_k}(\beta)}{\partial\beta}|_{\beta=\beta(u_i,v_i,t_k)}\xrightarrow{d}N(0,\Sigma).$$

Now, consider $\frac{\partial^2 \bar{l}_{i,t_k}(\beta^*)}{\partial \beta^2}$, with assumption 2.*iv* and by Kolmogorov strong law of large numbers, we have

$$\frac{1}{M}\left(\frac{\partial^2 \bar{l}_{i,t_k}(\beta)}{\partial \beta^2}|_{\beta=\beta(u_i,v_i,t_k)}\right)\xrightarrow{P} I.$$

By Theorem 1, we know that $\hat{b}(u_i,v_i,t_k)\xrightarrow{P}\beta(u_i,v_i,t_k)$, and because β_n^* lies between $\hat{b}(u_i,v_i,t_k)$ and $\beta(u_i,v_i,t_k)$ for $1 \le n \le$ p, then we have

$$\frac{1}{M}\frac{\partial^2 \bar{l}_{i,t_k}(\beta^*)}{\partial \beta^2}\xrightarrow{P} I.$$

As we have already attained the asymptotic property of $\frac{\partial \bar{l}_{i,t_k}(\beta)}{\partial \beta}|_{\beta=\beta(u_i,v_i,t_k)}$ and $\frac{\partial^2 \bar{l}_{i,t_k}(\beta^*)}{\partial \beta^2}$, then by Slutsky's theorem, we have

$$\sqrt{M}\left(\hat{b}(u_i,v_i,t_k)-\beta(u_i,v_i,t_k)\right)\xrightarrow{d} N\left(0, I^{-1}\Sigma I^{-1}\right),$$

or equivalently, when sample size is large enough, we have

$$\sqrt{M}\left(\hat{b}(u_i,v_i,t_k)-\beta(u_i,v_i,t_k)\right)\sim AN\left(0, M{I_M}^{-1}\Sigma_M {I_M}^{-1}\right). \qquad \square$$

References:

1 Lecessie, S. & Vanhouwelingen, J. C. Logistic-Regression for Correlated Binary Data. *Appl Stat-J Roy St C* **43**, 95-108 (1994).

2 Greene, W. H. *Econometric analysis*. 5th edn, (Prentice Hall, 2003).
