# Supplementary material for: Geographically weighted temporally correlated logistic regression model
Source: Sci Rep. 2018 Jan 23;8:1417. doi: 10.1038/s41598-018-19772-6 (PMC5780421; doi:10.1038/s41598-018-19772-6)

Supplementary Codes to

Geographically weighted temporally correlated logistic regression model

Authors:

Yang Liu[1,2], Kwok-Fai Lam[3], Joseph T Wu[2], Tommy Tsan-Yuk Lam[1,2]*

Affiliations:

[1] Center of Influenza Research, State Key Laboratory of Emerging Infectious Diseases, The University of Hong Kong

[2] School of Public Health, The University of Hong Kong

[3] Department of Statistics and Actuarial Science, The University of Hong Kong

* Corresponding author

Email: ttylam@hku.hk

Postal address: 5/F, Laboratory Block, Li Ka Shing Faculty of Medicine, The University of Hong Kong, 21 Sassoon Road, Pokfulam, Hong Kong SAR, China.

Please first run the following basic code

```
prob[x_, beta_] := Exp[x.beta] / (1 + Exp[x.beta]);

(*rou[t1_,t2_,d_]:=Exp[−Abs[t1−t2]*Exp[d]];*)
rou[t1_, t2_, d_] := d^(Abs[t1 − t2]);

TNear[t_, tao_, DATA_] := Pick[DATA, Table[Abs[DATA[[i]][[5]] − t] ≤ tao, {i, 1, Length[DATA]}]];

GeoW[x_, y_, band_] := N[Exp[−((QuantityMagnitude[GeoDistance[x, y[[3 ;; 4]]]]) ^ 2) / (band ^ 2)]];
(*GeoW[x_,y_,band_]:=N[If[EuclideanDistance[x,y[[3;;4]]]<band,(1−SquaredEuclideanDistance[x[[3;;4]],y[[3;;4]]]/(band^2))^2,0]];*)

Ker[x_] := (1 / Sqrt[2 * Pi]) * Exp[−(x ^ 2) / 2];

op11[x_, beta_] := CDF[BinormalDistribution[0],
    {Quantile[NormalDistribution[0, 1], prob[x[[6 ;; Length[x]]], beta]], Quantile[NormalDistribution[0, 1], prob[x[[6 ;; Length[x]]], beta]]}];
op10[x_, beta_] := CDF[BinormalDistribution[0], {Quantile[NormalDistribution[0, 1], prob[x[[6 ;; Length[x]]], beta]],
      −Quantile[NormalDistribution[0, 1], prob[x[[6 ;; Length[x]]], beta]]}];
op00[x_, beta_] := CDF[BinormalDistribution[0], {−Quantile[NormalDistribution[0, 1], prob[x[[6 ;; Length[x]]], beta]],
      −Quantile[NormalDistribution[0, 1], prob[x[[6 ;; Length[x]]], beta]]}];

p11[x_, y_, beta_, d_] := CDF[BinormalDistribution[rou[x[[5]], y[[5]], d]],
    {Quantile[NormalDistribution[0, 1], prob[x[[6 ;; Length[x]]], beta]], Quantile[NormalDistribution[0, 1], prob[y[[6 ;; Length[x]]], beta]]}];
p10[x_, y_, beta_, d_] := CDF[NormalDistribution[0, 1], Quantile[NormalDistribution[0, 1], prob[x[[6 ;; Length[x]]], beta]]] − p11[x, y, beta, d];
p01[x_, y_, beta_, d_] := CDF[NormalDistribution[0, 1], Quantile[NormalDistribution[0, 1], prob[y[[6 ;; Length[x]]], beta]]] − p11[x, y, beta, d];
p00[x_, y_, beta_, d_] := 1 − p11[x, y, beta, d] − p10[x, y, beta, d] − p01[x, y, beta, d];

odp11[x_, beta_] := CDF[NormalDistribution[0, 1], Quantile[NormalDistribution[0, 1], prob[x[[6 ;; Length[x]]], beta]]] *
      (Exp[x[[6 ;; Length[x]]].beta] / (1 + Exp[x[[6 ;; Length[x]]].beta]) ^ 2) * x[[6 ;; Length[x]]] +
    CDF[NormalDistribution[0, 1], Quantile[NormalDistribution[0, 1], prob[x[[6 ;; Length[x]]], beta]]] *
      (Exp[x[[6 ;; Length[x]]].beta] / (1 + Exp[x[[6 ;; Length[x]]].beta]) ^ 2) * x[[6 ;; Length[x]]];
odp10[x_, beta_] := CDF[NormalDistribution[0, 1], −Quantile[NormalDistribution[0, 1], prob[x[[6 ;; Length[x]]], beta]]] *
      (Exp[x[[6 ;; Length[x]]].beta] / (1 + Exp[x[[6 ;; Length[x]]].beta]) ^ 2) * x[[6 ;; Length[x]]] −
    CDF[NormalDistribution[0, 1], Quantile[NormalDistribution[0, 1], prob[x[[6 ;; Length[x]]], beta]]] *
      (Exp[x[[6 ;; Length[x]]].beta] / (1 + Exp[x[[6 ;; Length[x]]].beta]) ^ 2) * x[[6 ;; Length[x]]];
odp00[x_, beta_] := −CDF[NormalDistribution[0, 1], −Quantile[NormalDistribution[0, 1], prob[x[[6 ;; Length[x]]], beta]]] *
      (Exp[x[[6 ;; Length[x]]].beta] / (1 + Exp[x[[6 ;; Length[x]]].beta]) ^ 2) * x[[6 ;; Length[x]]] −
    CDF[NormalDistribution[0, 1], −Quantile[NormalDistribution[0, 1], prob[x[[6 ;; Length[x]]], beta]]] *
      (Exp[x[[6 ;; Length[x]]].beta] / (1 + Exp[x[[6 ;; Length[x]]].beta]) ^ 2) * x[[6 ;; Length[x]]];
```

```
dp11[x_, y_, beta_, d_] :=
    CDF[NormalDistribution[0, 1], (Quantile[NormalDistribution[0, 1], prob[y[[6 ;; Length[x]]], beta]] − Quantile[NormalDistribution[0, 1],
                prob[x[[6 ;; Length[x]]], beta]] * rou[x[[5]], y[[5]], d]) / (Sqrt[1 − (rou[x[[5]], y[[5]], d]) ^ 2])] *
        (Exp[x[[6 ;; Length[x]]].beta] / (1 + Exp[x[[6 ;; Length[x]]].beta]) ^ 2) * x[[6 ;; Length[x]]] +
      CDF[NormalDistribution[0, 1], (Quantile[NormalDistribution[0, 1], prob[x[[6 ;; Length[x]]], beta]] −
            Quantile[NormalDistribution[0, 1], prob[y[[6 ;; Length[x]]], beta]] * rou[x[[5]], y[[5]], d]) /
          (Sqrt[1 − (rou[x[[5]], y[[5]], d]) ^ 2])] * (Exp[y[[6 ;; Length[x]]].beta] / (1 + Exp[y[[6 ;; Length[x]]].beta]) ^ 2) * y[[6 ;; Length[x]]];
dp10[x_, y_, beta_, d_] := CDF[NormalDistribution[0, 1], (−Quantile[NormalDistribution[0, 1], prob[y[[6 ;; Length[x]]], beta]] +
            Quantile[NormalDistribution[0, 1], prob[x[[6 ;; Length[x]]], beta]] * rou[x[[5]], y[[5]], d]) /
          (Sqrt[1 − (rou[x[[5]], y[[5]], d]) ^ 2])] * (Exp[x[[6 ;; Length[x]]].beta] / (1 + Exp[x[[6 ;; Length[x]]].beta]) ^ 2) * x[[6 ;; Length[x]]] −
      CDF[NormalDistribution[0, 1], (Quantile[NormalDistribution[0, 1], prob[x[[6 ;; Length[x]]], beta]] −
            Quantile[NormalDistribution[0, 1], prob[y[[6 ;; Length[x]]], beta]] * rou[x[[5]], y[[5]], d]) /
          (Sqrt[1 − (rou[x[[5]], y[[5]], d]) ^ 2])] * (Exp[y[[6 ;; Length[x]]].beta] / (1 + Exp[y[[6 ;; Length[x]]].beta]) ^ 2) * y[[6 ;; Length[x]]];
dp01[x_, y_, beta_, d_] := −CDF[NormalDistribution[0, 1], (Quantile[NormalDistribution[0, 1], prob[y[[6 ;; Length[x]]], beta]] −
            Quantile[NormalDistribution[0, 1], prob[x[[6 ;; Length[x]]], beta]] * rou[x[[5]], y[[5]], d]) /
          (Sqrt[1 − (rou[x[[5]], y[[5]], d]) ^ 2])] * (Exp[x[[6 ;; Length[x]]].beta] / (1 + Exp[x[[6 ;; Length[x]]].beta]) ^ 2) * x[[6 ;; Length[x]]] +
      CDF[NormalDistribution[0, 1], (−Quantile[NormalDistribution[0, 1], prob[x[[6 ;; Length[x]]], beta]] +
            Quantile[NormalDistribution[0, 1], prob[y[[6 ;; Length[x]]], beta]] * rou[x[[5]], y[[5]], d]) /
          (Sqrt[1 − (rou[x[[5]], y[[5]], d]) ^ 2])] * (Exp[y[[6 ;; Length[x]]].beta] / (1 + Exp[y[[6 ;; Length[x]]].beta]) ^ 2) * y[[6 ;; Length[x]]];
dp00[x_, y_, beta_, d_] := −CDF[NormalDistribution[0, 1], (−Quantile[NormalDistribution[0, 1], prob[y[[6 ;; Length[x]]], beta]] +
            Quantile[NormalDistribution[0, 1], prob[x[[6 ;; Length[x]]], beta]] * rou[x[[5]], y[[5]], d]) /
          (Sqrt[1 − (rou[x[[5]], y[[5]], d]) ^ 2])] * (Exp[x[[6 ;; Length[x]]].beta] / (1 + Exp[x[[6 ;; Length[x]]].beta]) ^ 2) * x[[6 ;; Length[x]]] −
      CDF[NormalDistribution[0, 1], (−Quantile[NormalDistribution[0, 1], prob[x[[6 ;; Length[x]]], beta]] +
            Quantile[NormalDistribution[0, 1], prob[y[[6 ;; Length[x]]], beta]] * rou[x[[5]], y[[5]], d]) /
          (Sqrt[1 − (rou[x[[5]], y[[5]], d]) ^ 2])] * (Exp[y[[6 ;; Length[x]]].beta] / (1 + Exp[y[[6 ;; Length[x]]].beta]) ^ 2) * y[[6 ;; Length[x]]];
```

```
I1[x_, beta_] := Binomial[x[[1]], 2] * (1 / op11[x, beta]) * odp11[x, beta] +
      x[[1]] * (x[[2]] − x[[1]]) * (1 / op10[x, beta]) * odp10[x, beta] + Binomial[x[[2]] − x[[1]], 2] * (1 / op00[x, beta]) * odp00[x, beta];
```

```
O1[x_, y_, beta_, d_] := x[[1]] * y[[1]] * (1 / p11[x, y, beta, d]) * dp11[x, y, beta, d] +
      x[[1]] * (y[[2]] − y[[1]]) * (1 / p10[x, y, beta, d]) * dp10[x, y, beta, d] + y[[1]] * (x[[2]] − x[[1]]) * (1 / p01[x, y, beta, d]) * dp01[x, y, beta, d] +
      (x[[2]] − x[[1]]) * (y[[2]] − y[[1]]) * (1 / p00[x, y, beta, d]) * dp00[x, y, beta, d];
```

```
Od2p[x_, y_, beta_, d_] := −x[[2]] * y[[2]] * ((Outer[Times, dp11[x, y, beta, d], dp11[x, y, beta, d]] * (1 / p11[x, y, beta, d])) +
          (Outer[Times, dp10[x, y, beta, d], dp10[x, y, beta, d]] * (1 / p10[x, y, beta, d])) +
          (Outer[Times, dp01[x, y, beta, d], dp01[x, y, beta, d]] * (1 / p01[x, y, beta, d])) +
          (Outer[Times, dp00[x, y, beta, d], dp00[x, y, beta, d]] * (1 / p00[x, y, beta, d])));
```

```
Id2p[x_, beta_] := −Binomial[x[[2]], 2] *
      (Outer[Times, odp11[x, beta], odp11[x, beta]] * (1 / op11[x, beta]) + 2 * Outer[Times, odp10[x, beta], odp10[x, beta]] * (1 / op10[x, beta]) +
          Outer[Times, odp00[x, beta], odp00[x, beta]] * (1 / op00[x, beta]));
```

```
GTDE1[beta_, d_, DATA_] := (DR = 0;
    Do[DR += I1[DATA[[i]], beta], {i, 1, Length[DATA]}];
    Do[DR += O1[DATA[[i]], DATA[[j]], beta, d], {i, 1, Length[DATA] − 1}, {j, i + 1, Length[DATA]}]; DR);

DE1[a_, b_, band_, beta_, d_, DATA_] := (D1R = 0;
    D1Set = Split[DATA, #1[[3 ;; 4]] == #2[[3 ;; 4]] &];
    Do[
      D1R += GeoW[{a, b}, D1Set[[i]][[1]], band] * (1 / (Total[D1Set[[i]][[All, 2]]] − 1)) * GTDE1[beta, d, D1Set[[i]]], {i, 1, Length[D1Set]}];
    D1R);



GTDE2[beta_, d_, DATA_] := (FSI = 0;
    Do[FSI += Id2p[DATA[[i]], beta], {i, 1, Length[DATA]}];
    Do[FSI += Od2p[DATA[[i]], DATA[[j]], beta, d], {i, 1, Length[DATA] − 1}, {j, i + 1, Length[DATA]}]; FSI);

DE2[a_, b_, band_, beta_, d_, DATA_] := (D2R = 0;
    D1Set = Split[DATA, #1[[3 ;; 4]] == #2[[3 ;; 4]] &];
    Do[
      D2R += GeoW[{a, b}, D1Set[[i]][[1]], band] * (1 / (Total[D1Set[[i]][[All, 2]]] − 1)) * GTDE2[beta, d, D1Set[[i]]], {i, 1, Length[D1Set]}];
    D2R);



InLik[x_, beta_] :=
  Binomial[x[[1]], 2] * Log[op11[x, beta]] + x[[1]] * (x[[2]] − x[[1]]) * Log[op10[x, beta]] + Binomial[x[[2]] − x[[1]], 2] * Log[op00[x, beta]];
OutLik[x_, y_, beta_, d_] := x[[1]] * y[[1]] * Log[p11[x, y, beta, d]] + x[[1]] * (y[[2]] − y[[1]]) * Log[p10[x, y, beta, d]] +
    y[[1]] * (x[[2]] − x[[1]]) * Log[p01[x, y, beta, d]] + (x[[2]] − x[[1]]) * (y[[2]] − y[[1]]) * Log[p00[x, y, beta, d]];
GTLik[beta_, d_, DATA_] := (1 / (Total[DATA[[All, 2]]] − 1)) * (L = 0;
      Do[L += InLik[DATA[[i]], beta], {i, 1, Length[DATA]}];
      Do[L += OutLik[DATA[[i]], DATA[[j]], beta, d], {i, 1, Length[DATA] − 1}, {j, i + 1, Length[DATA]}]; L);
Lik[a_, b_, band_, beta_, d_, DATA_] := (Lik1 = 0;
    D1Set = Split[DATA, #1[[3 ;; 4]] == #2[[3 ;; 4]] &];
    Do[Lik1 += GeoW[{a, b}, D1Set[[i]][[1]], band] * GTLik[beta, d, D1Set[[i]]], {i, 1, Length[D1Set]}]; Lik1);



FinCom[a_, d_, u_, v_, band_, DATA_] := (beta = a;
  Do[beta = N[beta − Inverse[N[DE2[u, v, band, beta, d, DATA]]].N[DE1[u, v, band, beta, d, DATA]]];
    Print[beta];
    Print[N[Lik[u, v, band, beta, d, DATA]]], {i, 7}])
BetaFinCom[a_, d_, u_, v_, band_, DATA_] := (beta = a;
  Do[beta = N[beta − Inverse[N[DE2[u, v, band, beta, d, DATA]]].N[DE1[u, v, band, beta, d, DATA]]], {i, 7}]; beta)
```

```
LikFinCom[d_, u_, v_, band_, DATA_] := (beta = Table[0, {n, Length[DATA[[1]]] − 5}];
    Do[beta = N[beta − Inverse[N[DE2[u, v, band, beta, d, DATA]]].N[DE1[u, v, band, beta, d, DATA]]], {i, 7}];
    N[Lik[u, v, band, beta, d, DATA]])
```

```
PlotLik[a_, b_, n_, u_, v_, band_, DATA_] := (A = Table[0, {i, a, b, n}];
    Do[A[[Round[((i − a) / n) + 1]]] = LikFinCom[i, u, v, band, DATA], {i, a, b, n}];
    ListLinePlot[Transpose[Join[{Table[i, {i, a, b, n}]}, {A}]], AxesLabel → {Autocorrelation Parameter, Log − Likelihood}, ImageSize → Large])
```

```
ResAP[a_, b_, n_, u_, v_, band_, DATA_] := (ArP = Table[0, {i, a, b, n}];
      Pamd = Table[0, {i, a, b, n}];
      Do[ArP[[Round[((i − a) / n) + 1]]] = LikFinCom[i, u, v, band, DATA], {i, a, b, n}];
      Do[If[ArP[[j]] == Max[ArP], Pamd[[j]] = (j − 1) ∗ n + a, Pamd[[j]] = 0], {j, 1, Length[ArP]}];
      Total[Pamd]);
```

```
BetaM[Ini_, a_, b_, u_, v_, band_, DATA_] := Manipulate[BetaFinCom[Ini, d, u, v, band, DATA], {d, a, b}]
```

```
DEV[u_, v_, band_, beta_, d_, DATA_] := (D1V = 0;
      D1Set = Split[DATA, ♯1[[3 ;; 4]] == ♯2[[3 ;; 4]] &];
      Do[D1V += (GeoW[{u, v}, D1Set[[i]][[1]], band]^2) ∗ Outer[Times, (1 / (Total[D1Set[[i]][[All, 2]]] − 1)) ∗ GTDE1[beta, d, D1Set[[i]]],
              (1 / (Total[D1Set[[i]][[All, 2]]] − 1)) ∗ GTDE1[beta, d, D1Set[[i]]]], {i, 1, Length[D1Set]}];
      D1V);
RawVar[u_, v_, band_, d_, DATA_] :=
    Inverse[N[DE2[u, v, band, BetaFinCom[Table[0, {n, Length[DATA[[1]]] − 5}], d, u, v, band, DATA], d, DATA]]].
      DEV[u, v, band, BetaFinCom[Table[0, {n, Length[DATA[[1]]] − 5}], d, u, v, band, DATA], d, DATA].
      Inverse[N[DE2[u, v, band, BetaFinCom[Table[0, {n, Length[DATA[[1]]] − 5}], d, u, v, band, DATA], d, DATA]]];
```

```
RawTe[u_, v_, band_, d_, DATA_] := (B = Table[0, {n, Length[DATA[[1]]] − 5}];
      beta = BetaFinCom[Table[0, {n, Length[DATA[[1]]] − 5}], d, u, v, band, DATA];
      betaVar = RawVar[u, v, band, d, DATA];
      Do[
        B[[i]] = N[beta[[i]]] − 1.96 ∗ N[Sqrt[betaVar[[i]][[i]]]] < 0 < N[beta[[i]]] + 1.96 ∗ N[Sqrt[betaVar[[i]][[i]]]], {i, 1, Length[DATA[[1]]] − 5}];
      B);
```

```
PaDEV[u_, v_, band_, betaa_, betab_, d_, DATAa_, DATAb_] := (PaD1V = 0;
      D1Set = Split[DATAa, ♯1[[3 ;; 4]] == ♯2[[3 ;; 4]] &];
      D2Set = Split[DATAb, ♯1[[3 ;; 4]] == ♯2[[3 ;; 4]] &];
      Do[If[D1Set[[i]][[1]][[3 ;; 4]] == D2Set[[j]][[1]][[3 ;; 4]],
          PaD1V += (GeoW[{u, v}, D1Set[[i]][[1]], band]^2) ∗ Outer[Times, (1 / (Total[D1Set[[i]][[All, 2]]] − 1)) ∗ GTDE1[betaa, d, D1Set[[i]]],
                (1 / (Total[D2Set[[j]][[All, 2]]] − 1)) ∗ GTDE1[betab, d, D2Set[[j]]]],
          PaD1V += 0], {i, 1, Length[D1Set]}, {j, 1, Length[D2Set]}];
      PaD1V);
```

```
PaRawCv[u_, v_, band_, betaa_, betab_, d_, DATAa_, DATAb_] := Inverse[N[DE2[u, v, band, betaa, d, DATAa]]].
      PaDEV[u, v, band, betaa, betab, d, DATAa, DATAb].Inverse[N[DE2[u, v, band, betab, d, DATAb]]];



RawEs[Tim_, tao_, d_, u_, v_, band_, DATA_] := (betaa = Table[0, {j, Length[DATA[[1]]] - 5}];
      betaH = Table[betaa, {j, Length[Tim]}];
      ste = 1; Do[betaH[[ste]] = BetaFinCom[betaa, d, u, v, band, TNear[t, tao, DATA]]; ste += 1, {t, Tim}]; betaH);

RawCv[Tim_, tao_, d_, u_, v_, band_, betaS_, DATA_] := Table[PaRawCv[u, v, band, betaS[[i]],
        betaS[[j]], d, TNear[Tim[[i]], tao, DATA], TNear[Tim[[j]], tao, DATA]], {i, Length[Tim]}, {j, Length[Tim]}];



RefineEs[t_, p_, h_, Tim_, betaS_] := (DesM = Table[Table[0, {j, 0, p}], {i, Length[Tim]}];
      Do[DesM[[n]] = Table[Tim[[n]]^m, {m, 0, p}], {n, Length[Tim]}];
      Omig = DiagonalMatrix[Table[Ker[(t - l) / h], {l, Tim}]] ;
      Table[t^j, {j, 0, p}].Inverse[Transpose[DesM].Omig.DesM].Transpose[DesM].Omig.betaS);



RefineVar[t_, p_, h_, Tim_, Cv_] := (A = 0;
    DesM = Table[Table[0, {j, 0, p}], {i, Length[Tim]}];
    Do[DesM[[n]] = Table[Tim[[n]]^m, {m, 0, p}], {n, Length[Tim]}];
    Omig = DiagonalMatrix[Table[Ker[(t - l) / h], {l, Tim}]] ;
    Do[A += (Table[t^j, {j, 0, p}].Inverse[Transpose[DesM].Omig.DesM].Transpose[DesM].Omig[[i]]) * (Table[t^j, {j, 0, p}].
              Inverse[Transpose[DesM].Omig.DesM].Transpose[DesM].Omig[[j]]) * Cv[[i, j]], {i, 1, Length[Tim]}, {j, 1, Length[Tim]}];
    A)

PlotRefine[a_, b_, n_, p_, h_, Tim_, betaS_, Cv_] := Plot[{RefineEs[x, p, h, Tim, betaS][[n]] - 1.96 * Sqrt[RefineVar[x, p, h, Tim, Cv][[n, n]]],
        RefineEs[x, p, h, Tim, betaS][[n]], RefineEs[x, p, h, Tim, betaS][[n]] + 1.96 * Sqrt[RefineVar[x, p, h, Tim, Cv][[n, n]]]},
      {x, a, b}, Filling → {1 → {3}}, AxesLabel → {Time, Beta}, ImageSize → Large];
```

Now we import our data, please point to your own address

```
DA = Import["(*please specify the address here*).xlsx"][[1]];
DA = Drop[DA, 1]; (*drop the head*)
```

As specified in the paper, the temporal correlation parameter is determined by exhaustive search, this can be done by function “LikFinCom”, “LikFinCom” can give us the value of the log-likelihood given any location and the temporal correlation parameter. In the following example, we are going to calculate the log-likelihood of France with coordinates (46,2) when the temporal correlation parameter is -0.4. Note that the correlation structure adopted here is AR(1) correlation structure

```
LikFinCom[-0.4(*the pre-defined temporal correlation parameter*), 46(*latitude of France*),
  2(*longitude of France*), 1450(*geographical bandwidth*), DA(*our data set*)]
```

```
-124 698.
```

In our paper, we search the correlation parameter in -1 to 1 with a step-size of 0.01, the largest log-likelihood of France and Thailand are presented as follows

```
LikFinCom[0.17(*the parameter which gives the largest log−likelihood when searching step−size is 0.01*),
  46(*latitude of France*), 2(*longitude of France*), 1450, DA]
```

−124 036.

```
LikFinCom[0.19(*the parameter which gives the largest log−likelihood when searching step−size is 0.01*),
  15(*latitude of Thailand*), 100(*longitude of Thailand*), 1450, DA]
```

−15 316.6

As specified in the paper, the geographical bandwidth is 1450 and the temporal correlation parameter is 0.17 for France and 0.19 for Thailand. We can now compute the raw estimator and its log-likelihood value by using function “FinCom”. See the following example, we will calculate the raw estimator for France in Jan-2012 (time index 25):

```
FinCom[{0, 0, 0, 0}(*specify the initial value for intercept, temperature, precipitation and RH*), 0.17
  (*temporal correlation parameter*), 46(*latitude*), 2(*longitude*), 1450(*geographical bandwidth*),
  TNear[25(*specify the time index*), 3(*specify the τ of the τ−nearest temporal set*), DA(*our data set*)]]
```

{1.74831, −0.064805, −0.00432249, −3.03936}

−33 695.

{3.67617, −0.104106, −0.00901672, −5.24608}

−32 030.1

{4.34401, −0.112787, −0.0120615, −5.94532}

−31 877.3

{4.40476, −0.113085, −0.0126516, −6.00112}

−31 874.3

{4.40551, −0.113084, −0.0126666, −6.00159}

−31 874.3

{4.40551, −0.113085, −0.0126666, −6.00159}

−31 874.3

{4.40551, −0.113085, −0.0126666, −6.00159}

−31 874.3

Now we show the example of France as what we present in the paper

We need first compute all raw estimators by using function “RawEs”. Note that time index 25 relates to Jan-2012 and time index 48 relates to Dec-2013, the geographical center of France has coordinates (46,2), we skip time index 33 because of insufficient samples, the loss would be offset by smoothing the raw estimators, the result is stored in “Es”

```
Es = RawEs[{25, 26, 27, 28, 29, 30, 31, 32, 34, 35, 36, 37, 38, 39, 40, 41, 42, 43, 44, 45, 46, 47, 48}
    (*specify the time index set, here we would attain raw estimators from Jan−2012 to Dec−2013 except Sep−2012*),
    3(*specify the τ of the τ−nearest temporal set*), 0.17(*temporal correlation parameter*),
    46(*latitude*), 2(*longitude*), 1450(*geographical bandwidth*), DA(*our data set*)];
```

We now compute the corresponding covariance of the raw estimator by using function “RawCv”, the result is stored in “Cv”

```
Cv = RawCv[{25, 26, 27, 28, 29, 30, 31, 32, 34, 35, 36, 37, 38, 39, 40, 41, 42, 43, 44, 45, 46, 47, 48}(*specify the time index set,
    here we would attain the covariance of raw estimators from Jan−2012 to Dec−2013 except Sep−2012*), 3
    (*specify the τ of the τ−nearest temporal set*), 0.17(*temporal correlation parameter*), 46(*latitude*), 2
    (*longitude*), 1450(*geographical bandwidth*), Es(*the value of raw estimaors*), DA(*our data set*)];
```

We can refine the raw estimator by using function “RefineEs”, which is the GWTCLR estimator. In the following example, we are going to compute the refined estimator in Jan-2012 for France, note that we re-define the time index set (1 relates to Jan-2012 and 24 relates to Dec-2013).

```
RefineEs[1.0(*specify the time index of refined estimator, here we specify Jan−2012,
  note that you can imput any real number between 1 and 24*), 2(*order of the smoothing kernel*), 4
  (*bandwidth of the smoothing kernel*), {1, 2, 3, 4, 5, 6, 7, 8, 9, 11, 12, 13, 14, 15, 16, 17, 18, 19, 20, 21, 22, 23, 24}
  (*specify the time index set*), Es(*the value of raw estimaors*)]
```

{4.53588, −0.076548, −0.0133913, −6.51661}

We can also refine the covariance of the raw estimator by using function "RefineVar". In the following example, we are going to compute the refined covariance in Jan-2012 for France, note that we re-define the time index set (1 relates to Jan-2012 and 24 relates to Dec-2013).

```
RefineVar[1.0(*specify the time index of refined estimator, here we specify Jan−2012,
  note that you can imput any real number between 1 and 24*), 2(*order of the smoothing kernel*), 4
  (*bandwidth of the smoothing kernel*), {1, 2, 3, 4, 5, 6, 7, 8, 9, 11, 12, 13, 14, 15, 16, 17, 18, 19, 20, 21, 22, 23, 24}
  (*specify the time index set*), Cv(*the value of original covariance*)]
```

{{10.1674, −0.187401, −0.00334813, −12.3211}, {−0.187401, 0.00357939, 0.0000458471, 0.226804},
{−0.00334813, 0.0000458471, 6.19366×10^{-6}, 0.00412306}, {−12.3211, 0.226804, 0.00412306, 14.9486}}

Now we can draw the graph to see how does the coefficient change from Jan-2012 to Dec-2013

```
(*For coefficient of temperature*)
Plot[{RefineEs[x, 2, 4, {1, 2, 3, 4, 5, 6, 7, 8, 10, 11, 12, 13, 14, 15, 16, 17, 18, 19, 20, 21, 22, 23, 24}, Es][[
      2(*temperature is the second element of the estimator*)]] -
    1.96 * Sqrt[RefineVar[x, 2, 4, {1, 2, 3, 4, 5, 6, 7, 8, 10, 11, 12, 13, 14, 15, 16, 17, 18, 19, 20, 21, 22, 23, 24}, Cv][[2, 2]]],
   RefineEs[x, 2, 4, {1, 2, 3, 4, 5, 6, 7, 8, 10, 11, 12, 13, 14, 15, 16, 17, 18, 19, 20, 21, 22, 23, 24}, Es][[2]],
   RefineEs[x, 2, 4, {1, 2, 3, 4, 5, 6, 7, 8, 10, 11, 12, 13, 14, 15, 16, 17, 18, 19, 20, 21, 22, 23, 24}, Es][[2]] +
    1.96 * Sqrt[RefineVar[x, 2, 4, {1, 2, 3, 4, 5, 6, 7, 8, 10, 11, 12, 13, 14, 15, 16, 17, 18, 19, 20, 21, 22, 23, 24}, Cv][[2, 2]]]},
  {x, 1, 24}, Filling → {1 → {3}}, AxesLabel → {"Month Since 2012", "Coefficient of Temperature"}, ImageSize → Large,
  Exclusions → {1, 2, 3, 4, 5, 6, 7, 8, 9, 10, 11, 12, 13, 14, 15, 16, 17, 18, 19, 20, 21, 22, 23, 24}, PlotLabel → "(A)"]
```

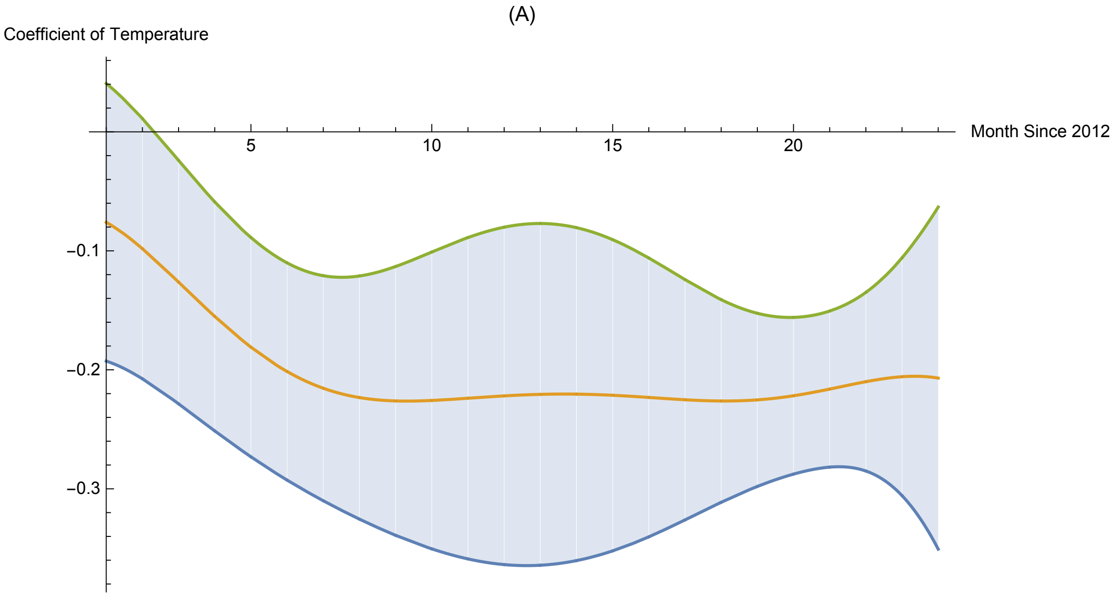

```
(*For coefficient of precipitation*)
Plot[{RefineEs[x, 2, 4, {1, 2, 3, 4, 5, 6, 7, 8, 10, 11, 12, 13, 14, 15, 16, 17, 18, 19, 20, 21, 22, 23, 24}, Es][[
      3(*precipitation is the third element of the estimator*)]] -
    1.96 * Sqrt[RefineVar[x, 2, 4, {1, 2, 3, 4, 5, 6, 7, 8, 10, 11, 12, 13, 14, 15, 16, 17, 18, 19, 20, 21, 22, 23, 24}, Cv][[3, 3]]],
   RefineEs[x, 2, 4, {1, 2, 3, 4, 5, 6, 7, 8, 10, 11, 12, 13, 14, 15, 16, 17, 18, 19, 20, 21, 22, 23, 24}, Es][[3]],
   RefineEs[x, 2, 4, {1, 2, 3, 4, 5, 6, 7, 8, 10, 11, 12, 13, 14, 15, 16, 17, 18, 19, 20, 21, 22, 23, 24}, Es][[3]] +
    1.96 * Sqrt[RefineVar[x, 2, 4, {1, 2, 3, 4, 5, 6, 7, 8, 10, 11, 12, 13, 14, 15, 16, 17, 18, 19, 20, 21, 22, 23, 24}, Cv][[3, 3]]]},
  {x, 1, 24}, Filling → {1 → {3}}, AxesLabel → {"Month Since 2012", "Coefficient of Precipitation"}, ImageSize → Large,
  Exclusions → {1, 2, 3, 4, 5, 6, 7, 8, 9, 10, 11, 12, 13, 14, 15, 16, 17, 18, 19, 20, 21, 22, 23, 24}, PlotLabel → "(B)"]
```

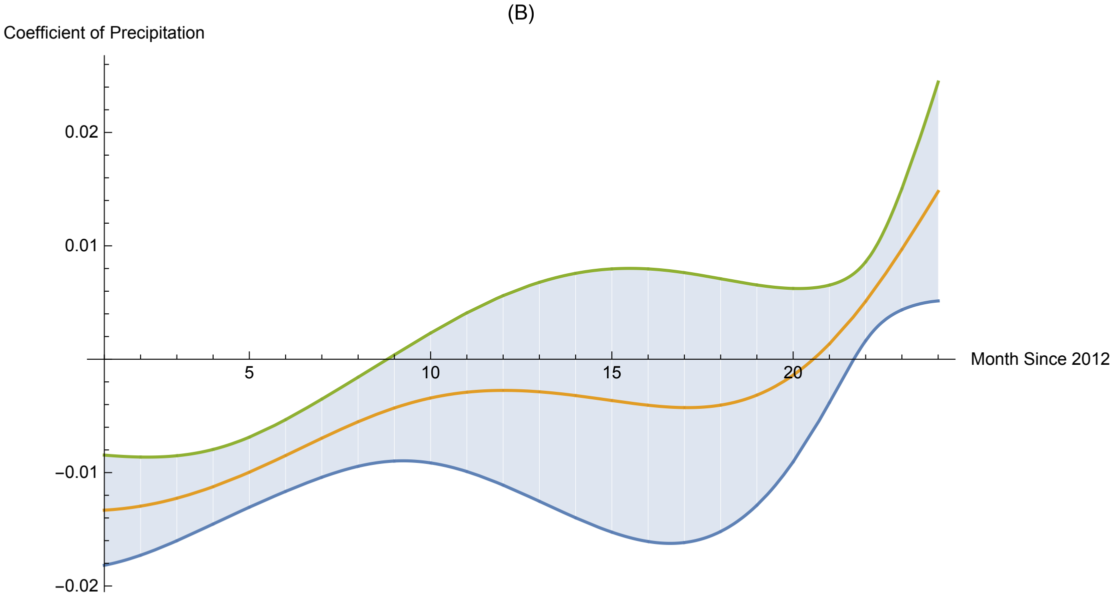


```
(*For coefficient of RH*)
Plot[{RefineEs[x, 2, 4, {1, 2, 3, 4, 5, 6, 7, 8, 10, 11, 12, 13, 14, 15, 16, 17, 18, 19, 20, 21, 22, 23, 24}, Es][[
      4(*RH is the fourth element of the estimator*)]] -
    1.96 * Sqrt[RefineVar[x, 2, 4, {1, 2, 3, 4, 5, 6, 7, 8, 10, 11, 12, 13, 14, 15, 16, 17, 18, 19, 20, 21, 22, 23, 24}, Cv][[4, 4]]],
   RefineEs[x, 2, 4, {1, 2, 3, 4, 5, 6, 7, 8, 10, 11, 12, 13, 14, 15, 16, 17, 18, 19, 20, 21, 22, 23, 24}, Es][[4]],
   RefineEs[x, 2, 4, {1, 2, 3, 4, 5, 6, 7, 8, 10, 11, 12, 13, 14, 15, 16, 17, 18, 19, 20, 21, 22, 23, 24}, Es][[4]] +
    1.96 * Sqrt[RefineVar[x, 2, 4, {1, 2, 3, 4, 5, 6, 7, 8, 10, 11, 12, 13, 14, 15, 16, 17, 18, 19, 20, 21, 22, 23, 24}, Cv][[4, 4]]]},
  {x, 1, 24}, Filling → {1 → {3}}, AxesLabel → {"Month Since 2012", "Coefficient of Relative Humidity"}, ImageSize → Large,
  Exclusions → {1, 2, 3, 4, 5, 6, 7, 8, 9, 10, 11, 12, 13, 14, 15, 16, 17, 18, 19, 20, 21, 22, 23, 24}, PlotLabel → "(C)"]
```

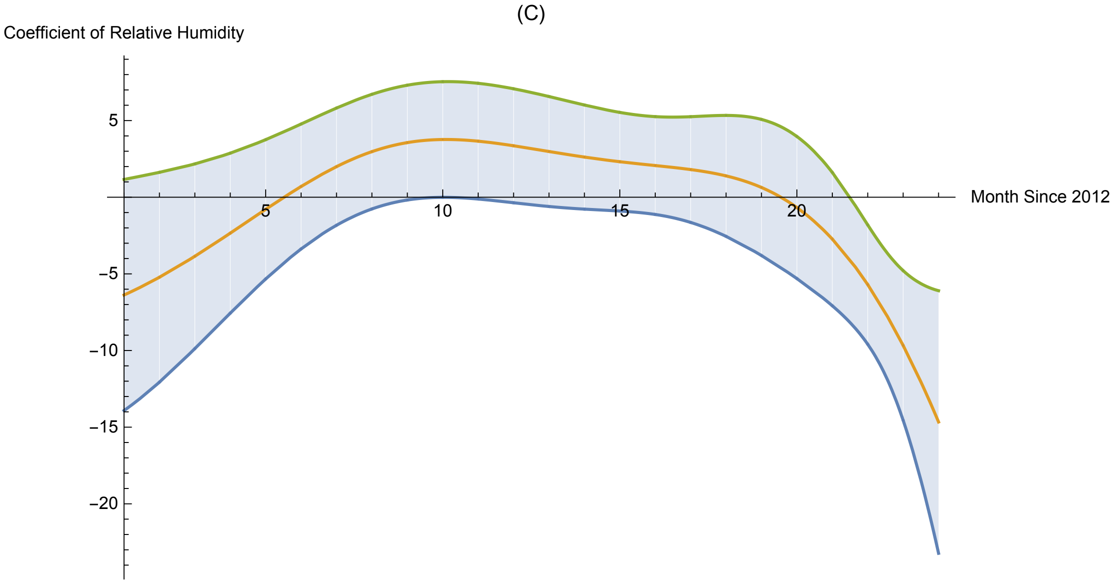

Now we show the example of Thailand as what we present in the paper, the process is same as France

Note that the geographical center of Thailand has coordinates (15,100), and the temporal correlation parameter is 0.19

```
Es2 = RawEs[{25, 26, 27, 28, 29, 30, 31, 32, 34, 35, 36, 37, 38, 39, 40, 41, 42, 43, 44, 45, 46, 47, 48}, 3, 0.19, 15, 100, 1450, DA];
Cv2 = RawCv[{25, 26, 27, 28, 29, 30, 31, 32, 34, 35, 36, 37, 38, 39, 40, 41, 42, 43, 44, 45, 46, 47, 48}, 3, 0.19, 15, 100, 1450, Es2, DA];

(*For coefficient of temperature*)
Plot[{RefineEs[x, 2, 3, {1, 2, 3, 4, 5, 6, 7, 8, 10, 11, 12, 13, 14, 15, 16, 17, 18, 19, 20, 21, 22, 23, 24}, Es2][[2]] −
     1.96 * Sqrt[RefineVar[x, 2, 3, {1, 2, 3, 4, 5, 6, 7, 8, 10, 11, 12, 13, 14, 15, 16, 17, 18, 19, 20, 21, 22, 23, 24}, Cv2][[2, 2]]],
   RefineEs[x, 2, 3, {1, 2, 3, 4, 5, 6, 7, 8, 10, 11, 12, 13, 14, 15, 16, 17, 18, 19, 20, 21, 22, 23, 24}, Es2][[2]],
   RefineEs[x, 2, 3, {1, 2, 3, 4, 5, 6, 7, 8, 10, 11, 12, 13, 14, 15, 16, 17, 18, 19, 20, 21, 22, 23, 24}, Es2][[2]] +
     1.96 * Sqrt[RefineVar[x, 2, 3, {1, 2, 3, 4, 5, 6, 7, 8, 10, 11, 12, 13, 14, 15, 16, 17, 18, 19, 20, 21, 22, 23, 24}, Cv2][[2, 2]]]},
  {x, 1, 24}, Filling → {1 → {3}}, AxesLabel → {"Month Since 2012", "Coefficient of Temperature"}, ImageSize → Large,
  Exclusions → {1, 2, 3, 4, 5, 6, 7, 8, 9, 10, 11, 12, 13, 14, 15, 16, 17, 18, 19, 20, 21, 22, 23, 24}, PlotLabel → "(A)"]
```

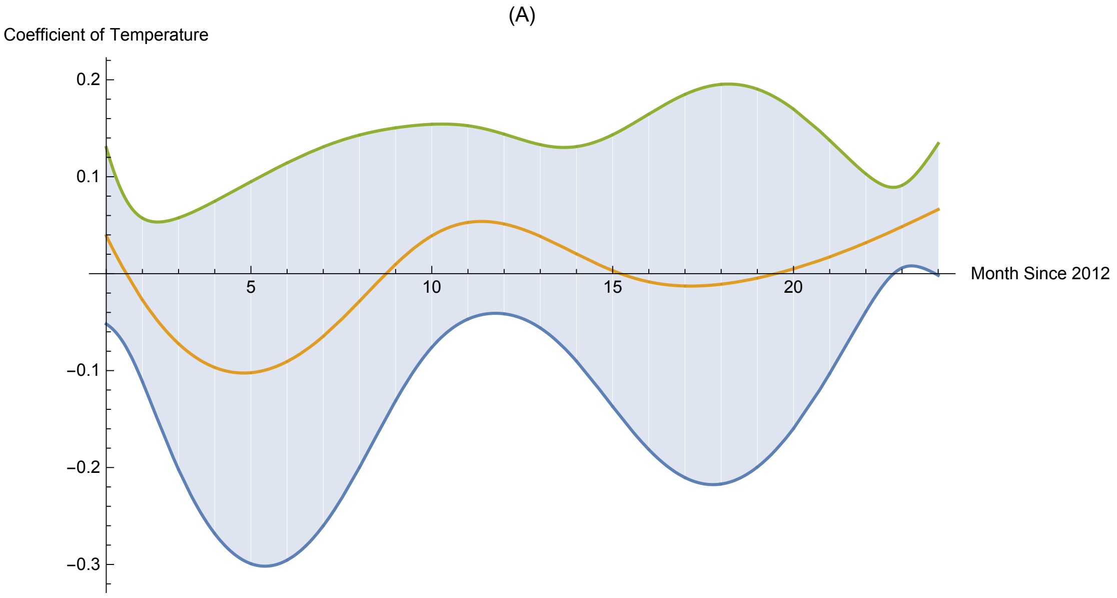

```
(*For coefficient of precipitation*)
Plot[{RefineEs[x, 2, 3, {1, 2, 3, 4, 5, 6, 7, 8, 10, 11, 12, 13, 14, 15, 16, 17, 18, 19, 20, 21, 22, 23, 24}, Es2][[3]] −
     1.96 * Sqrt[RefineVar[x, 2, 3, {1, 2, 3, 4, 5, 6, 7, 8, 10, 11, 12, 13, 14, 15, 16, 17, 18, 19, 20, 21, 22, 23, 24}, Cv2][[3, 3]]],
   RefineEs[x, 2, 3, {1, 2, 3, 4, 5, 6, 7, 8, 10, 11, 12, 13, 14, 15, 16, 17, 18, 19, 20, 21, 22, 23, 24}, Es2][[3]],
   RefineEs[x, 2, 3, {1, 2, 3, 4, 5, 6, 7, 8, 10, 11, 12, 13, 14, 15, 16, 17, 18, 19, 20, 21, 22, 23, 24}, Es2][[3]] +
     1.96 * Sqrt[RefineVar[x, 2, 3, {1, 2, 3, 4, 5, 6, 7, 8, 10, 11, 12, 13, 14, 15, 16, 17, 18, 19, 20, 21, 22, 23, 24}, Cv2][[3, 3]]]},
  {x, 1, 24}, Filling → {1 → {3}}, AxesLabel → {"Month Since 2012", "Coefficient of Precipitation"}, ImageSize → Large,
  Exclusions → {1, 2, 3, 4, 5, 6, 7, 8, 9, 10, 11, 12, 13, 14, 15, 16, 17, 18, 19, 20, 21, 22, 23, 24}, PlotLabel → "(B)"]
```

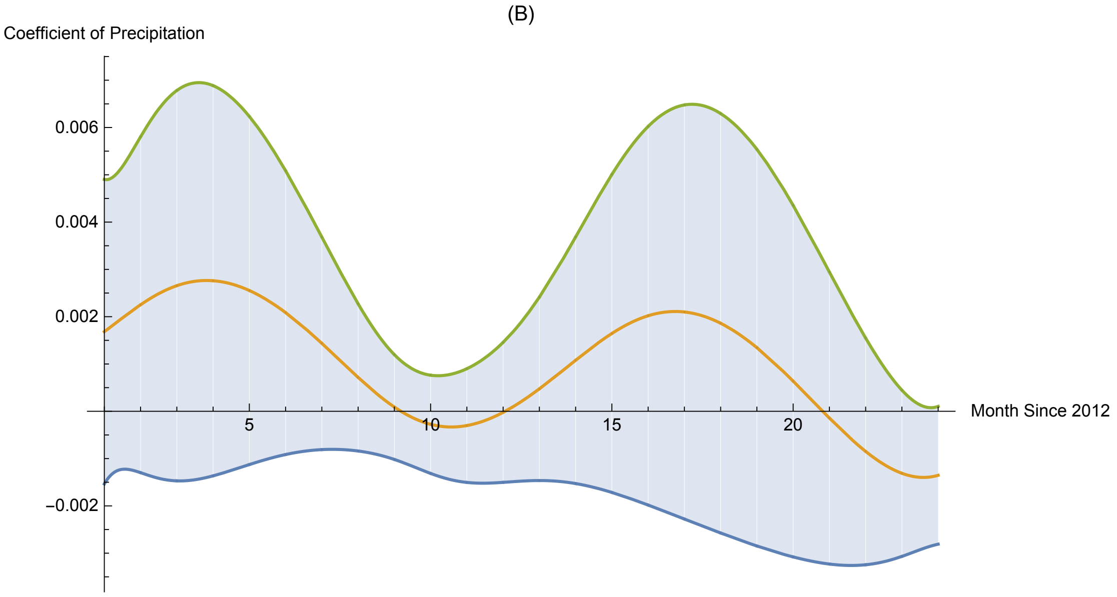


```
(*For coefficient of RH*)
Plot[{RefineEs[x, 2, 3, {1, 2, 3, 4, 5, 6, 7, 8, 10, 11, 12, 13, 14, 15, 16, 17, 18, 19, 20, 21, 22, 23, 24}, Es2][[4]] −
     1.96 * Sqrt[RefineVar[x, 2, 3, {1, 2, 3, 4, 5, 6, 7, 8, 10, 11, 12, 13, 14, 15, 16, 17, 18, 19, 20, 21, 22, 23, 24}, Cv2][[4, 4]]],
   RefineEs[x, 2, 3, {1, 2, 3, 4, 5, 6, 7, 8, 10, 11, 12, 13, 14, 15, 16, 17, 18, 19, 20, 21, 22, 23, 24}, Es2][[4]],
   RefineEs[x, 2, 3, {1, 2, 3, 4, 5, 6, 7, 8, 10, 11, 12, 13, 14, 15, 16, 17, 18, 19, 20, 21, 22, 23, 24}, Es2][[4]] +
     1.96 * Sqrt[RefineVar[x, 2, 3, {1, 2, 3, 4, 5, 6, 7, 8, 10, 11, 12, 13, 14, 15, 16, 17, 18, 19, 20, 21, 22, 23, 24}, Cv2][[4, 4]]]},
  {x, 1, 24}, Filling → {1 → {3}}, AxesLabel → {"Month Since 2012", "Coefficient of Relative Humidity"}, ImageSize → Large,
  Exclusions → {1, 2, 3, 4, 5, 6, 7, 8, 9, 10, 11, 12, 13, 14, 15, 16, 17, 18, 19, 20, 21, 22, 23, 24}, PlotLabel → "(C)"]
```

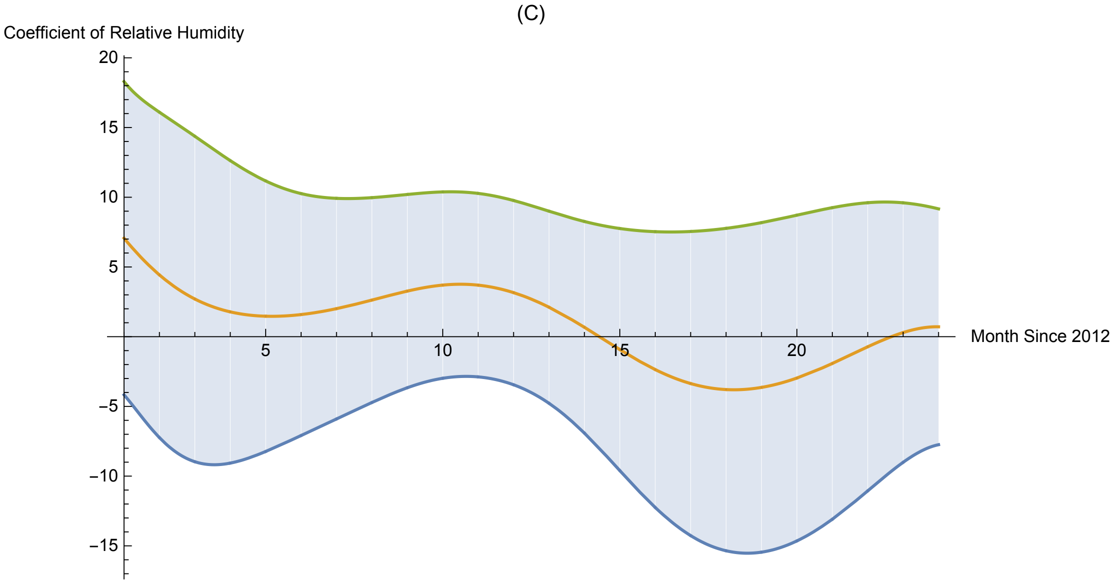

Now we show how to project these result to the map

Please first run the following code to import the raw estimator of the 22 countries

```
Es1 = {{4.3103790750280755`, −0.09347943468376584`, −0.012856250085036752`, −6.010140404085008`},
   {3.8063808224067093`, −0.08257602302979479`, −0.013530860694731032`, −5.384515284270297`},
   {2.8875796667110185`, −0.07217296018150102`, −0.014575362686395253`, −4.167394276079998`},
   {2.638777990665243`, −0.08352180227361924`, −0.011330857083901625`, −3.8840146877605544`},
   {2.757436722652374`, −0.10271030572445354`, −0.009126044750950569`, −3.908420899988653`},
   {0.2506747018476168`, −0.23066058687164204`, −0.01497970618866562`, 1.617265406425387`},
   {5.716866584881538`, −0.38211626167156765`, −0.011996933702432067`, −4.149101374670637`},
   {−3.592399378560568`, −0.08996111868408274`, 0.002218711725136751`, 0.5122139358643778`},
   {−10.132973805971403`, −0.17100211226275952`, 0.002874614394602568 7`, 10.266862811992262`},
   {−1.5301437848488224`, −0.17590826499043113`, −0.0036987657824508304`, 1.5435083087051047`},
   {0.011148624851505539`, −0.17115255538160973`, −0.0012837956532694283`, −0.4166337916683993`},
   {0.32137462427711866`, −0.15357835696515842`, −0.00290370561466325 8`, −0.6661671257330513`},
   {0.029080250896879056`, −0.1485554601702332`, −0.0027569877261937976`, −0.34577219757636235`},
   {−0.8100617624677771`, −0.12673066928685994`, −0.0024590293119852082`, 0.6243823171547414`},
   {−1.6116467221206545`, −0.12448118601485417`, −0.00030055726289942745`, 1.4789962659836184`},
   {−2.5218947202878894`, −0.14841475149411407`, 0.00108906549278368`, 2.7135490744475677`},
   {−0.3215602341174306`, −0.21251924852554513`, 0.003697499472991376`, −0.030583920064506078`},
   {5.743422727124703`, −0.36945061742234075`, −0.01782283639639022`, −4.221924681256512`},
   {−1.9079090527964557`, −0.0946591844475363`, 0.005938326634114265`, −2.496679280685416`},
   {−5.361981720682678`, −0.11308758326096915`, 0.0011856185038383892`, 2.547362407914159`},
   {3.1139311842570656`, −0.18339458370040967`, 0.011443504804660902`, −7.158908471971672`},
   {7.38360900965705`, −0.19203949830218303`, 0.011213180950898492`, −11.500011910084428`},
   {7.293838857555111`, −0.15844940567579802`, 0.008914834055786783`, −11.212460573572491`}};
Es2 = {{3.8888186150737822`, −0.0800958366210289`, −0.012700215686602314`, −5.551819073406812`},
   {3.4040922673396286`, −0.0702531013990767`, −0.01337348965860535 8`, −4.939780295686611`},
   {2.4332841783093273`, −0.05946557840922183`, −0.01492877781419557`, −3.6195249271862773`},
   {2.203357387917137`, −0.07009299989344064`, −0.0120026636954418 6`, −3.347343836723367`},
   {2.440160067482059`, −0.09046397913772813`, −0.009829369904128643`, −3.5171646099107496`},
   {0.2750697634473079`, −0.20633799903546926`, −0.015163500304869116`, 1.3131572006099252`},
   {5.717764177453933`, −0.36119895559337767`, −0.010453349899900877`, −4.596259316564883`},
   {−3.139731774352354`, −0.09039196968086695`, 0.0015062348405209599`, 0.14766547389324555`},
   {−9.82610435055227`, −0.15551386008119225`, 0.0033674677274289438`, 9.791813704141392`},
   {−1.450265604790142`, −0.15645741215044043`, −0.003237704270114152`, 1.336076830902103`},
   {−0.09920902939810337`, −0.1499014169779841`, −0.001070684391688141`, −0.3719436193470835`},
   {0.2238421906326439`, −0.13174047541722655`, −0.002663643152820062`, −0.6341950027307447`},
   {−0.0819033722316101`, −0.12645240870926466`, −0.0025467349756147857`, −0.29330615023721035`},
   {−0.9192415106292496`, −0.1069261765623652`, −0.002186828539889865`, 0.6814086251914836`},
   {−1.6283737925898087`, −0.10757748387399352`, −0.00023887060107892507`, 1.4519364637289203`},
   {−2.3464347754842545`, −0.13489086088627655`, 0.0009070543410230785`, 2.4816786094931933`},
   {0.35058534242818334`, −0.1951787145771357`, 0.0037130536397964957`, −0.9968114904817613`},
   {6.65657801061383`, −0.3485149073267328`, −0.014966342938341799`, −5.92712570079975`},
   {−0.11286885061339533`, −0.10393385918596264`, 0.006435821081483205`, −4.549437820838602`},
   {−4.630179484554941`, −0.10730243810414376`, 0.0020580571232003088`, 1.5233492762349503`},
   {3.155033509089904`, −0.17461145132390407`, 0.01115201804601052`, −7.245283034555096`},
   {6.7713007517415384`, −0.18152817985313519`, 0.010370127429767895`, −10.711233385699309`},
   {6.925961939242277`, −0.14507957002553795`, 0.008084856557472988`, −10.72800213240081`}};
Es3 = {{3.367153334510832`, −0.07104614652369946`, −0.012819191323157808`, −4.908616500546991`},
   {2.869443191833921`, −0.06068555890397713`, −0.013488946237894123`, −4.284716642360007`},
   {1.807770756824798`, −0.04826173500776917`, −0.01516292986628825 3`, −2.852015764382561`},
   {1.6695708478691529`, −0.06098013353484424`, −0.012250961264718143`, −2.685075745693242`},
   {1.9109903792375023`, −0.08178281559167257`, −0.010129248502427534`, −2.854011231626325`},
```

```
{−0.22639000876672175`, −0.20476136782194515`, −0.015163066898477866`, 1.9972379838270016`},
{5.363219365476577`, −0.3569569074512709`, −0.010021546746547478`, −4.2064014043301245`},
{−3.3514391127959624`, −0.08543662045390077`, 0.0009943359186073261`, 0.5181091926997365`},
{−10.389819212541887`, −0.14828995231009046`, 0.0029492045705594824`, 10.429022236580035`},
{−1.8294010134715906`, −0.15247831549656826`, −0.003121627073035584`, 1.7675135396805552`},
{−0.27565308504932223`, −0.1465405210311227`, −0.001578119270154725`, −0.104233388900725`},
{0.03039871604742035`, −0.1269085352485253`, −0.003154703953850551`, −0.3469609445245941`},
{−0.28884548171987323`, −0.12166462183317875`, −0.003025347551096986`, 0.008525827463931874`},
{−1.1218045853367102`, −0.1031417918036863`, −0.002564045702354308`, 0.970588719726835`},
{−1.6318191120901178`, −0.10666039367111971`, −0.0009204398681249796`, 1.531811560048401`},
{−2.201119611779324`, −0.1384162538378741`, −0.00014954835784114468`, 2.426381660999198`},
{0.33782263937507373`, −0.19626902089490766`, 0.002439373160702568`, −0.8235016468340481`},
{6.415059406258813`, −0.3624973026146968`, −0.01433831429638838`, −5.435096823084506`},
{0.8706422250016179`, −0.11899285662429393`, 0.005658706433212096`, −5.34990388178099`},
{−4.7606632767671115`, −0.10071631350642388`, 0.0023625439565873505`, 1.5958196832539318`},
{3.045747092406821`, −0.1755693326975927`, 0.010648357389939349`, −7.073162100845524`},
{6.984073203485453`, −0.18748110113153735`, 0.010330010862954604`, −10.927025130552536`},
{6.805420759484076`, −0.14620094424106214`, 0.0077802329606673675`, −10.505559721065627`}};
Es4 = {{4.121204393510064`, −0.0925540551222127`, −0.012939693213845312`, −5.759187779883073`},
{3.598349331420516`, −0.08082937778832903`, −0.01362671834416338`, −5.1162489814033645`},
{2.6355193653719353`, −0.06944340944379758`, −0.014581954399842266`, −3.857653159708239`},
{2.4300188643781113`, −0.08227154672477457`, −0.011209196912409977`, −3.627170335679289`},
{2.500990302045342`, −0.10114963387439643`, −0.009010654720712311`, −3.589437656995438`},
{−0.07035615110198334`, −0.23791818205755544`, −0.014931062793033125`, 2.148408288521682`},
{5.478745781995843`, −0.38576875710209957`, −0.012266378370072797`, −3.73946064137818`},
{−4.091184875474966`, −0.08395380342559619`, 0.0019687601021341748`, 1.116229588815853`},
{−10.621753578316627`, −0.17222040049126808`, 0.0024945814395506355`, 10.861086191105535`},
{−1.8703737083570173`, −0.18108725946911347`, −0.003704114509598711`, 1.968932190849872`},
{−0.10103013340568197`, −0.17766236100652605`, −0.0016245132297037417`, −0.2158816824578218`},
{0.2143703644068153`, −0.15904775482537417`, −0.003263937191766321`, −0.4713362410569055`},
{−0.07780597494916658`, −0.15406329228288226`, −0.003106385316959204`, −0.15278877841037206`},
{−0.9140722376154121`, −0.1317105475578645`, −0.0027949708429918538`, 0.8086401181724208`},
{−1.6064610316804497`, −0.13006228133061673`, −0.0008430592323961317`, 1.5493553481027025`},
{−2.4418730208052937`, −0.15564390232283484`, 0.0003664924842222886`, 2.7130847290685463`},
{−0.5486149319190042`, −0.21951297679878096`, 0.0027414511049558454`, 0.41852797543693226`},
{5.303519284305773`, −0.3839847190440147`, −0.01836032168926792`, −3.3864447988016004`},
{−1.7945183201913752`, −0.1021585308263159`, 0.005087321949078795`, −2.390636212156205`},
{−5.741956982700421`, −0.11045443145037023`, 0.001114213370169746`, 2.990943928251542`},
{3.0074606211283914`, −0.18674501486723735`, 0.011111126915101277`, −6.979197450546001`},
{7.7326216984521645`, −0.19952092931923102`, 0.011426179315580244`, −11.902976401524311`},
{7.327233811094181`, −0.16421814133090695`, 0.008907615276544574`, −11.20129974342434`}};
Es5 = {{3.414192863543515`, −0.0925621857982154`, −0.012774556908721302`, −4.812041234905586`},
{2.764267552037932`, −0.07610413779915508`, −0.013563250431121934`, −4.047037177901257`},
{1.4868808191369138`, −0.05783586637941541`, −0.014258487586869257`, −2.4734609559704297`},
{1.5756873229238535`, −0.08013785349111519`, −0.01017996097142959`, −2.58871764637014`},
{1.5083687836340685`, −0.09931265268779085`, −0.008011198286956936`, −2.3598082174345376`},
{−0.9052316603234132`, −0.278582263115187`, −0.014701094173037532`, 3.768577641793453`},
{4.8459757403686`, −0.4004054200241587`, −0.013537508620606322`, −2.5085748136561707`},
{−5.8287005165874115`, −0.06381564585298011`, 0.0007217432032444607`, 3.2592881345348057`},
{−13.321249421218525`, −0.1868855815540351`, 0.0008280995036256558`, 14.151812942117381`},
{−3.1996883451153377`, −0.22234910690841422`, −0.0035290961108086056`, 3.7217179229848996`},
{−0.42219610197426033`, −0.22587180266057622`, −0.0021659676023466712`, 0.4805193558161953`},
{−0.05485755834412706`, −0.2007533432246846`, −0.0038399969516754494`, 0.1519303685639641`},
{−0.3371322693930685`, −0.1956063716929269`, −0.0036742551139536043`, 0.45352265962464594`},
{−1.1804783891788357`, −0.16842056992028615`, −0.003401596255934985`, 1.3949141615599157`},
```

```
        {−1.5503180427576992`, −0.16545659429727472`, −0.0024625805307786942`, 1.8208275837167638`},
        {−2.049218085711466`, −0.1939444250802396`, −0.002753122743766805`, 2.7066514467064295`},
        {−1.6036053633088925`, −0.2588591800602665`, −0.002497729300069334`, 2.640240586089156`},
        {3.7071623255092603`, −0.4356864290731326`, −0.020833153247997886`, −0.39445289405975137`},
        {−1.0037931469289625`, −0.13408909722191562`, 0.001965836247227951`, −2.4269848301368304`},
        {−7.012977308251612`, −0.10505319869360205`, 0.000779028373202364`, 4.509277552541969`},
        {2.4362192464383816`, −0.2044257632058189`, 0.00982774764428368`, −6.046315683200223`},
        {10.38439550835637`, −0.24416610552059234`, 0.013100089152482484`, −15.003741032465319`},
        {7.724618740915079`, −0.19874209282142127`, 0.008797139264468058`, −11.360911990476666`}};
Es6 = {{4.5831867029463025`, −0.09302787898427993`, −0.01248567675072682`, −6.39429420767235`},
        {4.112040073568708`, −0.0836843434402713`, −0.01315019706623841`, −5.797239718721936`},
        {3.246611565665256`, −0.07471078934958093`, −0.014472576027816954`, −4.6168291954949625`},
        {2.930674541758796`, −0.08361083924248587`, −0.011491555543414161`, −4.2475793894327545`},
        {3.1728469956199508`, −0.10391106579949817`, −0.009286030821707248`, −4.429472328922515`},
        {0.9799616520050064`, −0.21409756070632796`, −0.015096224795123533`, 0.4054396145202635`},
        {6.2958472446707034`, −0.37077434641896373`, −0.01122642954613373`, −5.185131335977044`},
        {−2.4695496000462605`, −0.1022696864647481`, 0.002404577132691406`, −0.7964065058501292`},
        {−9.270409474066213`, −0.16661345422241514`, 0.003741896841747063`, 9.190637497252208`},
        {−0.8540928673763413`, −0.1647196985091306`, −0.003554456051401168`, 0.6892311974037549`},
        {0.21055490499904278`, −0.15710873726267635`, −0.0004361298846753127`, −0.7965340085071284`},
        {0.517517356207522`, −0.14114794886191062`, −0.0019988532700197107`, −1.0444843866178786`},
        {0.22305910503589946`, −0.1360004344406614`, −0.0018857451024262145`, −0.7168482940998298`},
        {−0.623396259598239`, −0.11541439664846416`, −0.001612697024871531`, 0.27297198502333686`},
        {−1.622825945437165`, −0.11212902789093673`, 0.0007795921729165304`, 1.3471302565809498`},
        {−2.6059868568253437`, −0.13348453529961923`, 0.002305818422304252`, 2.650860118884166`},
        {0.22408700731876344`, −0.19768978159768585`, 0.005290145147617687`, −1.0088936512338678`},
        {6.916409237482304`, −0.33649866743726004`, −0.016263100896750093`, −6.398974477904309`},
        {−1.5547991171987743`, −0.0837945034228882`, 0.007711111102128407`, −3.366783360812163`},
        {−4.3706992132142`, −0.11859179459785203`, 0.001509333830061433`, 1.362629257775387`},
        {3.288764351193261`, −0.17579883960980155`, 0.012026502889861304`, −7.4657686538595875`},
        {6.761168395201504`, −0.17759400026982902`, 0.010728514806408792`, −10.764338621896803`},
        {7.183900368441053`, −0.146389459967872`, 0.008708424301052849`, −11.145525659040487`}};
Es7 = {{3.4472127386274485`, −0.08686501919440894`, −0.012946298859155195`, −4.89005910423097`},
        {2.8654387465379854`, −0.0727064333767838`, −0.013671588394005915`, −4.193004348559473`},
        {1.711274074392409`, −0.057363788328483906`, −0.014524685293287341`, −2.7342275739955766`},
        {1.6961285158057073`, −0.07588825994367487`, −0.010802664447958112`, −2.727518599039411`},
        {1.7080992885347714`, −0.09528126051409998`, −0.008647118707113646`, −2.6020000847964284`},
        {−0.7524537759073008`, −0.25702102150911516`, −0.01483346590879256`, 3.315596198253873`},
        {4.98182930027445`, −0.39047837475739405`, −0.012758365864925427`, −2.920084244151972`},
        {−5.091332898377149`, −0.07109257877207664`, 0.000938155241375945`, 2.425535005057815`},
        {−12.207314333011015`, −0.17337922494525415`, 0.001494056791229657`, 12.755621432622565`},
        {−2.7300224516933214`, −0.19641674090846067`, −0.0035834023308104175`, 3.056876115759789`},
        {−0.3807421999181487`, −0.19687666660306452`, −0.0022241584847309537`, 0.29697489521741843`},
        {−0.04603915577432312`, −0.17453415897926894`, −0.003884453898849003`, 0.015059458488290982`},
        {−0.34376329756338364`, −0.16956494058643032`, −0.0037086897087446453`, 0.3363354475373661`},
        {−1.1806705407344646`, −0.14597225431897468`, −0.003337397751542322`, 1.2818293760398851`},
        {−1.5948922915833472`, −0.1456047939671991`, −0.002106302735261766`, 1.748308573373668`},
        {−2.1624044301620913`, −0.17589885458685445`, −0.0018582459034688927`, 2.682796672008596`},
        {−1.0502925675184078`, −0.23914981883575334`, −0.00044587652796230994`, 1.566521323058338`},
        {4.412317672287717`, −0.41748704016680943`, −0.019361662249381448`, −1.667535042621008`},
        {−0.8130640585364053`, −0.12675667953222863`, 0.0031122550806611 9`, −2.9030168041786393`},
        {−6.395301659659311`, −0.10459913496673645`, 0.001153705599081302`, 3.725184176230549`},
        {2.6661623636469147`, −0.19554272210779086`, 0.010172583959103688`, −6.422764383982875`},
        {8.991501565144885`, −0.22307154570853005`, 0.012093382867875674`, −13.34536404475745`},
        {7.4016562936327`, −0.18074613005634207`, 0.008594918782618104`, −11.086302537269017`}};
```

```
Es8 = {{4.405512364361346`, -0.11308463775087761`, -0.01266656875663161`, -6.001592098976982`},
    {3.721597941760509`, -0.09605725061126119`, -0.013509932801315377`, -5.195466260314368`},
    {2.5771402063689437`, -0.07978003328061702`, -0.013907196584387412`, -3.7957522438121933`},
    {2.4747242474648075`, -0.09844002028038637`, -0.009732711920720468`, -3.6943615292121494`},
    {2.22271776566794`, -0.1141163771848122`, -0.007496452719378307`, -3.259952533208452`},
    {-0.671479555714288`, -0.2915415750102334`, -0.014521848024502425`, 3.5788941139091923`},
    {5.067591121365116`, -0.4180963084633842`, -0.014397497207228825`, -2.5047786940704624`},
    {-5.976365813148384`, -0.06540619341067155`, 0.002303235794423223`, 3.100428414844203`},
    {-13.001881470886541`, -0.21001673810640417`, 0.0008598909600291833`, 13.90953296004907`},
    {-3.0752676166790702`, -0.2419697489878279`, -0.0037994948452424993`, 3.6489589741498785`},
    {-0.22960813601639993`, -0.24338148037875929`, -0.001974688245047009`, 0.2596699043685518`},
    {0.128378430400368`, -0.22012472474905107`, -0.003640703412693398`, -0.06331476165849853`},
    {-0.11945808644422128`, -0.21518247682981853`, -0.0034947657847609116`, 0.1994924986692355`},
    {-0.9528604779114669`, -0.18397280394719828`, -0.003467726818929546`, 1.1316802363565992`},
    {-1.487894690022602`, -0.17687687653263`, -0.0020770377375293734`, 1.7014607351169633`},
    {-2.212133274485438`, -0.19881298533453842`, -0.0014560915828436514`, 2.7519346758839265`},
    {-2.0708697189542256`, -0.26654656523079995`, -0.001191151353241173`, 3.126257029843373`},
    {3.3397369392526763`, -0.4368893719728386`, -0.022449156747747435`, 0.2172477086886741`},
    {-3.097556029833885`, -0.1140872653010647`, 0.001896128015493125`, -0.26201395059343424`},
    {-7.76106710906796`, -0.11007393464738517`, -0.00026324207964359934`, 5.558018748812872`},
    {2.5334896841306356`, -0.20926646804340815`, 0.010490118005729224`, -6.1886101355370755`},
    {10.714353357791076`, -0.2465751230192387`, 0.013866036837909268`, -15.497189842964493`},
    {8.239905098989214`, -0.20668714214436537`, 0.010091887837273328`, -12.154274590701693`}};
Es9 = {{3.1211960579217735`, -0.0849029610473364`, -0.012866412357583009`, -4.46481392821235`},
    {2.4977549757210866`, -0.06917400044339611`, -0.013614274889988887`, -3.729318488301205`},
    {1.1977252947378338`, -0.05077926617333176`, -0.014451020699195422`, -2.1167284754315205`},
    {1.3311141125932626`, -0.07350150245540814`, -0.01050849381319897`, -2.2809268943664054`},
    {1.3444217636708495`, -0.09392703160703669`, -0.008365443941724346`, -2.1457092242786553`},
    {-0.9599932627168711`, -0.2701812563710551`, -0.014776563354822515`, 3.753127215049383`},
    {4.819216358951371`, -0.39277745635816075`, -0.013060570533770141`, -2.6186329255355267`},
    {-5.45385858585044`, -0.06678424840571288`, 0.0003829962249020092`, 2.936199201168237`},
    {-13.234704053698126`, -0.1765809133584132`, 0.0009230347765775707`, 13.990025904717958`},
    {-3.1159322784065355`, -0.2103527024160508`, -0.003486478905179379`, 3.580245007488149`},
    {-0.457086606235043`, -0.21350408056591008`, -0.0022834545829324053`, 0.49490048828122035`},
    {-0.10404450837526588`, -0.1885093511519399`, -0.00394757981491262`, 0.1860764385386451`},
    {-0.40286789937050865`, -0.18344959176413744`, -0.0037713884934861682`, 0.5069477868585972`},
    {-1.2471102995342713`, -0.15851319525887295`, -0.003387848071747128`, 1.450126795887452`},
    {-1.5695171765408154`, -0.1577078990465984`, -0.0024918981514239318`, 1.8305045584692428`},
    {-2.021523863908313`, -0.18893152340802344`, -0.0029635673721896023`, 2.688011743088103`},
    {-1.3375808247392549`, -0.252476810794166`, -0.0024530564677793304`, 2.2565568486288092`},
    {3.9789512835197662`, -0.43232067083122167`, -0.01998207694552231`, -0.8565152080683648`},
    {-0.2459953846166952`, -0.1392248273102338`, 0.0023054144332467705`, -3.281897359247397`},
    {-6.631461123660552`, -0.10324689444502112`, 0.001167514067200874`, 3.996697479226334`},
    {2.4621689723336204`, -0.2013255509069664`, 0.009736410889841337`, -6.095360634936822`},
    {10.041893080625597`, -0.23969184594675158`, 0.012701530741953664`, -14.566457451398971`},
    {7.525715217051317`, -0.1926159598044522`, 0.008405640578587344`, -11.095601728529541`}};
Es10 = {{3.7066737054483765`, -0.08261608678205457`, -0.012942497087564944`, -5.2741051497385225`},
    {3.1923517171136475`, -0.07123294919298812`, -0.013620283431791303`, -4.638751300934953`},
    {2.1759853413470065`, -0.059152783019881236`, -0.014822142348022104`, -3.2978853803199617`},
    {2.0200839836464817`, -0.07257309586616892`, -0.011584314100379396`, -3.1206698148813143`},
    {2.1537910325872214`, -0.092299878075407`, -0.0094247689054841`, -3.156235844013525`},
    {-0.2306189074101163`, -0.22650399378238958`, -0.015025284269495252`, 2.2476565060348945`},
    {5.351499861165833`, -0.3747028842971134`, -0.011480947122104186`, -3.7934391708354798`},
    {-3.9657953345917205`, -0.08255538887171723`, 0.0013786936367332428`, 1.1035168977035448`},
    {-10.666594020494488`, -0.16165332682265637`, 0.0025704581484728467`, 10.848495085808198`},
```

```
        {−1.9586802705375117`, −0.17005299467335347`, −0.0034638770516365676`, 2.014965927883477`},
        {−0.21249845702644782`, −0.16628815045959`, −0.0017095271250636315`, −0.10362573306408679`},
        {0.105058764174657`, −0.14688478537266425`, −0.0033368879400606`, −0.3600422628858915`},
        {−0.20016743406321802`, −0.141786776016792`, −0.0031833132819370606`, −0.025035128263311796`},
        {−1.0370232050448622`, −0.12104819573424286`, −0.00279162773246354`, 0.93734417935672`},
        {−1.6206748205875385`, −0.12175843659287935`, −0.0010603060335673674`, 1.5727819146856916`},
        {−2.3140017414648457`, −0.1507019059111019`, −0.00013515465834348776`, 2.599135932824302`},
        {−0.23103769681839986`, −0.21205417781294456`, 0.0022778517579115677`, 0.022317410331553864`},
        {5.620180481722192`, −0.37988679738646947`, −0.01692804180806394`, −3.9571335857250705`},
        {−0.6394502196965849`, −0.11177937726524836`, 0.00505350057157611`, −3.595608204152356`},
        {−5.423116075440216`, −0.105828558814025`, 0.0016466288545533623`, 2.5125629233840594`},
        {2.983757016972453`, −0.18292506151915733`, 0.010783776603821139`, −6.9515331648547445`},
        {7.4939526560863285`, −0.19654243508265964`, 0.010992765804029832`, −11.575048133647416`},
        {7.090897657494067`, −0.15825523089389787`, 0.008367200700772327`, −10.863848556697198`}};
Es11 = {{4.451997215578638`, −0.09474255645606608`, −0.012775724741904724`, −6.193918322137051`},
        {3.957973472473779`, −0.084289084268172`, −0.01344459036891392`, −5.5774472677143825`},
        {3.0655074661164665`, −0.07445598289971554`, −0.01453032402675125`, −4.3865727131861485`},
        {2.7907497662045757`, −0.0849791558042531`, −0.011350630447806225`, −4.071564595175762`},
        {2.9379541470165895`, −0.10438394484995689`, −0.00914217572234629`, −4.133154510443281`},
        {0.47987305299196353`, −0.22733819613306952`, −0.015000617701155461`, 1.25780834036857`},
        {5.894412477880864`, −0.3805736476532139`, −0.011887356495557236`, −4.428034488934852`},
        {−3.2556510956926226`, −0.09418890296872945`, 0.002400680544390636`, 0.09972134042397435`},
        {−9.854027530892957`, −0.17108771621062685`, 0.0031028004461154906`, 9.931322779900666`},
        {−1.3065390737613038`, −0.1738569292491596`, −0.00371008354130536`, 1.2715241365166425`},
        {0.0924614592893833`, −0.16829120246077145`, −0.0010431855959568844`, −0.5527673007348359`},
        {0.3980859502475215`, −0.151375706390227`, −0.002646985875974652`, −0.7976765483673897`},
        {0.10629071964791194`, −0.14634126114930335`, −0.002507402509479418`, −0.476854663941709`},
        {−0.7351559944712951`, −0.12472151195073634`, −0.0022224071277419043`, 0.49900111911265527`},
        {−1.6139998499797072`, −0.12187604584268359`, 0.0000636137199646810 1`, 1.4351148136075338`},
        {−2.5778968564545215`, −0.14455235404334885`, 0.0015508528683109185`, 2.7229110328685717`},
        {−0.20699253422157532`, −0.20904352204134327`, 0.004278399982170455`, −0.26860550793165566`},
        {6.017035186881453`, −0.36064396296498874`, −0.01759278123792813`, −4.736280152604597`},
        {−2.02162304441456`, −0.08961446968087905`, 0.006493467338458072`, −2.5249175129402497`},
        {−5.122206620736595`, −0.11532169979666868`, 0.0011942870548145288`, 2.2759606636926883`},
        {3.1763398663810904`, −0.18172068732386054`, 0.011681691162432443`, −7.265334049998894`},
        {7.219122363363802`, −0.18803436697914028`, 0.011134660871288992`, −11.31440300631971`},
        {7.295687154723665`, −0.1555664766946561`, 0.008952833205172586`, −11.24636742907698`}};
Es12 = {{3.659054904508988`, −0.09435977806591872`, −0.012854450158956025`, −5.126465365874477`},
        {3.041676243025657`, −0.0790619208478258`, −0.013620533866869497`, −4.39267403849669`},
        {1.8712445542715548`, −0.06302214738541635`, −0.014303474237766881`, −2.9339252493449606`},
        {1.8537474901632767`, −0.08234828844023295`, −0.010379370717016851`, −2.9272150399254495`},
        {1.7840441409374534`, −0.1008210294139387`, −0.008197989135117687`, −2.7031475656901782`},
        {−0.7697393661352997`, −0.27032763351912914`, −0.014732329835294756`, 3.4860575190272995`},
        {4.9649550335860075`, −0.39971558343091523`, −0.013392540447759834`, −2.705997068377896`},
        {−5.545140460284527`, −0.06736074290752608`, 0.0011608556223925714`, 2.8634036726617538`},
        {−12.609672677607184`, −0.1845792966034557`, 0.0012080039222449844`, 13.297686243894562`},
        {−2.921693526720529`, −0.21224137643090762`, −0.0036445057005906304`, 3.349295389372551`},
        {−0.3673174425129984`, −0.2138269798588859`, −0.0021805333604986944`, 0.3432005693887031`},
        {−0.017530139032844 24`, −0.1907816676844511`, −0.003852269811081132`, 0.03955709027845237`},
        {−0.2999633187261043`, −0.18578127316733653`, −0.0036829279692549366`, 0.3422649794201706`},
        {−1.1362986255830632`, −0.15972342863429503`, −0.003410415240463311 6`, 1.28304171248883`},
        {−1.568948920283904`, −0.15721516769550467`, −0.002218914219823324`, 1.7665791028896067`},
        {−2.15816921143171`, −0.18522544452527553`, −0.0019834340333311825`, 2.717121985028097`},
        {−1.428649710052351`, −0.2501280405074496`, −0.0010204342494863111`, 2.186722697418564`},
        {3.9789730235662035`, −0.4265066999317244`, −0.020536531552467097`, −0.8932346301122189`},
```

```
      {-1.4545099157730434`, -0.1251169946299162`, 0.002539835219040143`, -2.1029775802589823`},
      {-6.86524725045058`, -0.10625576917334298`, 0.0007326726008042345`, 4.347009851731982`},
      {2.5843910612481924`, -0.20074654667318656`, 0.01015985080856245`, -6.282440259625149`},
      {9.629547827287416`, -0.23251059475070823`, 0.01268148844733489`, -14.127357844939459`},
      {7.6484839160066524`, -0.19063069286653014`, 0.008968448945700026`, -11.371697126746408`}};
Es13 = {{4.277452396908964`, -0.07870996363617122`, -0.011753385580270951`, -6.132112243220072`},
      {3.8219945241719255`, -0.07091319600160108`, -0.012477305573581189`, -5.535796522387685`},
      {2.8724782329092706`, -0.06130682499184318`, -0.014710777418835799`, -4.184637558826297`},
      {2.514125492761242`, -0.06783478436866448`, -0.012244420245462571`, -3.7417644154209277`},
      {2.9355471504133397`, -0.08976266824467427`, -0.009998650376708499`, -4.15268174029144`},
      {1.3535645268997032`, -0.17776138227815177`, -0.015323440914563991`, -0.5358103474038077`},
      {6.618223025126479`, -0.33786821186061455`, -0.008756220805321298`, -6.3357316520874996`},
      {-1.6810472004502035`, -0.10302911498801523`, 0.0013987929171358183`, -1.5459878880010134`},
      {-8.793459844405357`, -0.14892270302434638`, 0.004934663440169621`, 8.45024171362177`},
      {-0.6444154940850709`, -0.14026531110056514`, -0.002727368557523183`, 0.2775320618964992`},
      {0.062097713571521045`, -0.1287234229869739`, 0.00030366498223059697`, -0.7724358876173094`},
      {0.4112076205834367`, -0.11196392660842858`, -0.00121811955057593`, -1.0680642297619307`},
      {0.11417657975834898`, -0.10648525070428348`, -0.0011958609596571517`, -0.7255149150579338`},
      {-0.7166071043091872`, -0.08825352327282626`, -0.0009591953096396368`, 0.26277628828081506`},
      {-1.6163320576208378`, -0.0880357465684511`, 0.0010848480025120568`, 1.2570533435626152`},
      {-2.28120273971899`, -0.11225082855633418`, 0.002332066577854049`, 2.195688416624886`},
      {1.2287790921117419`, -0.171596519595238`, 0.005571601707305694`, -2.4711843652558225`},
      {8.864220467308396`, -0.29463994196729426`, -0.011616738016042466`, -9.95744767725282`},
      {1.7409304478759173`, -0.09969976865521178`, 0.008714631968673234`, -7.260706107582489`},
      {-3.111423316623149`, -0.1126222515439441`, 0.0027273700824481176`, -0.3445845057683117`},
      {3.3517230178811515`, -0.16220887398766898`, 0.01172330132340106`, -7.596973539019283`},
      {5.941442298627067`, -0.16094535632138612`, 0.009499994837497187`, -9.694176162594722`},
      {6.738799498916517`, -0.12746801164957813`, 0.007484497268149622`, -10.54728235298692`}};
Es14 = {{4.138381206262848`, -0.08329582614621125`, -0.012543499711344486`, -5.870798644383503`},
      {3.6638821731844016`, -0.07395330557359667`, -0.013217823623583015`, -5.267502873922596`},
      {2.731465133942441`, -0.06396063028006166`, -0.014804480751073476`, -3.988292460046113`},
      {2.4565663678987755`, -0.07338981205249165`, -0.011934562657877675`, -3.6619753208459764`},
      {2.721673610409863`, -0.09391976241348192`, -0.009742224565706826`, -3.8702311445658903`},
      {0.626464148141365`, -0.20353820468112652`, -0.015183517602088251`, 0.7870904174928377`},
      {5.991871959373379`, -0.36013280629617267`, -0.010417792636657736`, -4.993955522832422`},
      {-2.773528154420251`, -0.09526333769440026`, 0.0017360928484562933`, -0.32267514426823074`},
      {-9.470786004881175`, -0.1573161889005528`, 0.0037102328154868763`, 9.36897630959587`},
      {-1.1622535656705761`, -0.15579215088079804`, -0.00324508406848114`, 0.9919864411721122`},
      {0.009990994097157244`, -0.14828017255075357`, -0.0006880444428942767`, -0.5524247650852017`},
      {0.33666209644989203`, -0.13097339456393978`, -0.0022691418391822048`, -0.8198679855299729`},
      {0.03524422783128669`, -0.1256663483766611`, -0.002165854651033776`, -0.48240584400366493`},
      {-0.8044438112932615`, -0.10605618298425255`, -0.0018478927774367472`, 0.4998722088968928`},
      {-1.6271698846561857`, -0.1054570909173611`, 0.00025253121585043324`, 1.3925845034457336`},
      {-2.4116395646371713`, -0.13061561426045692`, 0.001540555390386067`, 2.483933226973901`},
      {0.4691378621967362`, -0.1918168313085718`, 0.004484372177957427`, -1.2599425835340046`},
      {7.031820110600259`, -0.3362734315437565`, -0.014822657757354`, -6.629638993499176`},
      {-0.3190434219648153`, -0.09670626220461315`, 0.007126837309771833`, -4.534836573123322`},
      {-4.34419733943491`, -0.11103387770406989`, 0.0020198539271568624`, 1.212488738461437`},
      {3.2285259529711827`, -0.1727625085361578`, 0.011469225437278139`, -7.368943051866102`},
      {6.597941675969096`, -0.17669928962329381`, 0.010304491952853868`, -10.518897262207954`},
      {6.9565722592029395`, -0.1423176721087122`, 0.008153294116658698`, -10.8042318265186`}};
Es15 = {{4.871933683251892`, -0.10557794075922744`, -0.012793314366481037`, -6.673285381795246`},
      {4.364169114425135`, -0.09458373318261443`, -0.013465273643108604`, -6.045386138137347`},
      {3.511719435460646`, -0.08509730989776013`, -0.014272654698653792`, -4.925232504557205`},
      {3.2086205611740652`, -0.09560490798863781`, -0.010947884061183306`, -4.582140826005783`},
```

{3.2892055306319383`, −0.11425614290258934`, −0.008717565891974137`, −4.564585728638897`},
{0.5748040952224408`, −0.24245017250668968`, −0.01485677809255876`, 1.2885097442963387`},
{6.004226946954772`, −0.39440361241205435`, −0.012760077721361854`, −4.303512401111277`},
{−3.18760399690593`, −0.09822092778101173`, 0.0032329794688152422`, −0.12098231984720519`},
{−9.968665585333143`, −0.18321639560240546`, 0.002823310687159845`, 10.152480651652748`},
{−1.2249163217488057`, −0.1871368708600577`, −0.004065093845500047`, 1.2564591698276781`},
{0.2303564258190973`, −0.18181680215745874`, −0.0011025643906030004`, −0.6710067907344717`},
{0.505892124675624`, −0.16571594616311747`, −0.0026919367047675237`, −0.8844480771638737`},
{0.2247166099861705`, −0.16091568822046887`, −0.0025419631247805097`, −0.5780284367160318`},
{−0.6158429838708901`, −0.13763471810828487`, −0.002316292264464739`, 0.39263948018130906`},
{−1.5941388778683852`, −0.1326230347362031`, 0.00024691774900026565`, 1.4159700579569559`},
{−2.747242016776114`, −0.15201121435689047`, 0.0019971095617041103`, 2.900852042261823`},
{−0.6472618274352566`, −0.2192190339954596`, 0.004604698192500462`, 0.31498496732488207`},
{5.547814677136509`, −0.37078250813814895`, −0.019300523528023836`, −3.8604812654821328`},
{−3.265786062639064`, −0.08077542673362632`, 0.0065373488548621005`, −1.1984980594570493`},
{−5.52285207481126`, −0.11996521009100618`, 0.0005108977097471731`, 2.8705254881451925`},
{3.1978107545952548`, −0.18753831856375378`, 0.012114393305163784`, −7.292965039230147`},
{7.65442679384202`, −0.194273172543634`, 0.011794816586355778`, −11.88920538420945`},
{7.61007513650415`, −0.16381869782503183`, 0.00969789789478836`, −11.686280406285666`}};
Es16 = {{2.6537293597754132`, −0.08405012388058358`, −0.01265490330308521`, −3.8429863213671385`},
{1.9267700990058503`, −0.06487927991696521`, −0.013455197687581317`, −3.008938420997548`},
{0.2594673141703837`, −0.038665559348986606`, −0.014362911916414105`, −1.006839011032996`},
{0.7655916903687497`, −0.07135370675321187`, −0.009951478834734285`, −1.5917205232941183`},
{0.8395921219438163`, −0.09501275722399405`, −0.0077694086268438`, −1.5044826153157296`},
{−1.0768025008700066`, −0.2974993818334848`, −0.014666614583121199`, 4.228365203779776`},
{4.648095584289175`, −0.3963246845124197`, −0.013543126803550892`, −2.259212736686033`},
{−5.964167518856695`, −0.06180169506367358`, −0.0005650371901972656`, 3.686774768452133`},
{−15.344010373974475`, −0.1909813960417325`, −0.00017084007887729024`, 16.559022805220454`},
{−3.6466700439093955`, −0.2520046804478411`, −0.003135223965534721`, 4.393577019920957`},
{−0.383591351796607`, −0.262147567438565`, −0.0017821014030742388`, 0.6340164281452331`},
{0.036059848472686416`, −0.22959471831234568`, −0.0034787252478122753`, 0.22860843221768787`},
{−0.2531749004874855`, −0.22365414673651074`, −0.0033372749631923183`, 0.5360177608665195`},
{−1.135106341499165`, −0.19353521837722842`, −0.003029303083582734`, 1.4991043860662148`},
{−1.417213070361462`, −0.18929787510697235`, −0.002421206271080489`, 1.8372801976476998`},
{−1.8172246727978463`, −0.2166208627834375`, −0.0043174491137676334`, 2.734455086057325`},
{−1.8789985749465452`, −0.2805935496453627`, −0.006712677132811111`, 3.6220787498554223`},
{3.201242689498095`, −0.4526758094641757`, −0.021003234038914853`, 0.48845638681928505`},
{0.6741917401225646`, −0.15840396243944288`, 0.0013050163322183925`, −3.9651538112490305`},
{−6.950580473740632`, −0.10307147069482571`, 0.001172245800416585`, 4.376610453825008`},
{2.0733655421357025`, −0.21329897470123022`, 0.009056130464528656`, −5.475924369046228`},
{12.916152382053115`, −0.2793974009840758`, 0.014433144609732095`, −17.94586493920608`},
{7.956357615949928`, −0.22105856039052665`, 0.008056423600936096`, −11.30223329031789`}};
Es17 = {{−5.019121719597795`, 0.024772172342692085`, 0.003080729651082842`, 3.3817948583881323`},
{−6.365590601998595`, 0.002369288338906992`, −0.00001925329262730408`, 6.200000424618344`},
{1.0220161804990782`, −0.10279107190513588`, 0.0036200542544557266`, −0.9518369990417137`},
{1.2853214280736283`, −0.1055571210593467`, 0.003966499404433437`, −1.2326296812385544`},
{1.1352079684207543`, −0.12277922143540652`, 0.0030117573961268265`, −0.18036347908963674`},
{1.1390549914400119`, −0.10795613246330303`, 0.004032099663799631`, −0.9295427033882285`},
{−0.8801788906890027`, −0.03591701363902229`, 0.001999009172599853`, −0.2091980815155924`},
{−0.6783464508494848`, 0.016847370239231024`, 0.00021718727240227072`, −1.6579194461311844`},
{−4.7562051203299545`, 0.10094542123220192`, −0.0007116664794906097`, 1.1862203444129202`},
{−5.648040802241088`, 0.09745642105848064`, −0.0004817496255743754`, 2.3539575022267063`},
{−5.492245694984299`, 0.09386741057109027`, 0.00019743155386386452`, 2.1151039281429744`},
{−3.6045160417066024`, 0.045145606804957196`, 0.0006765986894854457`, 1.0705118751802756`},
{−1.4771623673036813`, 0.007925624272250048`, 0.0014773356689492642`, −0.6934254912213276`},

```
        {1.5566939181198591`, −0.02297912625165834`, 0.0031754259124747077`, −3.944017109243573`},
        {1.9168551283512536`, −0.015537387729474918`, 0.0033073445900267443`, −4.862898054405561`},
        {0.5892951784729359`, 0.04738784051628985`, 0.0029748102294755354`, −5.467193402437419`},
        {−0.6344256692271798`, 0.06512519171135023`, 0.0024576384281917198`, −4.369235810082927`},
        {1.7900466439785383`, −0.005627630831561378`, 0.0023353720380426043`, −4.820821261867021`},
        {2.0743645038633605`, 0.0336832940463449`, 0.00019834671303136444`, −5.703536020416088`},
        {−0.3994338138365181`, 0.048055896379218775`, −0.0002454332207737434`, −2.8190874804514463`},
        {−2.9591320525211318`, 0.03542308247265564`, −0.00127465857142252`, 1.1409589877272455`},
        {−2.957585464813942`, 0.047790521597396024`, −0.001479960376662401`, 0.8621030632507286`},
        {−1.910094636977632`, 0.05970185583241539`, −0.0004968654438869866`, −0.9850687061242013`}};
Es18 = {{−5.968403483877015`, 0.02206400563733288`, 0.0031499173261994758`, 4.750595912862819`},
        {−6.174703451557826`, −0.004813387901627558`, 0.00020295429283041008`, 6.165354709036221`},
        {3.3331912869175437`, −0.12068104392186184`, 0.004577026658645695`, −3.6084782415833745`},
        {2.907392767738971`, −0.1169019070000187`, 0.004706507652491154`, −3.155432956936419`},
        {0.9827258141891375`, −0.11684131330614266`, 0.0028700335906850405`, −0.14921163063777326`},
        {−0.28226869703833407`, −0.09887659826196324`, 0.003186992211012681`, 0.8600653706122543`},
        {−1.4072061558656312`, −0.059056647331247325`, 0.0014746415229821377`, 1.469073466772488`},
        {−1.3286784142249104`, −0.003810918248563614`, 0.00008564998260373866`, −0.044059482663096644`},
        {−5.349870601757787`, 0.08831969140452865`, −0.0008720305930476367`, 2.4342228576035203`},
        {−6.345918462334507`, 0.08521510269381387`, −0.0007225559325040918`, 3.7560987484779833`},
        {−6.525863356773333`, 0.08764759948697061`, −0.0001456996591723609 8`, 3.797588801422541`},
        {−4.632592907098865`, 0.046273472081327455`, 0.0003476622618922853 6`, 2.4928730971750857`},
        {−1.9509229328378248`, 0.006301762619873766`, 0.0011485101208420477`, 0.05673035511863669`},
        {1.699312990123306`, −0.02516939439201328`, 0.002951478302334713`, −4.03639662682356`},
        {2.953020188417279`, −0.023150146198798963`, 0.00349481630825714 5`, −6.03399168358611`},
        {1.324417939918378`, 0.036429164577222305`, 0.002981437010875719`, −6.056827986934563`},
        {0.5149294139554875`, 0.04399774581263784`, 0.002424726146948818`, −5.119584448904645`},
        {3.0353004249694044`, −0.03669115649980367`, 0.0018650881188654923`, −5.232756382063689`},
        {3.3913426830254547`, 0.019165789131700077`, −0.00017788668762940975`, −6.832370307232855`},
        {0.5949013045286885`, 0.041299871775683825`, −0.0008942866917220193`, −3.7154579011933153`},
        {−2.3177500452784088`, 0.029115845453717776`, −0.001400934295884264`, 0.5194165201837867`},
        {−2.5167934276426926`, 0.03746160166475701`, −0.001639747894683668`, 0.6168699760362695`},
        {−1.5057573106985456`, 0.0500024381320744`, −0.0007284724404940961`, −1.1964143479512417`}};
Es19 = {{−6.973291008879074`, 0.029612960937967638`, 0.002798534291054635`, 5.92581449913463`},
        {−7.7451021231519155`, 0.004973627507715593`, −0.00017992020561087349`, 8.034956289099911`},
        {0.8210833991189236`, −0.10813580412200603`, 0.0035939302600825618`, −0.47106383310698663`},
        {0.5929297230880414`, −0.1044560633199339`, 0.003770086026898858`, −0.2877345442052154`},
        {−0.25871514405957347`, −0.11778291392829995`, 0.002390497908405656`, 1.665363533368099`},
        {−0.8258853443349135`, −0.10826261088954173`, 0.0032216285988051663`, 1.9127773661368017`},
        {−1.5530574481889552`, −0.09237162924172103`, 0.001480512082179504`, 2.846280630677839`},
        {−1.7515427395167027`, −0.031558953605926816`, 0.0000942207605938104`, 1.476207514203275`},
        {−5.975391521451722`, 0.08358045605500364`, −0.0009421008955015548`, 3.4728396252576745`},
        {−6.808371925388668`, 0.08586170813212907`, −0.0008305158852621517`, 4.4303768447456635`},
        {−7.060992769538313`, 0.08724821283738539`, −0.00030188429419515607`, 4.611528215251013`},
        {−5.365989562901412`, 0.04296527099102351`, 0.00014274352859011547`, 3.691751350444461`},
        {−2.345567639474604`, −0.00830912499449923`, 0.0010632766710766178`, 1.1551091765407249`},
        {0.5589193964903251`, −0.03666394021032607`, 0.002240161872032003`, −1.972611140449185`},
        {1.5574742017528316`, −0.034568838168476876`, 0.0024758818863727192`, −3.571819659227404`},
        {0.8236087251148828`, 0.014465149140698342`, 0.002512637984448265`, −4.532690125857384`},
        {−0.3269184412485554`, 0.024627604050726754`, 0.001975451774926682`, −3.268169203750694`},
        {1.8297409507167506`, −0.045083205934217954`, 0.0018680983462894282`, −3.4347063691083743`},
        {2.0290965507083576`, 0.002041178116627876`, 0.00005589864665924219`, −4.592617739316233`},
        {−0.9607355321493912`, 0.03961869640551629`, −0.0006830252816315433`, −1.7535313772858372`},
        {−3.5913652726156244`, 0.027635401895998373`, −0.0015794525341513246`, 2.243332276099856`},
        {−3.32862651358546`, 0.04927079904031526`, −0.0018578006875273093`, 1.3744054108441208`},
```

```
   {−2.2181462323085377`, 0.06505616712110508`, −0.0008025653730298377`, −0.6893495709011095`}};
Es20 = {{−8.571052210119332`, 0.12983996876762782`, 0.0007955927124505584`, 4.883119727692465`},
   {−8.91702555269611`, 0.0818873829551222`, −0.0006431703931035728`, 7.004767769632494`},
   {−5.8352302736551485`, −0.0029620658433763287`, 0.000756734942607326`, 5.29953247628682`},
   {−5.465930826186013`, 0.006264408191687319`, 0.0010538611289741804`, 4.432116099676048`},
   {−2.1768346278261017`, −0.04369836288000274`, 0.002185895519003179 6`, 1.613392924469569`},
   {1.1331666583004494`, −0.025744750428105`, 0.006006044503053832`, −4.4143569029033705`},
   {−5.6965539397955265`, 0.1864238204286457`, 0.0031790332825644718`, −2.228534578428679`},
   {−2.1600780169611054`, 0.1358398839819075`, −0.0003181952286018502`, −3.7519423346332026`},
   {−5.884002490888868`, 0.194741097973631`, 0.00030036506329120244`, −0.7804152085815941`},
   {−6.45315094060076`, 0.20467620313264176`, 0.0006535351511033569`, −0.506336784166596`},
   {−5.983607502853691`, 0.193690370577835`, 0.0013516349395335885`, −0.9303517366453808`},
   {−3.5455215915652047`, 0.10998132016348933`, 0.001958129741002991`, −1.4981696108309281`},
   {−1.9556638027841489`, 0.07171367301253978`, 0.00278858864286538`, −2.4776453251301036`},
   {−0.8902286114155044`, 0.05609847724085163`, 0.0035740929482352313`, −3.4727803740883347`},
   {−2.9408367245629`, 0.08888556228882245`, 0.0027233477621261574`, −1.9601245600019983`},
   {−8.265675447185824`, 0.2903715203906545`, 0.002661612500247019`, −2.492499964727485`},
   {−12.550453088796258`, 0.38868429623075035`, 0.002963616726839581`, −0.5860426063826001`},
   {−10.270570803922396`, 0.34314838972156986`, 0.00489849647225675`, −2.240346792688579`},
   {−6.109293070094556`, 0.2630536045508453`, 0.002961338686692385`, −3.8006598552438113`},
   {−3.8939555954130736`, 0.154741948779493`, 0.002885972269117779`, −2.7327764757012116`},
   {−5.138551685906231`, 0.10589067499393054`, −0.0005561857418745656`, 1.586716992017165`},
   {−4.893439726186266`, 0.14090051960178052`, −0.0007048576696723328`, 0.33429744417384194`},
   {−3.749049870376697`, 0.13708900183739453`, 0.00040277774268004094`, −1.1611788024486631`}};
Es21 = {{−4.309899060230262`, 0.01503223983576925`, 0.0034516015139100602`, 2.6888586807515846`},
   {−5.191146631476554`, −0.008277725936715871`, 0.0003059795151474762`, 4.917861794719721`},
   {3.4755889073588553`, −0.11945193979011895`, 0.0046133426413245224`, −3.855073854751909`},
   {3.41556922839607`, −0.12068655275304022`, 0.004859902654483811`, −3.741277305575603`},
   {2.032538361487651`, −0.12429796049408041`, 0.0033053511752958167`, −1.3975438832640854`},
   {1.168737376528506`, −0.10365982510953921`, 0.003742005587233266`, −1.0478046331983442`},
   {−0.7492502777892909`, −0.03161967802228992`, 0.001816402635027373`, −0.4830431580410579`},
   {−0.5412470225037082`, 0.019992604193499408`, 0.000208971257197227`, −1.9512017682444698`},
   {−4.410397420461803`, 0.09602367008523836`, −0.0007245950903331834`, 0.8770245658663505`},
   {−5.425418692206539`, 0.08971814649697699`, −0.0004937834603506429`, 2.291101954306322`},
   {−5.316975821058165`, 0.08847246765042963`, 0.0001771931218882015`, 2.042941150843896`},
   {−3.3282854989923494`, 0.043971211468836806`, 0.0006732138319354553`, 0.7098973886593725`},
   {−1.268521805211079`, 0.01322797505366905`, 0.001398656999984533`, −1.166426396646303`},
   {2.386900252373767`, −0.019891459192490437`, 0.003499767038384131`, −5.229638275865717`},
   {3.149675805185863`, −0.014199653934441791`, 0.003908333358288664`, −6.677203060606489`},
   {1.3115450642508786`, 0.05127144492443647`, 0.003226885647163892`, −6.603192998734945`},
   {0.44701923185116405`, 0.06302219704266278`, 0.0026643467144346444`, −5.735560476811174`},
   {3.0877631938285557`, −0.017141655848839657`, 0.0021576262057638996`, −6.029776227727726`},
   {3.4006847657865276`, 0.03261616690082558`, −0.00012136507486207066`, −7.284086980149651`},
   {0.8697553638493647`, 0.04368466149704865`, −0.0006455548952952704`, −4.18782719636406`},
   {−1.946058072016888`, 0.031828774909355845`, −0.0012237308281768308`, −0.0712573671151994`},
   {−2.224295338223753`, 0.0362052027914455`, −0.0013996688189846659`, 0.22624066276427204`},
   {−1.2637523933103358`, 0.046332386855476033`, −0.0005274163866649316`, −1.438825665041676`}};
Es22 = {{−3.997786123134445`, 0.11039916811847628`, 0.0019917426336525015`, −0.8224976029666095`},
   {−4.7634343942654045`, 0.060111670121858495`, −0.000010305712534249804`, 1.999129579464737`},
   {−1.2194502344239293`, −0.018357996970991733`, 0.0018393574257492631`, −0.602610764271614`},
   {−0.6833029617369885`, −0.010385967391941506`, 0.00231534517044146 5`, −1.678609970574118`},
   {2.6653077050609975`, −0.06597258904996056`, 0.0037786156959906426`, −4.479529198458175`},
   {6.201736942953636`, −0.07126677897513951`, 0.007496503525012565`, −9.949509019648941`},
   {−3.6736373352084652`, 0.23511331848097786`, 0.003812554508742388`, −6.880856072644585`},
   {−0.2553146056048227`, 0.18920530441763164`, 0.00030050423571314876`, −8.369411019690434`},
```

```
{-3.3791558241288295`, 0.21688308656753139`, 0.00033566601766804165`, -4.942846566268658`},
{-3.94916618301819`, 0.216456094268072`, 0.0010009021756775158`, -4.442651171066261`},
{-3.1988409710485852`, 0.20378615244930662`, 0.001926504068500745`, -5.2295412116700986`},
{-0.9046305362573286`, 0.12593164728684034`, 0.002585206025511056`, -5.840687643383652`},
{0.4355389590326045`, 0.10606418802322451`, 0.0035042524364625213`, -7.104213000030648`},
{2.598295664538044`, 0.07947533841418712`, 0.00504465922347779`, -9.27412987401785`},
{0.16891273411552435`, 0.12335552908379044`, 0.004595531877082297`, -7.736396780143838`},
{-5.580048938331185`, 0.3246393193595847`, 0.0041001229937584985`, -7.579647107526696`},
{-9.462648664668567`, 0.42072945591414934`, 0.00439013686851294`, -6.041595435271445`},
{-6.692951814087483`, 0.3656089523580108`, 0.005783934027116653`, -7.823611075032799`},
{-2.62051656266946`, 0.28882096303498234`, 0.0034130421046365293`, -9.283867558141914`},
{0.44041204709435106`, 0.14914065645287436`, 0.003675770072216563`, -8.361721343387105`},
{-1.4048813702996714`, 0.1081778869845482`, -0.00019089467660534867`, -3.4178469553186552`},
{-1.6094051797216509`, 0.1303055515973871`, -0.0002352146253466577`, -3.7805435564877965`},
{-0.5751591894605862`, 0.11534409582823091`, 0.0009090295646993716`, -4.818900882849865`}};
```

We can draw the graph to see how does the coefficient of these 22 countries change from Jan-2012 to Dec-2013

```
(*For temperature*)
Plot[{RefineEs[x, 2, 4, {1, 2, 3, 4, 5, 6, 7, 8, 9, 11, 12, 13, 14, 15, 16, 17, 18, 19, 20, 21, 22, 23, 24}, Es1][[2]],
   RefineEs[x, 2, 4, {1, 2, 3, 4, 5, 6, 7, 8, 9, 11, 12, 13, 14, 15, 16, 17, 18, 19, 20, 21, 22, 23, 24}, Es2][[2]],
   RefineEs[x, 2, 4, {1, 2, 3, 4, 5, 6, 7, 8, 9, 11, 12, 13, 14, 15, 16, 17, 18, 19, 20, 21, 22, 23, 24}, Es3][[2]],
   RefineEs[x, 2, 4, {1, 2, 3, 4, 5, 6, 7, 8, 9, 11, 12, 13, 14, 15, 16, 17, 18, 19, 20, 21, 22, 23, 24}, Es4][[2]],
   RefineEs[x, 2, 4, {1, 2, 3, 4, 5, 6, 7, 8, 9, 11, 12, 13, 14, 15, 16, 17, 18, 19, 20, 21, 22, 23, 24}, Es5][[2]],
   RefineEs[x, 2, 4, {1, 2, 3, 4, 5, 6, 7, 8, 9, 11, 12, 13, 14, 15, 16, 17, 18, 19, 20, 21, 22, 23, 24}, Es6][[2]],
   RefineEs[x, 2, 4, {1, 2, 3, 4, 5, 6, 7, 8, 9, 11, 12, 13, 14, 15, 16, 17, 18, 19, 20, 21, 22, 23, 24}, Es7][[2]],
   RefineEs[x, 2, 4, {1, 2, 3, 4, 5, 6, 7, 8, 9, 11, 12, 13, 14, 15, 16, 17, 18, 19, 20, 21, 22, 23, 24}, Es8][[2]],
   RefineEs[x, 2, 4, {1, 2, 3, 4, 5, 6, 7, 8, 9, 11, 12, 13, 14, 15, 16, 17, 18, 19, 20, 21, 22, 23, 24}, Es9][[2]],
   RefineEs[x, 2, 4, {1, 2, 3, 4, 5, 6, 7, 8, 9, 11, 12, 13, 14, 15, 16, 17, 18, 19, 20, 21, 22, 23, 24}, Es10][[2]],
   RefineEs[x, 2, 4, {1, 2, 3, 4, 5, 6, 7, 8, 9, 11, 12, 13, 14, 15, 16, 17, 18, 19, 20, 21, 22, 23, 24}, Es11][[2]],
   RefineEs[x, 2, 4, {1, 2, 3, 4, 5, 6, 7, 8, 9, 11, 12, 13, 14, 15, 16, 17, 18, 19, 20, 21, 22, 23, 24}, Es12][[2]],
   RefineEs[x, 2, 4, {1, 2, 3, 4, 5, 6, 7, 8, 9, 11, 12, 13, 14, 15, 16, 17, 18, 19, 20, 21, 22, 23, 24}, Es13][[2]],
   RefineEs[x, 2, 4, {1, 2, 3, 4, 5, 6, 7, 8, 9, 11, 12, 13, 14, 15, 16, 17, 18, 19, 20, 21, 22, 23, 24}, Es14][[2]],
   RefineEs[x, 2, 4, {1, 2, 3, 4, 5, 6, 7, 8, 9, 11, 12, 13, 14, 15, 16, 17, 18, 19, 20, 21, 22, 23, 24}, Es15][[2]],
   RefineEs[x, 2, 4, {1, 2, 3, 4, 5, 6, 7, 8, 9, 11, 12, 13, 14, 15, 16, 17, 18, 19, 20, 21, 22, 23, 24}, Es16][[2]],
   RefineEs[x, 2, 3, {1, 2, 3, 4, 5, 6, 7, 8, 9, 11, 12, 13, 14, 15, 16, 17, 18, 19, 20, 21, 22, 23, 24}, Es17][[2]],
   RefineEs[x, 2, 3, {1, 2, 3, 4, 5, 6, 7, 8, 9, 11, 12, 13, 14, 15, 16, 17, 18, 19, 20, 21, 22, 23, 24}, Es18][[2]],
   RefineEs[x, 2, 3, {1, 2, 3, 4, 5, 6, 7, 8, 9, 11, 12, 13, 14, 15, 16, 17, 18, 19, 20, 21, 22, 23, 24}, Es19][[2]],
   RefineEs[x, 2, 3, {1, 2, 3, 4, 5, 6, 7, 8, 9, 11, 12, 13, 14, 15, 16, 17, 18, 19, 20, 21, 22, 23, 24}, Es20][[2]],
   RefineEs[x, 2, 3, {1, 2, 3, 4, 5, 6, 7, 8, 9, 11, 12, 13, 14, 15, 16, 17, 18, 19, 20, 21, 22, 23, 24}, Es21][[2]],
   RefineEs[x, 2, 3, {1, 2, 3, 4, 5, 6, 7, 8, 9, 11, 12, 13, 14, 15, 16, 17, 18, 19, 20, 21, 22, 23, 24}, Es22][[2]]},
  {x, 1, 24}, AxesLabel → {"Month Since 2012", Beta}, ImageSize → Large,
  Exclusions → {1, 2, 3, 4, 5, 6, 7, 8, 9, 10, 11, 12, 13, 14, 15, 16, 17, 18, 19, 20, 21, 22, 23, 24},
  PlotLabels → {"Slovenia", "Slovakia", "Poland", "Austria", "Belgium", "Bosnia and Herzegovina",
     "Germany", "France", "Netherlands", "Czech Republic", "Croatia", "Luxembourg", "Romania", "Hungary",
     "Italy", "United Kingdom", "Cambodia", "Laos", "Thailand", "Singapore", "Vietnam", "Malaysia"},
  PlotLegends → {"Slovenia", "Slovakia", "Poland", "Austria", "Belgium", "Bosnia and Herzegovina", "Germany",
     "France", "Netherlands", "Czech Republic", "Croatia", "Luxembourg", "Romania", "Hungary",
     "Italy", "United Kingdom", "Cambodia", "Laos", "Thailand", "Singapore", "Vietnam", "Malaysia"},
  PlotStyle → {ColorData["Rainbow"][0.04], ColorData["Rainbow"][0.09], ColorData["Rainbow"][0.14],
     ColorData["Rainbow"][0.19], ColorData["Rainbow"][0.24], ColorData["Rainbow"][0.29],
     ColorData["Rainbow"][0.34], ColorData["Rainbow"][0.39], ColorData["Rainbow"][0.44], ColorData["Rainbow"][0.49],
     ColorData["Rainbow"][0.54], ColorData["Rainbow"][0.59], ColorData["Rainbow"][0.64], ColorData["Rainbow"][0.69],
     ColorData["Rainbow"][0.74], ColorData["Rainbow"][0.79], ColorData["Rainbow"][0.84], ColorData["Rainbow"][0.89],
     ColorData["Rainbow"][0.76], ColorData["Rainbow"][0.8], ColorData["Rainbow"][0.94], ColorData["Rainbow"][0.99]}]
```

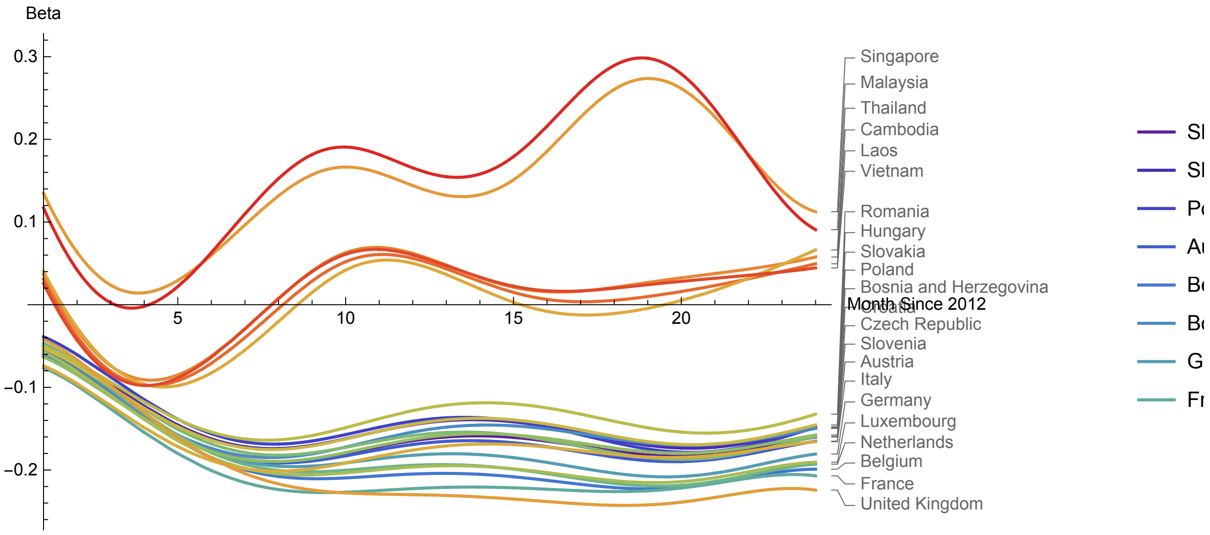
Beta
0.3
0.2
0.1
−0.1
−0.2
5
10
15
20
Month Since 2012
Singapore
Malaysia
Thailand
Cambodia
Laos
Vietnam
Romania
Hungary
Slovakia
Poland
Bosnia and Herzegovina
Croatia
Czech Republic
Slovenia
Austria
Italy
Germany
Luxembourg
Netherlands
Belgium
France
United Kingdom

```
(*For precipitation*)
Plot[{RefineEs[x, 2, 4, {1, 2, 3, 4, 5, 6, 7, 8, 9, 11, 12, 13, 14, 15, 16, 17, 18, 19, 20, 21, 22, 23, 24}, Es1][[3]],
   RefineEs[x, 2, 4, {1, 2, 3, 4, 5, 6, 7, 8, 9, 11, 12, 13, 14, 15, 16, 17, 18, 19, 20, 21, 22, 23, 24}, Es2][[3]],
   RefineEs[x, 2, 4, {1, 2, 3, 4, 5, 6, 7, 8, 9, 11, 12, 13, 14, 15, 16, 17, 18, 19, 20, 21, 22, 23, 24}, Es3][[3]],
   RefineEs[x, 2, 4, {1, 2, 3, 4, 5, 6, 7, 8, 9, 11, 12, 13, 14, 15, 16, 17, 18, 19, 20, 21, 22, 23, 24}, Es4][[3]],
   RefineEs[x, 2, 4, {1, 2, 3, 4, 5, 6, 7, 8, 9, 11, 12, 13, 14, 15, 16, 17, 18, 19, 20, 21, 22, 23, 24}, Es5][[3]],
   RefineEs[x, 2, 4, {1, 2, 3, 4, 5, 6, 7, 8, 9, 11, 12, 13, 14, 15, 16, 17, 18, 19, 20, 21, 22, 23, 24}, Es6][[3]],
   RefineEs[x, 2, 4, {1, 2, 3, 4, 5, 6, 7, 8, 9, 11, 12, 13, 14, 15, 16, 17, 18, 19, 20, 21, 22, 23, 24}, Es7][[3]],
   RefineEs[x, 2, 4, {1, 2, 3, 4, 5, 6, 7, 8, 9, 11, 12, 13, 14, 15, 16, 17, 18, 19, 20, 21, 22, 23, 24}, Es8][[3]],
   RefineEs[x, 2, 4, {1, 2, 3, 4, 5, 6, 7, 8, 9, 11, 12, 13, 14, 15, 16, 17, 18, 19, 20, 21, 22, 23, 24}, Es9][[3]],
   RefineEs[x, 2, 4, {1, 2, 3, 4, 5, 6, 7, 8, 9, 11, 12, 13, 14, 15, 16, 17, 18, 19, 20, 21, 22, 23, 24}, Es10][[3]],
   RefineEs[x, 2, 4, {1, 2, 3, 4, 5, 6, 7, 8, 9, 11, 12, 13, 14, 15, 16, 17, 18, 19, 20, 21, 22, 23, 24}, Es11][[3]],
   RefineEs[x, 2, 4, {1, 2, 3, 4, 5, 6, 7, 8, 9, 11, 12, 13, 14, 15, 16, 17, 18, 19, 20, 21, 22, 23, 24}, Es12][[3]],
   RefineEs[x, 2, 4, {1, 2, 3, 4, 5, 6, 7, 8, 9, 11, 12, 13, 14, 15, 16, 17, 18, 19, 20, 21, 22, 23, 24}, Es13][[3]],
   RefineEs[x, 2, 4, {1, 2, 3, 4, 5, 6, 7, 8, 9, 11, 12, 13, 14, 15, 16, 17, 18, 19, 20, 21, 22, 23, 24}, Es14][[3]],
   RefineEs[x, 2, 4, {1, 2, 3, 4, 5, 6, 7, 8, 9, 11, 12, 13, 14, 15, 16, 17, 18, 19, 20, 21, 22, 23, 24}, Es15][[3]],
   RefineEs[x, 2, 4, {1, 2, 3, 4, 5, 6, 7, 8, 9, 11, 12, 13, 14, 15, 16, 17, 18, 19, 20, 21, 22, 23, 24}, Es16][[3]],
   RefineEs[x, 2, 3, {1, 2, 3, 4, 5, 6, 7, 8, 9, 11, 12, 13, 14, 15, 16, 17, 18, 19, 20, 21, 22, 23, 24}, Es17][[3]],
   RefineEs[x, 2, 3, {1, 2, 3, 4, 5, 6, 7, 8, 9, 11, 12, 13, 14, 15, 16, 17, 18, 19, 20, 21, 22, 23, 24}, Es18][[3]],
   RefineEs[x, 2, 3, {1, 2, 3, 4, 5, 6, 7, 8, 9, 11, 12, 13, 14, 15, 16, 17, 18, 19, 20, 21, 22, 23, 24}, Es19][[3]],
   RefineEs[x, 2, 3, {1, 2, 3, 4, 5, 6, 7, 8, 9, 11, 12, 13, 14, 15, 16, 17, 18, 19, 20, 21, 22, 23, 24}, Es20][[3]],
   RefineEs[x, 2, 3, {1, 2, 3, 4, 5, 6, 7, 8, 9, 11, 12, 13, 14, 15, 16, 17, 18, 19, 20, 21, 22, 23, 24}, Es21][[3]],
   RefineEs[x, 2, 3, {1, 2, 3, 4, 5, 6, 7, 8, 9, 11, 12, 13, 14, 15, 16, 17, 18, 19, 20, 21, 22, 23, 24}, Es22][[3]]},
  {x, 1, 24}, AxesLabel → {"Month Since 2012", Beta}, ImageSize → Large,
  Exclusions → {1, 2, 3, 4, 5, 6, 7, 8, 9, 10, 11, 12, 13, 14, 15, 16, 17, 18, 19, 20, 21, 22, 23, 24},
  PlotLabels → {"Slovenia", "Slovakia", "Poland", "Austria", "Belgium", "Bosnia and Herzegovina",
    "Germany", "France", "Netherlands", "Czech Republic", "Croatia", "Luxembourg", "Romania", "Hungary",
    "Italy", "United Kingdom", "Cambodia", "Laos", "Thailand", "Singapore", "Vietnam", "Malaysia"},
  PlotLegends → {"Slovenia", "Slovakia", "Poland", "Austria", "Belgium", "Bosnia and Herzegovina", "Germany",
    "France", "Netherlands", "Czech Republic", "Croatia", "Luxembourg", "Romania", "Hungary",
    "Italy", "United Kingdom", "Cambodia", "Laos", "Thailand", "Singapore", "Vietnam", "Malaysia"},
  PlotStyle → {ColorData["Rainbow"][0.04], ColorData["Rainbow"][0.09], ColorData["Rainbow"][0.14],
    ColorData["Rainbow"][0.19], ColorData["Rainbow"][0.24], ColorData["Rainbow"][0.29],
    ColorData["Rainbow"][0.34], ColorData["Rainbow"][0.39], ColorData["Rainbow"][0.44], ColorData["Rainbow"][0.49],
    ColorData["Rainbow"][0.54], ColorData["Rainbow"][0.59], ColorData["Rainbow"][0.64], ColorData["Rainbow"][0.69],
    ColorData["Rainbow"][0.74], ColorData["Rainbow"][0.79], ColorData["Rainbow"][0.84], ColorData["Rainbow"][0.89],
    ColorData["Rainbow"][0.76], ColorData["Rainbow"][0.8], ColorData["Rainbow"][0.94], ColorData["Rainbow"][0.99]}]
```

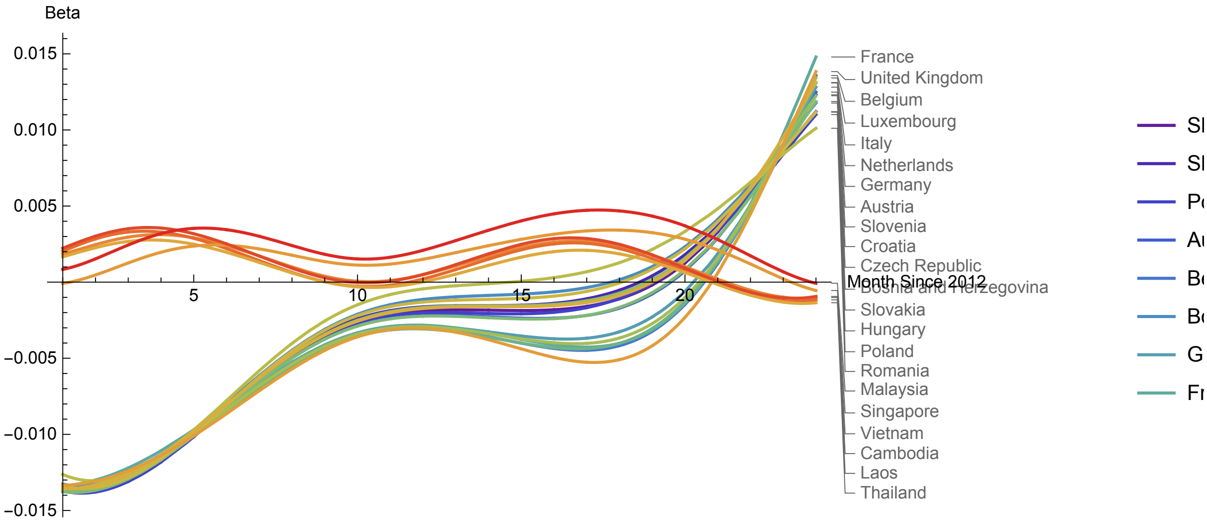

```
(*For RH*)
Plot[{RefineEs[x, 2, 4, {1, 2, 3, 4, 5, 6, 7, 8, 9, 11, 12, 13, 14, 15, 16, 17, 18, 19, 20, 21, 22, 23, 24}, Es1][[4]],
    RefineEs[x, 2, 4, {1, 2, 3, 4, 5, 6, 7, 8, 9, 11, 12, 13, 14, 15, 16, 17, 18, 19, 20, 21, 22, 23, 24}, Es2][[4]],
    RefineEs[x, 2, 4, {1, 2, 3, 4, 5, 6, 7, 8, 9, 11, 12, 13, 14, 15, 16, 17, 18, 19, 20, 21, 22, 23, 24}, Es3][[4]],
    RefineEs[x, 2, 4, {1, 2, 3, 4, 5, 6, 7, 8, 9, 11, 12, 13, 14, 15, 16, 17, 18, 19, 20, 21, 22, 23, 24}, Es4][[4]],
    RefineEs[x, 2, 4, {1, 2, 3, 4, 5, 6, 7, 8, 9, 11, 12, 13, 14, 15, 16, 17, 18, 19, 20, 21, 22, 23, 24}, Es5][[4]],
    RefineEs[x, 2, 4, {1, 2, 3, 4, 5, 6, 7, 8, 9, 11, 12, 13, 14, 15, 16, 17, 18, 19, 20, 21, 22, 23, 24}, Es6][[4]],
    RefineEs[x, 2, 4, {1, 2, 3, 4, 5, 6, 7, 8, 9, 11, 12, 13, 14, 15, 16, 17, 18, 19, 20, 21, 22, 23, 24}, Es7][[4]],
    RefineEs[x, 2, 4, {1, 2, 3, 4, 5, 6, 7, 8, 9, 11, 12, 13, 14, 15, 16, 17, 18, 19, 20, 21, 22, 23, 24}, Es8][[4]],
    RefineEs[x, 2, 4, {1, 2, 3, 4, 5, 6, 7, 8, 9, 11, 12, 13, 14, 15, 16, 17, 18, 19, 20, 21, 22, 23, 24}, Es9][[4]],
    RefineEs[x, 2, 4, {1, 2, 3, 4, 5, 6, 7, 8, 9, 11, 12, 13, 14, 15, 16, 17, 18, 19, 20, 21, 22, 23, 24}, Es10][[4]],
    RefineEs[x, 2, 4, {1, 2, 3, 4, 5, 6, 7, 8, 9, 11, 12, 13, 14, 15, 16, 17, 18, 19, 20, 21, 22, 23, 24}, Es11][[4]],
    RefineEs[x, 2, 4, {1, 2, 3, 4, 5, 6, 7, 8, 9, 11, 12, 13, 14, 15, 16, 17, 18, 19, 20, 21, 22, 23, 24}, Es12][[4]],
    RefineEs[x, 2, 4, {1, 2, 3, 4, 5, 6, 7, 8, 9, 11, 12, 13, 14, 15, 16, 17, 18, 19, 20, 21, 22, 23, 24}, Es13][[4]],
    RefineEs[x, 2, 4, {1, 2, 3, 4, 5, 6, 7, 8, 9, 11, 12, 13, 14, 15, 16, 17, 18, 19, 20, 21, 22, 23, 24}, Es14][[4]],
    RefineEs[x, 2, 4, {1, 2, 3, 4, 5, 6, 7, 8, 9, 11, 12, 13, 14, 15, 16, 17, 18, 19, 20, 21, 22, 23, 24}, Es15][[4]],
    RefineEs[x, 2, 4, {1, 2, 3, 4, 5, 6, 7, 8, 9, 11, 12, 13, 14, 15, 16, 17, 18, 19, 20, 21, 22, 23, 24}, Es16][[4]],
    RefineEs[x, 2, 3, {1, 2, 3, 4, 5, 6, 7, 8, 9, 11, 12, 13, 14, 15, 16, 17, 18, 19, 20, 21, 22, 23, 24}, Es17][[4]],
    RefineEs[x, 2, 3, {1, 2, 3, 4, 5, 6, 7, 8, 9, 11, 12, 13, 14, 15, 16, 17, 18, 19, 20, 21, 22, 23, 24}, Es18][[4]],
    RefineEs[x, 2, 3, {1, 2, 3, 4, 5, 6, 7, 8, 9, 11, 12, 13, 14, 15, 16, 17, 18, 19, 20, 21, 22, 23, 24}, Es19][[4]],
    RefineEs[x, 2, 3, {1, 2, 3, 4, 5, 6, 7, 8, 9, 11, 12, 13, 14, 15, 16, 17, 18, 19, 20, 21, 22, 23, 24}, Es20][[4]],
    RefineEs[x, 2, 3, {1, 2, 3, 4, 5, 6, 7, 8, 9, 11, 12, 13, 14, 15, 16, 17, 18, 19, 20, 21, 22, 23, 24}, Es21][[4]],
    RefineEs[x, 2, 3, {1, 2, 3, 4, 5, 6, 7, 8, 9, 11, 12, 13, 14, 15, 16, 17, 18, 19, 20, 21, 22, 23, 24}, Es22][[4]]},
  {x, 1, 24}, AxesLabel → {"Month Since 2012", Beta}, ImageSize → Large,
  Exclusions → {1, 2, 3, 4, 5, 6, 7, 8, 9, 10, 11, 12, 13, 14, 15, 16, 17, 18, 19, 20, 21, 22, 23, 24},
  PlotLabels → {"Slovenia", "Slovakia", "Poland", "Austria", "Belgium", "Bosnia and Herzegovina",
     "Germany", "France", "Netherlands", "Czech Republic", "Croatia", "Luxembourg", "Romania", "Hungary",
     "Italy", "United Kingdom", "Cambodia", "Laos", "Thailand", "Singapore", "Vietnam", "Malaysia"},
  PlotLegends → {"Slovenia", "Slovakia", "Poland", "Austria", "Belgium", "Bosnia and Herzegovina", "Germany",
     "France", "Netherlands", "Czech Republic", "Croatia", "Luxembourg", "Romania", "Hungary",
     "Italy", "United Kingdom", "Cambodia", "Laos", "Thailand", "Singapore", "Vietnam", "Malaysia"},
  PlotStyle → {ColorData["Rainbow"][0.04], ColorData["Rainbow"][0.09], ColorData["Rainbow"][0.14],
     ColorData["Rainbow"][0.19], ColorData["Rainbow"][0.24], ColorData["Rainbow"][0.29],
     ColorData["Rainbow"][0.34], ColorData["Rainbow"][0.39], ColorData["Rainbow"][0.44], ColorData["Rainbow"][0.49],
     ColorData["Rainbow"][0.54], ColorData["Rainbow"][0.59], ColorData["Rainbow"][0.64], ColorData["Rainbow"][0.69],
     ColorData["Rainbow"][0.74], ColorData["Rainbow"][0.79], ColorData["Rainbow"][0.84], ColorData["Rainbow"][0.89],
     ColorData["Rainbow"][0.76], ColorData["Rainbow"][0.8], ColorData["Rainbow"][0.94], ColorData["Rainbow"][0.99]}]
```

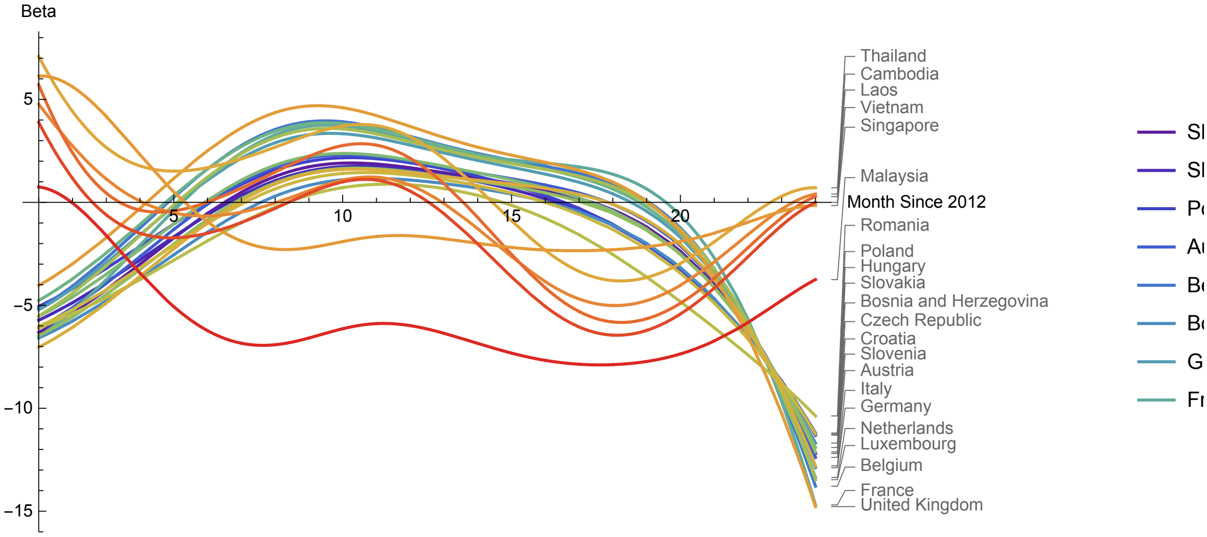

We can also draw the geographical heat map to see the spatial difference of these coefficients
Please first run the following code

```
TemCoun1[x_] := GeoRegionValuePlot[{ Slovenia (country) → ColorData["Rainbow"][
    Rescale[RefineEs[x, 2, 4, {1, 2, 3, 4, 5, 6, 7, 8, 9, 11, 12, 13, 14, 15, 16, 17, 18, 19, 20, 21, 22, 23, 24}, Es1][[2]],
     {-0.3, 0.3}, {0, 1}]], Slovakia (country) → ColorData["Rainbow"][
    Rescale[RefineEs[x, 2, 4, {1, 2, 3, 4, 5, 6, 7, 8, 9, 11, 12, 13, 14, 15, 16, 17, 18, 19, 20, 21, 22, 23, 24}, Es2][[2]],
     {-0.3, 0.3}, {0, 1}]], Poland (country) → ColorData["Rainbow"][
    Rescale[RefineEs[x, 2, 4, {1, 2, 3, 4, 5, 6, 7, 8, 9, 11, 12, 13, 14, 15, 16, 17, 18, 19, 20, 21, 22, 23, 24}, Es3][[2]],
     {-0.3, 0.3}, {0, 1}]], Austria (country) → ColorData["Rainbow"][
    Rescale[RefineEs[x, 2, 4, {1, 2, 3, 4, 5, 6, 7, 8, 9, 11, 12, 13, 14, 15, 16, 17, 18, 19, 20, 21, 22, 23, 24}, Es4][[2]],
     {-0.3, 0.3}, {0, 1}]], Belgium (country) -> ColorData["Rainbow"][Rescale[
     RefineEs[x, 2, 4, {1, 2, 3, 4, 5, 6, 7, 8, 9, 11, 12, 13, 14, 15, 16, 17, 18, 19, 20, 21, 22, 23, 24}, Es5][[2]], {-0.3, 0.3}, {0, 1}]],
   Bosnia and Herzegovina (country) -> ColorData["Rainbow"][Rescale[
     RefineEs[x, 2, 4, {1, 2, 3, 4, 5, 6, 7, 8, 9, 11, 12, 13, 14, 15, 16, 17, 18, 19, 20, 21, 22, 23, 24}, Es6][[2]], {-0.3, 0.3}, {0, 1}]],
   Germany (country) -> ColorData["Rainbow"][Rescale[
     RefineEs[x, 2, 4, {1, 2, 3, 4, 5, 6, 7, 8, 9, 11, 12, 13, 14, 15, 16, 17, 18, 19, 20, 21, 22, 23, 24}, Es7][[2]], {-0.3, 0.3}, {0, 1}]],
   France (country) -> ColorData["Rainbow"][Rescale[
     RefineEs[x, 2, 4, {1, 2, 3, 4, 5, 6, 7, 8, 9, 11, 12, 13, 14, 15, 16, 17, 18, 19, 20, 21, 22, 23, 24}, Es8][[2]], {-0.3, 0.3}, {0, 1}]],
   Netherlands (country) -> ColorData["Rainbow"][Rescale[
     RefineEs[x, 2, 4, {1, 2, 3, 4, 5, 6, 7, 8, 9, 11, 12, 13, 14, 15, 16, 17, 18, 19, 20, 21, 22, 23, 24}, Es9][[2]], {-0.3, 0.3}, {0, 1}]],
   Czech Republic (country) -> ColorData["Rainbow"][
    Rescale[RefineEs[x, 2, 4, {1, 2, 3, 4, 5, 6, 7, 8, 9, 11, 12, 13, 14, 15, 16, 17, 18, 19, 20, 21, 22, 23, 24}, Es10][[2]],
     {-0.3, 0.3}, {0, 1}]], Croatia (country) -> ColorData["Rainbow"][
    Rescale[RefineEs[x, 2, 4, {1, 2, 3, 4, 5, 6, 7, 8, 9, 11, 12, 13, 14, 15, 16, 17, 18, 19, 20, 21, 22, 23, 24}, Es11][[2]],
     {-0.3, 0.3}, {0, 1}]], Luxembourg (country) -> ColorData["Rainbow"][
    Rescale[RefineEs[x, 2, 4, {1, 2, 3, 4, 5, 6, 7, 8, 9, 11, 12, 13, 14, 15, 16, 17, 18, 19, 20, 21, 22, 23, 24}, Es12][[2]],
     {-0.3, 0.3}, {0, 1}]], Romania (country) -> ColorData["Rainbow"][
    Rescale[RefineEs[x, 2, 4, {1, 2, 3, 4, 5, 6, 7, 8, 9, 11, 12, 13, 14, 15, 16, 17, 18, 19, 20, 21, 22, 23, 24}, Es13][[2]],
     {-0.3, 0.3}, {0, 1}]], Hungary (country) -> ColorData["Rainbow"][
    Rescale[RefineEs[x, 2, 4, {1, 2, 3, 4, 5, 6, 7, 8, 9, 11, 12, 13, 14, 15, 16, 17, 18, 19, 20, 21, 22, 23, 24}, Es14][[2]],
     {-0.3, 0.3}, {0, 1}]], Italy (country) -> ColorData["Rainbow"][Rescale[
     RefineEs[x, 2, 4, {1, 2, 3, 4, 5, 6, 7, 8, 9, 11, 12, 13, 14, 15, 16, 17, 18, 19, 20, 21, 22, 23, 24}, Es15][[2]], {-0.3, 0.3}, {0, 1}]],
   United Kingdom (country) -> ColorData["Rainbow"][Rescale[RefineEs[x, 2, 4,
        {1, 2, 3, 4, 5, 6, 7, 8, 9, 11, 12, 13, 14, 15, 16, 17, 18, 19, 20, 21, 22, 23, 24}, Es16][[2]], {-0.3, 0.3}, {0, 1}]]}]
TemCoun2[x_] := GeoRegionValuePlot[{ Cambodia (country) → ColorData["Rainbow"][
    Rescale[RefineEs[x, 2, 3, {1, 2, 3, 4, 5, 6, 7, 8, 9, 11, 12, 13, 14, 15, 16, 17, 18, 19, 20, 21, 22, 23, 24}, Es17][[2]],
     {-0.3, 0.3}, {0, 1}]], Laos (country) → ColorData["Rainbow"][
    Rescale[RefineEs[x, 2, 3, {1, 2, 3, 4, 5, 6, 7, 8, 9, 11, 12, 13, 14, 15, 16, 17, 18, 19, 20, 21, 22, 23, 24}, Es18][[2]],
     {-0.3, 0.3}, {0, 1}]], Thailand (country) → ColorData["Rainbow"][
    Rescale[RefineEs[x, 2, 3, {1, 2, 3, 4, 5, 6, 7, 8, 9, 11, 12, 13, 14, 15, 16, 17, 18, 19, 20, 21, 22, 23, 24}, Es19][[2]],
     {-0.3, 0.3}, {0, 1}]], Singapore (country) → ColorData["Rainbow"][
```

```
Rescale[RefineEs[x, 2, 3, {1, 2, 3, 4, 5, 6, 7, 8, 9, 11, 12, 13, 14, 15, 16, 17, 18, 19, 20, 21, 22, 23, 24}, Es20][[2]],
  {−0.3, 0.3}, {0, 1}]], Vietnam (country) −> ColorData["Rainbow"][Rescale[
  RefineEs[x, 2, 3, {1, 2, 3, 4, 5, 6, 7, 8, 9, 11, 12, 13, 14, 15, 16, 17, 18, 19, 20, 21, 22, 23, 24}, Es21][[2]], {−0.3, 0.3}, {0, 1}]],
Malaysia (country) −> ColorData["Rainbow"][Rescale[RefineEs[x, 2, 3,
    {1, 2, 3, 4, 5, 6, 7, 8, 9, 11, 12, 13, 14, 15, 16, 17, 18, 19, 20, 21, 22, 23, 24}, Es22][[2]], {−0.3, 0.3}, {0, 1}]]}]
PreC1[x_] := GeoRegionValuePlot[{Slovenia (country) → ColorData["Rainbow"][
    Rescale[RefineEs[x, 2, 4, {1, 2, 3, 4, 5, 6, 7, 8, 9, 11, 12, 13, 14, 15, 16, 17, 18, 19, 20, 21, 22, 23, 24}, Es1][[3]],
     {−0.015, 0.015}, {0, 1}]], Slovakia (country) → ColorData["Rainbow"][
    Rescale[RefineEs[x, 2, 4, {1, 2, 3, 4, 5, 6, 7, 8, 9, 11, 12, 13, 14, 15, 16, 17, 18, 19, 20, 21, 22, 23, 24}, Es2][[3]],
     {−0.015, 0.015}, {0, 1}]], Poland (country) → ColorData["Rainbow"][
    Rescale[RefineEs[x, 2, 4, {1, 2, 3, 4, 5, 6, 7, 8, 9, 11, 12, 13, 14, 15, 16, 17, 18, 19, 20, 21, 22, 23, 24}, Es3][[3]],
     {−0.015, 0.015}, {0, 1}]], Austria (country) → ColorData["Rainbow"][
    Rescale[RefineEs[x, 2, 4, {1, 2, 3, 4, 5, 6, 7, 8, 9, 11, 12, 13, 14, 15, 16, 17, 18, 19, 20, 21, 22, 23, 24}, Es4][[3]],
     {−0.015, 0.015}, {0, 1}]], Belgium (country) −> ColorData["Rainbow"][
    Rescale[RefineEs[x, 2, 4, {1, 2, 3, 4, 5, 6, 7, 8, 9, 11, 12, 13, 14, 15, 16, 17, 18, 19, 20, 21, 22, 23, 24}, Es5][[3]],
     {−0.015, 0.015}, {0, 1}]], Bosnia and Herzegovina (country) −> ColorData["Rainbow"][
    Rescale[RefineEs[x, 2, 4, {1, 2, 3, 4, 5, 6, 7, 8, 9, 11, 12, 13, 14, 15, 16, 17, 18, 19, 20, 21, 22, 23, 24}, Es6][[3]],
     {−0.015, 0.015}, {0, 1}]], Germany (country) −> ColorData["Rainbow"][
    Rescale[RefineEs[x, 2, 4, {1, 2, 3, 4, 5, 6, 7, 8, 9, 11, 12, 13, 14, 15, 16, 17, 18, 19, 20, 21, 22, 23, 24}, Es7][[3]],
     {−0.015, 0.015}, {0, 1}]], France (country) −> ColorData["Rainbow"][
    Rescale[RefineEs[x, 2, 4, {1, 2, 3, 4, 5, 6, 7, 8, 9, 11, 12, 13, 14, 15, 16, 17, 18, 19, 20, 21, 22, 23, 24}, Es8][[3]],
     {−0.015, 0.015}, {0, 1}]], Netherlands (country) −> ColorData["Rainbow"][
    Rescale[RefineEs[x, 2, 4, {1, 2, 3, 4, 5, 6, 7, 8, 9, 11, 12, 13, 14, 15, 16, 17, 18, 19, 20, 21, 22, 23, 24}, Es9][[3]],
     {−0.015, 0.015}, {0, 1}]], Czech Republic (country) −> ColorData["Rainbow"][
    Rescale[RefineEs[x, 2, 4, {1, 2, 3, 4, 5, 6, 7, 8, 9, 11, 12, 13, 14, 15, 16, 17, 18, 19, 20, 21, 22, 23, 24}, Es10][[3]],
     {−0.015, 0.015}, {0, 1}]], Croatia (country) −> ColorData["Rainbow"][
    Rescale[RefineEs[x, 2, 4, {1, 2, 3, 4, 5, 6, 7, 8, 9, 11, 12, 13, 14, 15, 16, 17, 18, 19, 20, 21, 22, 23, 24}, Es11][[3]],
     {−0.015, 0.015}, {0, 1}]], Luxembourg (country) −> ColorData["Rainbow"][
    Rescale[RefineEs[x, 2, 4, {1, 2, 3, 4, 5, 6, 7, 8, 9, 11, 12, 13, 14, 15, 16, 17, 18, 19, 20, 21, 22, 23, 24}, Es12][[3]],
     {−0.015, 0.015}, {0, 1}]], Romania (country) −> ColorData["Rainbow"][
    Rescale[RefineEs[x, 2, 4, {1, 2, 3, 4, 5, 6, 7, 8, 9, 11, 12, 13, 14, 15, 16, 17, 18, 19, 20, 21, 22, 23, 24}, Es13][[3]],
     {−0.015, 0.015}, {0, 1}]], Hungary (country) −> ColorData["Rainbow"][
    Rescale[RefineEs[x, 2, 4, {1, 2, 3, 4, 5, 6, 7, 8, 9, 11, 12, 13, 14, 15, 16, 17, 18, 19, 20, 21, 22, 23, 24}, Es14][[3]],
     {−0.015, 0.015}, {0, 1}]], Italy (country) −> ColorData["Rainbow"][
    Rescale[RefineEs[x, 2, 4, {1, 2, 3, 4, 5, 6, 7, 8, 9, 11, 12, 13, 14, 15, 16, 17, 18, 19, 20, 21, 22, 23, 24}, Es15][[3]],
     {−0.015, 0.015}, {0, 1}]], United Kingdom (country) −> ColorData["Rainbow"][
    Rescale[RefineEs[x, 2, 4, {1, 2, 3, 4, 5, 6, 7, 8, 9, 11, 12, 13, 14, 15, 16, 17, 18, 19, 20, 21, 22, 23, 24}, Es16][[3]],
     {−0.015, 0.015}, {0, 1}]]}]
PreC2[x_] := GeoRegionValuePlot[{Cambodia (country) → ColorData["Rainbow"][
    Rescale[RefineEs[x, 2, 3, {1, 2, 3, 4, 5, 6, 7, 8, 9, 11, 12, 13, 14, 15, 16, 17, 18, 19, 20, 21, 22, 23, 24}, Es17][[3]],
     {−0.015, 0.015}, {0, 1}]], Laos (country) → ColorData["Rainbow"][
    Rescale[RefineEs[x, 2, 3, {1, 2, 3, 4, 5, 6, 7, 8, 9, 11, 12, 13, 14, 15, 16, 17, 18, 19, 20, 21, 22, 23, 24}, Es18][[3]],
```

```
  {−0.015, 0.015}, {0, 1}]], Thailand (country) → ColorData["Rainbow"][
 Rescale[RefineEs[x, 2, 3, {1, 2, 3, 4, 5, 6, 7, 8, 9, 11, 12, 13, 14, 15, 16, 17, 18, 19, 20, 21, 22, 23, 24}, Es19][[3]],
  {−0.015, 0.015}, {0, 1}]], Singapore (country) → ColorData["Rainbow"][
 Rescale[RefineEs[x, 2, 3, {1, 2, 3, 4, 5, 6, 7, 8, 9, 11, 12, 13, 14, 15, 16, 17, 18, 19, 20, 21, 22, 23, 24}, Es20][[3]],
  {−0.015, 0.015}, {0, 1}]], Vietnam (country) −> ColorData["Rainbow"][
 Rescale[RefineEs[x, 2, 3, {1, 2, 3, 4, 5, 6, 7, 8, 9, 11, 12, 13, 14, 15, 16, 17, 18, 19, 20, 21, 22, 23, 24}, Es21][[3]],
  {−0.015, 0.015}, {0, 1}]], Malaysia (country) −> ColorData["Rainbow"][
 Rescale[RefineEs[x, 2, 3, {1, 2, 3, 4, 5, 6, 7, 8, 9, 11, 12, 13, 14, 15, 16, 17, 18, 19, 20, 21, 22, 23, 24}, Es22][[3]],
  {−0.015, 0.015}, {0, 1}]]}]
AHC1[x_] := GeoRegionValuePlot[{ Slovenia (country) → ColorData["Rainbow"][Rescale[
  RefineEs[x, 2, 4, {1, 2, 3, 4, 5, 6, 7, 8, 9, 11, 12, 13, 14, 15, 16, 17, 18, 19, 20, 21, 22, 23, 24}, Es1][[4]], {−10, 8}, {0, 1}]],
 Slovakia (country) → ColorData["Rainbow"][Rescale[RefineEs[x, 2, 4, {1, 2, 3, 4, 5, 6, 7, 8, 9, 11, 12, 13, 14, 15,
    16, 17, 18, 19, 20, 21, 22, 23, 24}, Es2][[4]], {−10, 8}, {0, 1}]], Poland (country) → ColorData["Rainbow"][
 Rescale[RefineEs[x, 2, 4, {1, 2, 3, 4, 5, 6, 7, 8, 9, 11, 12, 13, 14, 15, 16, 17, 18, 19, 20, 21, 22, 23, 24}, Es3][[4]],
  {−10, 8}, {0, 1}]], Austria (country) → ColorData["Rainbow"][
 Rescale[RefineEs[x, 2, 4, {1, 2, 3, 4, 5, 6, 7, 8, 9, 11, 12, 13, 14, 15, 16, 17, 18, 19, 20, 21, 22, 23, 24}, Es4][[4]],
  {−10, 8}, {0, 1}]], Belgium (country) −> ColorData["Rainbow"][Rescale[
  RefineEs[x, 2, 4, {1, 2, 3, 4, 5, 6, 7, 8, 9, 11, 12, 13, 14, 15, 16, 17, 18, 19, 20, 21, 22, 23, 24}, Es5][[4]], {−10, 8}, {0, 1}]],
 Bosnia and Herzegovina (country) −> ColorData["Rainbow"][Rescale[
  RefineEs[x, 2, 4, {1, 2, 3, 4, 5, 6, 7, 8, 9, 11, 12, 13, 14, 15, 16, 17, 18, 19, 20, 21, 22, 23, 24}, Es6][[4]], {−10, 8}, {0, 1}]],
 Germany (country) −> ColorData["Rainbow"][Rescale[
  RefineEs[x, 2, 4, {1, 2, 3, 4, 5, 6, 7, 8, 9, 11, 12, 13, 14, 15, 16, 17, 18, 19, 20, 21, 22, 23, 24}, Es7][[4]], {−10, 8}, {0, 1}]],
 France (country) −> ColorData["Rainbow"][Rescale[
  RefineEs[x, 2, 4, {1, 2, 3, 4, 5, 6, 7, 8, 9, 11, 12, 13, 14, 15, 16, 17, 18, 19, 20, 21, 22, 23, 24}, Es8][[4]], {−10, 8}, {0, 1}]],
 Netherlands (country) −> ColorData["Rainbow"][Rescale[
  RefineEs[x, 2, 4, {1, 2, 3, 4, 5, 6, 7, 8, 9, 11, 12, 13, 14, 15, 16, 17, 18, 19, 20, 21, 22, 23, 24}, Es9][[4]], {−10, 8}, {0, 1}]],
 Czech Republic (country) −> ColorData["Rainbow"][Rescale[
  RefineEs[x, 2, 4, {1, 2, 3, 4, 5, 6, 7, 8, 9, 11, 12, 13, 14, 15, 16, 17, 18, 19, 20, 21, 22, 23, 24}, Es10][[4]], {−10, 8}, {0, 1}]],
 Croatia (country) −> ColorData["Rainbow"][Rescale[
  RefineEs[x, 2, 4, {1, 2, 3, 4, 5, 6, 7, 8, 9, 11, 12, 13, 14, 15, 16, 17, 18, 19, 20, 21, 22, 23, 24}, Es11][[4]], {−10, 8}, {0, 1}]],
 Luxembourg (country) −> ColorData["Rainbow"][Rescale[
  RefineEs[x, 2, 4, {1, 2, 3, 4, 5, 6, 7, 8, 9, 11, 12, 13, 14, 15, 16, 17, 18, 19, 20, 21, 22, 23, 24}, Es12][[4]], {−10, 8}, {0, 1}]],
 Romania (country) −> ColorData["Rainbow"][Rescale[RefineEs[x, 2, 4, {1, 2, 3, 4, 5, 6, 7, 8, 9, 11, 12, 13, 14, 15,
    16, 17, 18, 19, 20, 21, 22, 23, 24}, Es13][[4]], {−10, 8}, {0, 1}]], Hungary (country) −> ColorData["Rainbow"][
 Rescale[RefineEs[x, 2, 4, {1, 2, 3, 4, 5, 6, 7, 8, 9, 11, 12, 13, 14, 15, 16, 17, 18, 19, 20, 21, 22, 23, 24}, Es14][[4]],
  {−10, 8}, {0, 1}]], Italy (country) −> ColorData["Rainbow"][Rescale[
  RefineEs[x, 2, 4, {1, 2, 3, 4, 5, 6, 7, 8, 9, 11, 12, 13, 14, 15, 16, 17, 18, 19, 20, 21, 22, 23, 24}, Es15][[4]], {−10, 8}, {0, 1}]],
 United Kingdom (country) −> ColorData["Rainbow"][Rescale[
  RefineEs[x, 2, 4, {1, 2, 3, 4, 5, 6, 7, 8, 9, 11, 12, 13, 14, 15, 16, 17, 18, 19, 20, 21, 22, 23, 24}, Es16][[4]], {−10, 8}, {0, 1}]]}]
AHC2[x_] := GeoRegionValuePlot[{ Cambodia (country) → ColorData["Rainbow"][
 Rescale[RefineEs[x, 2, 3, {1, 2, 3, 4, 5, 6, 7, 8, 9, 11, 12, 13, 14, 15, 16, 17, 18, 19, 20, 21, 22, 23, 24}, Es17][[4]],
```

```
{−10, 8}, {0, 1}]], Laos (country) → ColorData["Rainbow"][
Rescale[RefineEs[x, 2, 3, {1, 2, 3, 4, 5, 6, 7, 8, 9, 11, 12, 13, 14, 15, 16, 17, 18, 19, 20, 21, 22, 23, 24}, Es18][[4]],
{−10, 8}, {0, 1}]], Thailand (country) → ColorData["Rainbow"][Rescale[
RefineEs[x, 2, 3, {1, 2, 3, 4, 5, 6, 7, 8, 9, 11, 12, 13, 14, 15, 16, 17, 18, 19, 20, 21, 22, 23, 24}, Es19][[4]], {−10, 8}, {0, 1}]],
Singapore (country) → ColorData["Rainbow"][Rescale[
RefineEs[x, 2, 3, {1, 2, 3, 4, 5, 6, 7, 8, 9, 11, 12, 13, 14, 15, 16, 17, 18, 19, 20, 21, 22, 23, 24}, Es20][[4]], {−10, 8}, {0, 1}]],
Vietnam (country) −> ColorData["Rainbow"][Rescale[
RefineEs[x, 2, 3, {1, 2, 3, 4, 5, 6, 7, 8, 9, 11, 12, 13, 14, 15, 16, 17, 18, 19, 20, 21, 22, 23, 24}, Es21][[4]], {−10, 8}, {0, 1}]],
Malaysia (country) −> ColorData["Rainbow"][Rescale[
RefineEs[x, 2, 3, {1, 2, 3, 4, 5, 6, 7, 8, 9, 11, 12, 13, 14, 15, 16, 17, 18, 19, 20, 21, 22, 23, 24}, Es22][[4]], {−10, 8}, {0, 1}]]}]
```

We have defined six function above. “TemCoun1”, “PreC1” and “AHC1” are functions of temperature, precipitation and RH for European countries, the other three are functions for Southeast Asia countries

We use function “BarLegend” to get colorbar, we use function “TemCoun1” to draw the heat map of temperature in European countries

```
(*Colorbar for temperature*)
BarLegend[{"Rainbow", {−0.3, 0.3}}]
```

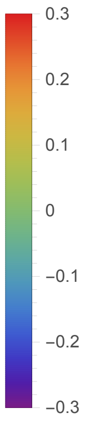


```
(*You can use function "Manipulate" to see the animation of the heat map!*)
Manipulate[TemCoun1[a], {a, 1, 24}]
```

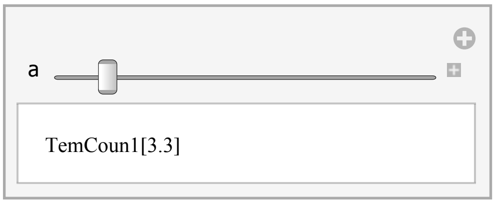

(*For temperature in Jan−2012, Europe*)
TemCoun1[1.0]

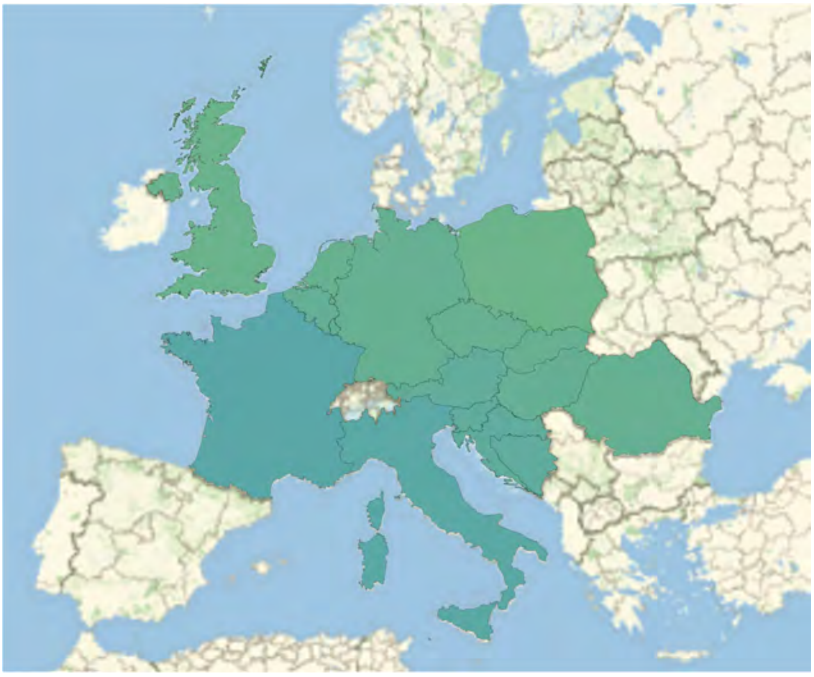

(*For temperature in Jul−2012, Europe*)
TemCoun1[7.0]

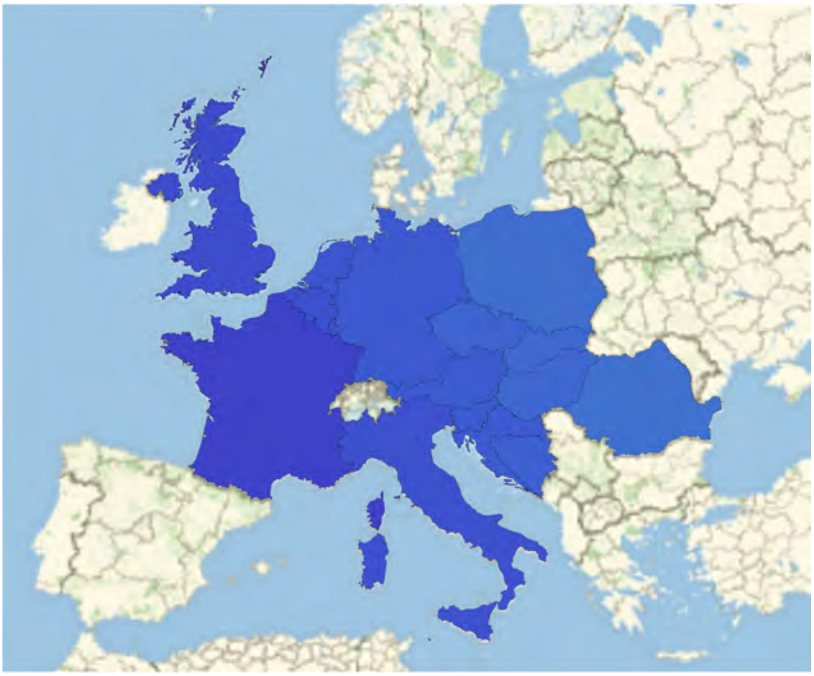

(*For temperature in Jan−2013, Europe*)
TemCoun1[13.0]

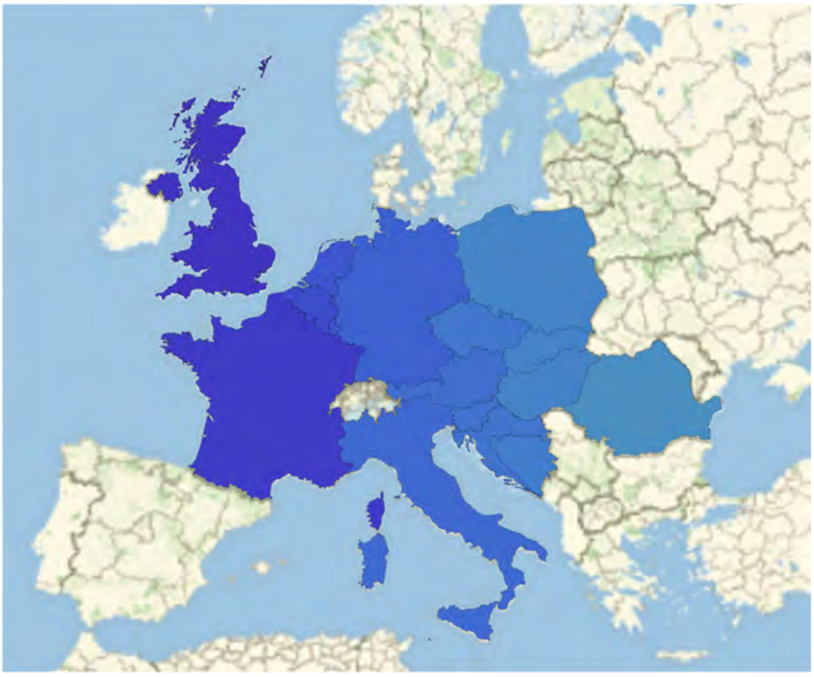

(*For temperature in Jul−2013, Europe*)
TemCoun1[19.0]

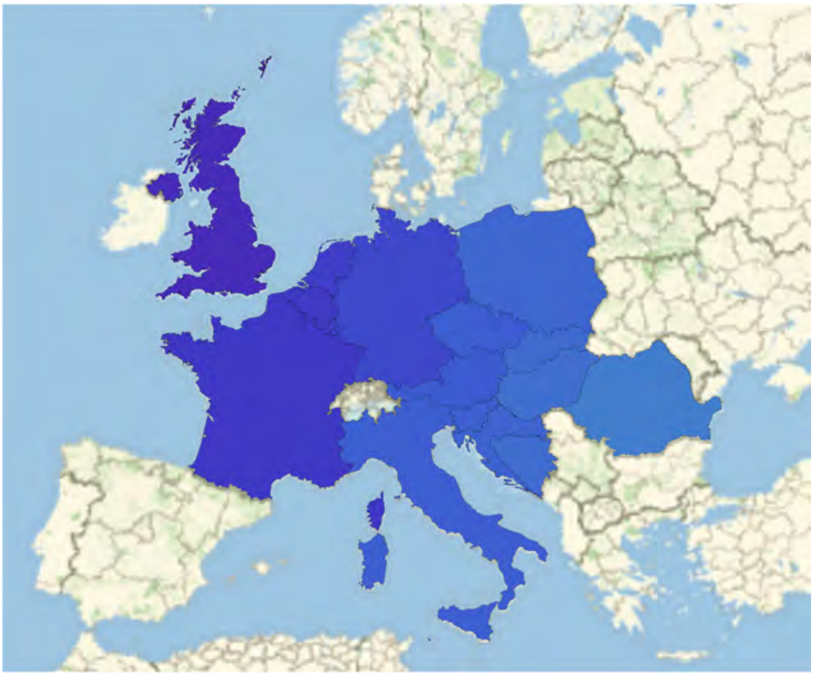

(*Same process can be applied to heat map of other factor.*)

```
(*For temperature of Southeast asia*)
Manipulate[TemCoun2[a], {a, 1, 25}]
```

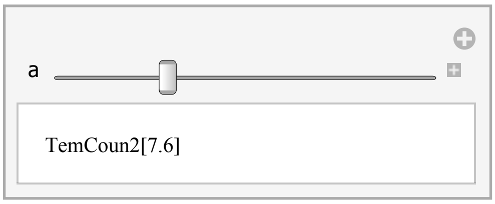


```
(*colorbar for precipitation*)
BarLegend[{"Rainbow", {−0.015, 0.015}}]
```

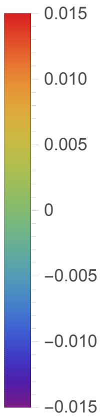


```
(*For precipitation of European countries*)
Manipulate[PreC1[a], {a, 1, 25}]
```

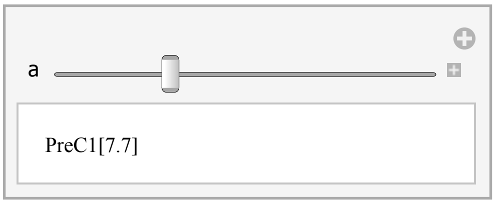


```
(*For precipitation of Southeast asia*)
Manipulate[PreC2[a], {a, 1, 25}]
```

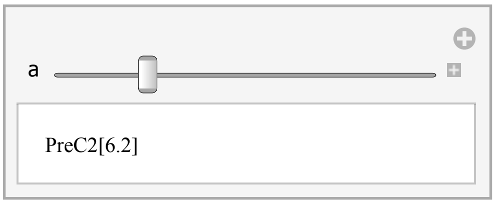

(*colorbar for RH*)
BarLegend[{"Rainbow", {−10, 8}}]

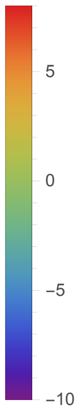

(*For RH of European countries*)
Manipulate[AHC1[a], {a, 1, 25}]

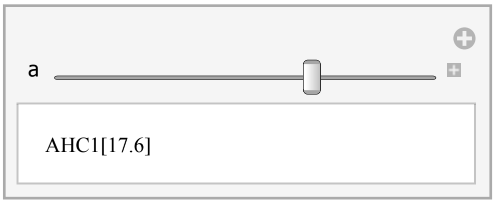

(*For RH of Southeast asia*)
Manipulate[AHC2[a], {a, 1, 25}]

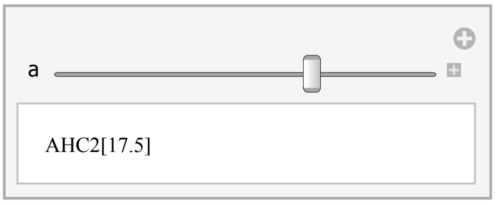

Supplement: Supplementary file 2 — Supplementary Codes [file 41598_2018_19772_MOESM2_ESM.pdf]
